# Stereochemical Rules Govern the Soft Self‐Assembly of Achiral Compounds: Understanding the Heliconical Liquid‐Crystalline Phases of Bent‐Core Mesogens

**DOI:** 10.1002/chem.201904871

**Published:** 2020-02-28

**Authors:** Anne Lehmann, Mohamed Alaasar, Marco Poppe, Silvio Poppe, Marko Prehm, Mamatha Nagaraj, Sithara P. Sreenilayam, Yuri P. Panarin, Jagdish K. Vij, Carsten Tschierske

**Affiliations:** ^1^ Department of Chemistry Martin Luther University Halle-Wittenberg Kurt Mothes Str. 2 06120 Halle (Saale) Germany; ^2^ Department of Chemistry Cairo University 12613 Giza Egypt; ^3^ Department of Electronic and Electrical Engineering Trinity College, Dublin, The University of Dublin Dublin 2 Ireland

**Keywords:** chirality, de Vries phases, ferroelectricity, helical structures, liquid crystals

## Abstract

A series of bent‐shaped 4‐cyanoresorcinol bisterephthalates is reported. Some of these achiral compounds spontaneously form a short‐pitch heliconical lamellar liquid‐crystalline phase with incommensurate 3‐layer pitch and the helix axis parallel to the layer normal. It is observed at the paraelectric‐(anti)ferroelectric transition, if it coincides with the transition from random to uniform tilt and with the transition from anticlinic to synclinic tilt correlation of the molecules in the layers of the developing tilted smectic phase. For compounds with long chains the heliconical phase is only field‐induced, but once formed it is stable in a distinct temperature range, even after switching off the field. The presence of the helix changes the phase properties and the switching mechanism from the naturally preferred rotation around the molecular long axis, which reverses the chirality, to a precession on a cone, which retains the chirality. These observations are explained by diastereomeric relations between two coexisting modes of superstructural chirality. One is the layer chirality, resulting from the combination of tilt and polar order, and the other one is the helical twist evolving between the layers. At lower temperature the helical structure is replaced by a non‐tilted and ferreoelectric switching lamellar phase, providing an alternative non‐chiral way for the transition from anticlinic to synclinic tilt.

## Graphical abstract

**Mirror symmetry breaking in fluids**: Achiral bent‐shaped molecules form a spontaneous or field induced short‐pitch heliconical lamellar liquid‐crystalline phase. The combination of the helix with the superstructural layer chirality leads to diastereomeric pairs and changes the switching mechanism from the naturally preferred rotation around the molecular long axis, inverting the layer chirality, to a precession on a cone, retaining the chirality.

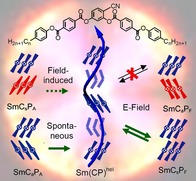

## Introduction

In biological systems, the chirality of proteins, carbohydrates and DNA have developed into stable systems from initially achiral sources.^1^ Formation of helical superstructures^2^, ^3^, ^4^, ^5^, ^6^ chirality amplification^7^, ^8^ and spontaneous mirror symmetry breaking, are nowadays well documented for solid state and crystalline assembles.^2^ In contrast, for the fluid state spontaneous mirror symmetry breaking was assumed to be impossible due to the significant contribution of the mixing entropy; nevertheless, it was experimentally observed recently.^9^ As biological chirality has presumably developed in the fluid state an understanding of the symmetry breaking in fluids is of significant importance.

**Figure 1:**
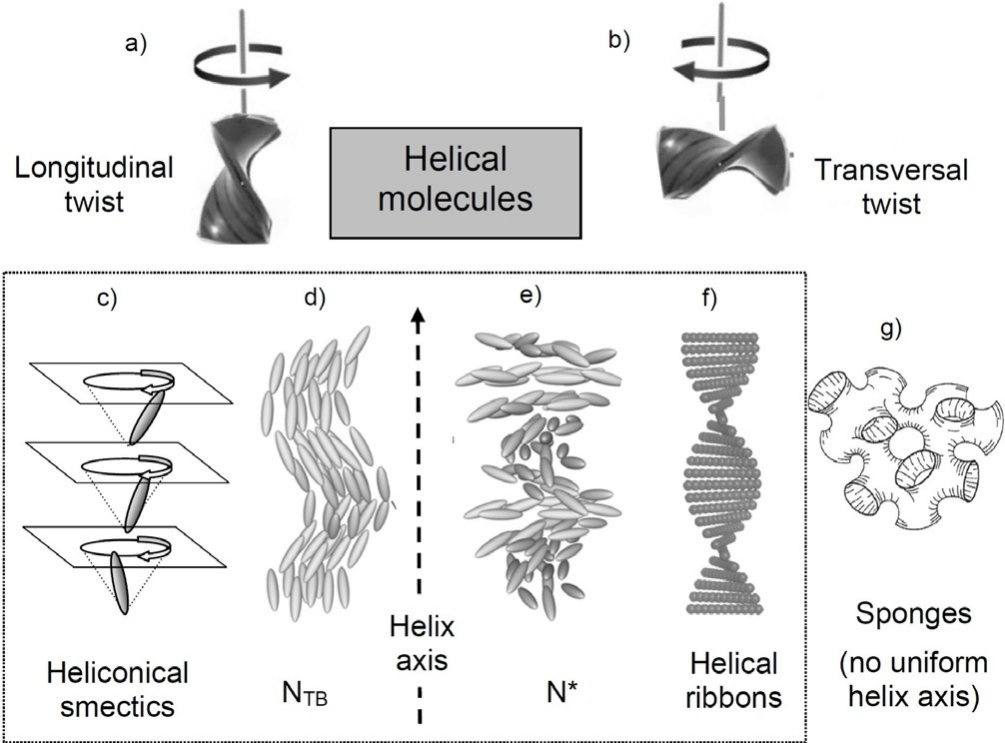
Chirality and mirror symmetry breaking in LC soft matter formed by permanently and transiently chiral rod‐like or bent molecules; a) longitudinal coupling of the helical twist leads to: c) heliconical smectic phases (SmC_α_, SmC_TB_, etc.) or d) the twist‐bend nematic phases (N_TB_), whereas b) transversal helix coupling leads to e) chiral nematic phases (N*), f) helical nano‐filament phases (HNF) or g) the sponge‐like dark conglomerate phases.

Liquid crystals (LCs) are fluid systems with long range orientational or positional order, thus representing simple model systems for studying the fundamental concepts of symmetry breaking at the cross‐over from long‐range order in crystalline solids to short‐range order in the isotropic liquids.^10^, ^11^, ^12^ Moreover, LCs are of significant technological importance for displays and numerous non‐display applications.^11^ Especially, helical superstructures formed by LCs arose interest as switchable optical and photonic materials for tunable lasers, circular polarized emitters and a number of other technological applications.^13^ It is known that chirality in LC systems can result from a permanent molecular chirality, providing a helical twist of molecular conformation with a preferred twist sense determined by the sense of permanent chirality, and thus leading to helical LC phases with fixed helix sense (Figure 1). In the nematic phases, involving only orientational order, the helicity of the involved molecules induces a helical twist between the molecular long axes (transversal twist correlation, Figure 1 b) leading to the chiral nematic phases (N*, see Figure 1 e).^14^ However, in the smectic phases with additional positional order, provided by the layer periodicity, the helical twist distorts the formation of layers and leads to a series of chirality frustrated LC phases (Figure 1 f, g).^14^, ^15^, ^16^ If the helical twist develops longitudinally, that is, between the short molecular axes (Figure 1 a), then undistorted layers can be retained and in this case heliconical lamellar phases, having the helix parallel to the layer normal, arise (Figure 1 c). Long pitch heliconical smectic phases are known for chiral rod‐like molecules in the synclinic SmC_s_* and the anticlinic SmC_a_* phases. However, the formation of short pitch heliconical LC phases (SmC_α_*, SmC_FI_*) requires a „strong“ permanent chirality and a high enantiomeric purity of the involved molecules and it occurs at the transition between different phases, either SmA* and SmC* or SmC_s_* and SmC_a_*.^14^, ^17^, ^18^

**Figure 2:**
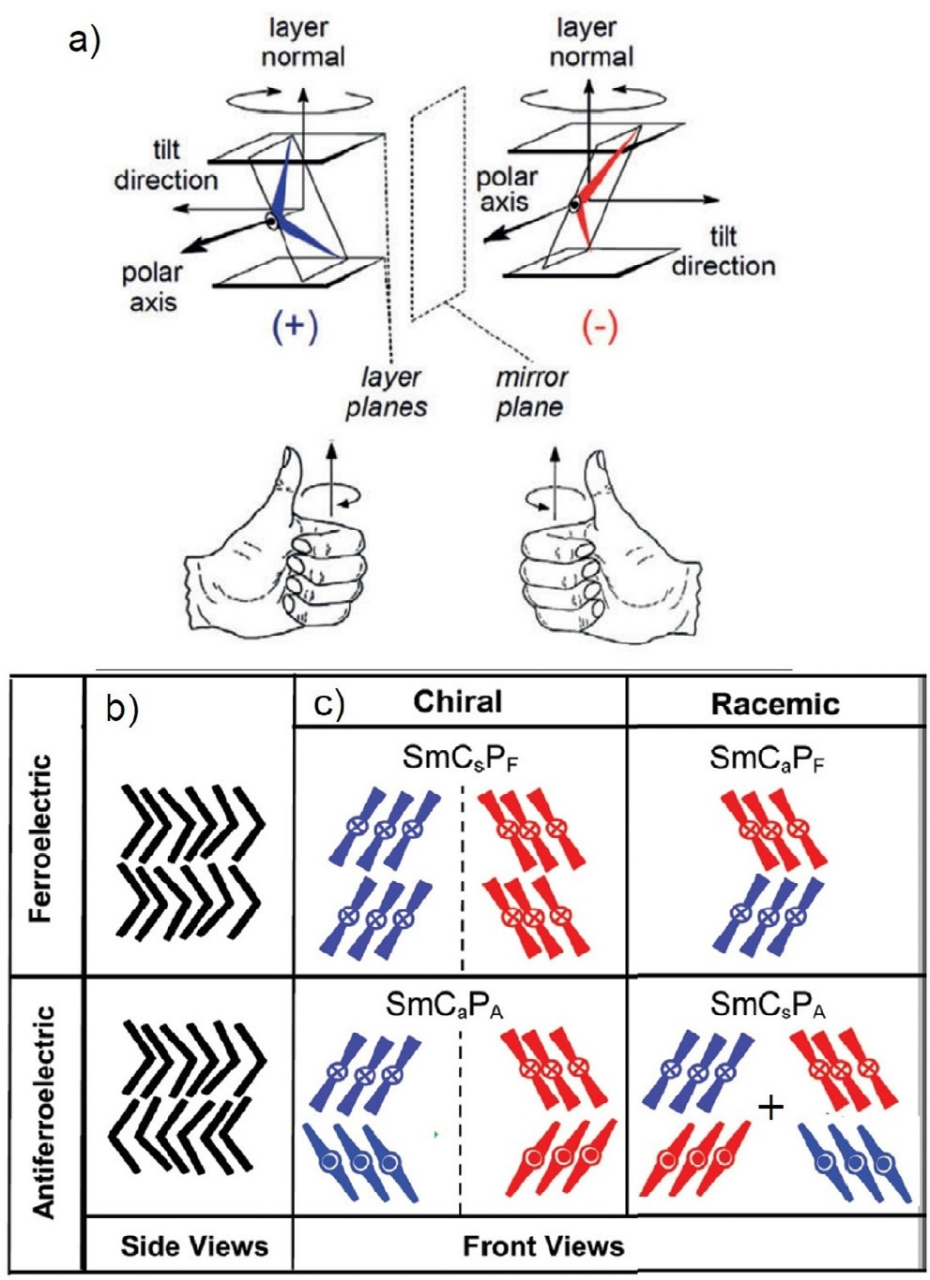
Layer chirality and the structures of the polar SmC phases of bent‐core mesogens. a) The orthogonal combination of tilt and polar order leads to reduced *C*_2*v*_ symmetry and superstructural chirality of the layers (reproduced with permission from ref. [25], copyright 2006, The Royal Society of Chemistry); blue/red color indicates the chirality sense. b, c) shows the four diastereomorphic structures of tilted polar smectic phases of bent‐core mesogens: b) side views, showing synpolar (“ferroelectric”: P_F_) and antipolar (P_A_) order in adjacent layers, and c) front views showing the tilt correlation (C_a_ and C_s_ refer to the anticlinic and synclinic tilt, respectively) of the bent‐core molecules in adjacent layers; here the polar direction is indicated by dots (pointing out from back to front) and crosses (pointing to the back); reproduced with permission from ref. 44, copyright 2019, The Royal Society of Chemistry.

The more surprising is the recent observation that similar short pitch heliconical phases can even be observed for achiral molecules.^9^, ^12^, ^19^, ^20^ The use of achiral compounds for helix generation reduces the cost of materials synthesis, because it is independent on the chiral pool, and more importantly, both senses of handedness are easily accessible in a process of mirror symmetry breaking and chirality amplification.^2^, ^3^, ^7^, ^9^, ^12^ Moreover, in soft matter the helical structures of these transiently chiral molecules can be switched by external stimuli either on‐off^21^, ^22^ or between the enantiomeric states.^23^, ^24^ The mirror symmetry breaking in systems formed by bent molecules received special interest^25^, ^26^, ^27^, ^28^, ^29^, ^30^ because it is associated with the development of polar order and there are different sources of superstructural chirality.^12^, ^30^, ^39^ One is due to the tilted organization of the molecules with uniform polar direction parallel to the layer planes, providing a chiral *C*_2*v*_ symmetry of the layers (layer chirality, Figure 2 a).^26^ The combination of tilt direction and polar direction determines the chirality sense of the layers which is inverted either by reversing tilt direction or polar direction and retained by the simultaneous inversion of both. As shown in Figure 2 b, c, the chiral layers can pack in stacks with uniform or opposite tilt direction (s=synclinic or a=anticlinic) and polar direction (F=ferroelectric=synpolar or A=antiferroelectric=antipolar) thus leading to four stereoisomeric superstructures (see Figure 2 b, c). The SmC_s_P_F_ and SmC_a_P_A_ phases are homogeneously chiral because they are formed by layers with uniform chirality sense (either blue or red). The other two, SmC_s_P_A_ and SmC_a_P_F_, are formed by alternating layers with opposite chirality (mixed blue and red) and therefore are achiral (racemic).^26^ Another source of chirality is provided by the significant transient chirality of energy minimum helical molecular conformations of the bent‐core molecules (intramolecular twist).^12^, ^31^, ^32^ The synchronization of the transient molecular helicity of adjacent molecules supports mirror symmetry breaking.^12^ In the cases of bent‐core self assembly, known so far, the intermolecular twist occurs mainly transversal between the molecular long axes (Figure 1 b) and the helix axis develops along the layers, leading to layer distortion. Rigid layers break into helical nano‐ribbons (helical nano‐filament phases, HNF, B4 phases, Figure 1 f)^33^, ^34^, ^35^, ^36^, ^37^, ^38^ and soft layers form more disordered sponge‐like structures, the so‐called dark conglomerate phases (DC‐phases, Figure 1 g).^12^, ^25^, ^30^, ^39^, ^40^, ^41^, ^42^, ^43^, ^44^

**Scheme 1:**
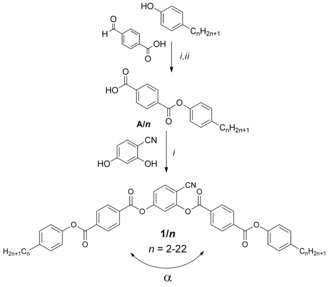
Synthesis of compounds **1*/n***; *Reagents and conditions*: i: 1. 4‐substituted benzoic acid, SOCl_2_, 80 °C, 2 hrs, 2. phenol, DCM, pyridine, 50 °C, 2 hrs; ii: NaClO_3_, KH_2_PO_4_, resorcinol, *t*BuOH, 20 °C, 1 h.

Herein we focus on helicity occurring along the layer normal (longitudinal twist between the short, secondary, molecular axes, Figure 1 a) and its effects on the LC self‐assembly of bent molecules. The investigated compounds **1/*n*** (Scheme 1) are based on the 140° bent 4‐cyanoresorcinol core (*α*=140°) with a reduced molecular bent compared to the 120° angle of ordinary bent‐core molecules.^45^, ^46^, ^47^, ^48^, ^49^, ^50^, ^51^ In these molecules the 4‐cyanoresorcinol core is connected with two electron deficit terephthalate wings, providing a dense packing.^52^, ^53^, ^54^, ^55^, ^56^ A summary of previous work on selected individual members of the 4‐cyanoresorcinol based LCs and the development of the distinct models of their phase structures is given in reference 57 as well as in Section S4 and Table S2 in the Supporting Information. Compounds **1/*n*** with *n=*14 and 16 are of special interest, because for **1/16** the existence of a heliconical smectic LC phase was first observed by optical investigations^19^, ^58^, ^59^, ^60^, ^61^ and AFM,^19^ and an incommensurate 3‐layer structure was corroborated for **1/14** by soft resonant X‐ray scattering at the carbon K‐edge (RSoXR).^20^ Herein we report the complete homologous series of compounds **1/*n*** with even numbered *n* ranging from *n=*2 to 22 to provide a fundamental understanding of spontaneous mirror symmetry breaking by helix formation and its effect on the phase sequence and the phase structure in the context of diasteromeric relations between layer chirality^26^ and helix chirality. It is shown that helix formation requires a weak layer coupling as found at the transition from non‐tilted (SmA) to tilted (SmC) and from anticlinic (SmC_a_) to synclinic tilted (SmC_s_) smectic phases. Moreover, this transition has to be coupled with the paraelectric‐(anti)ferroelectric transition, leading to a polar smectic phase with uniform layer chirality. This work also answers the question, why in some cases the heliconical phase represents a ground state structure, whereas in others it is only field induced. The relations with other heliconical structures, as for example found in the twist‐bent nematic phases (N_TB_, see Figure 1 d)^62^, ^63^, ^64^, ^65^, ^66^ and the related smectic phases (SmC_TB_) of bent mesogenic dimers,^67^ and especially the heliconical smectic phases of permanently chiral rod‐like mesogens (SmC_α_*, SmC_FI_* phases)^17^ will be discussed briefly. With lowering temperature the helix is removed^57^ and it is shown that under these conditions a non‐tilted and ferroelectric switching SmA′P_F_ phase represents an alternative non‐chiral way for the transition from anticlinic to synclinic tilt.

## Experimental Section

**Synthesis**: The synthesis of the compounds was conducted as shown in Scheme 1 by acylation of 4‐cyanoresorcinol with the properly substituted benzoyl chlorides in CH_2_Cl_2_ in the presence of pyridine.^19^ The synthetic procedures and analytical data of the new compounds are collated in the Supporting Information, together with the structural analysis data. The synthesis and analytical data of compounds **1/6**, **1/12**, **1/16** and **1/18** have been reported in previous work.^19^, ^55^

**Methods**: The investigation of the synthesized compounds was performed by polarizing optical microscopy (POM), differential scanning calorimetry (DSC), X‐ray diffraction (XRD) and by switching experiments, electro‐optical and dielectric investigations as described in the Methods section in the Supporting Information.

## Results and Discussion

**Figure 3:**
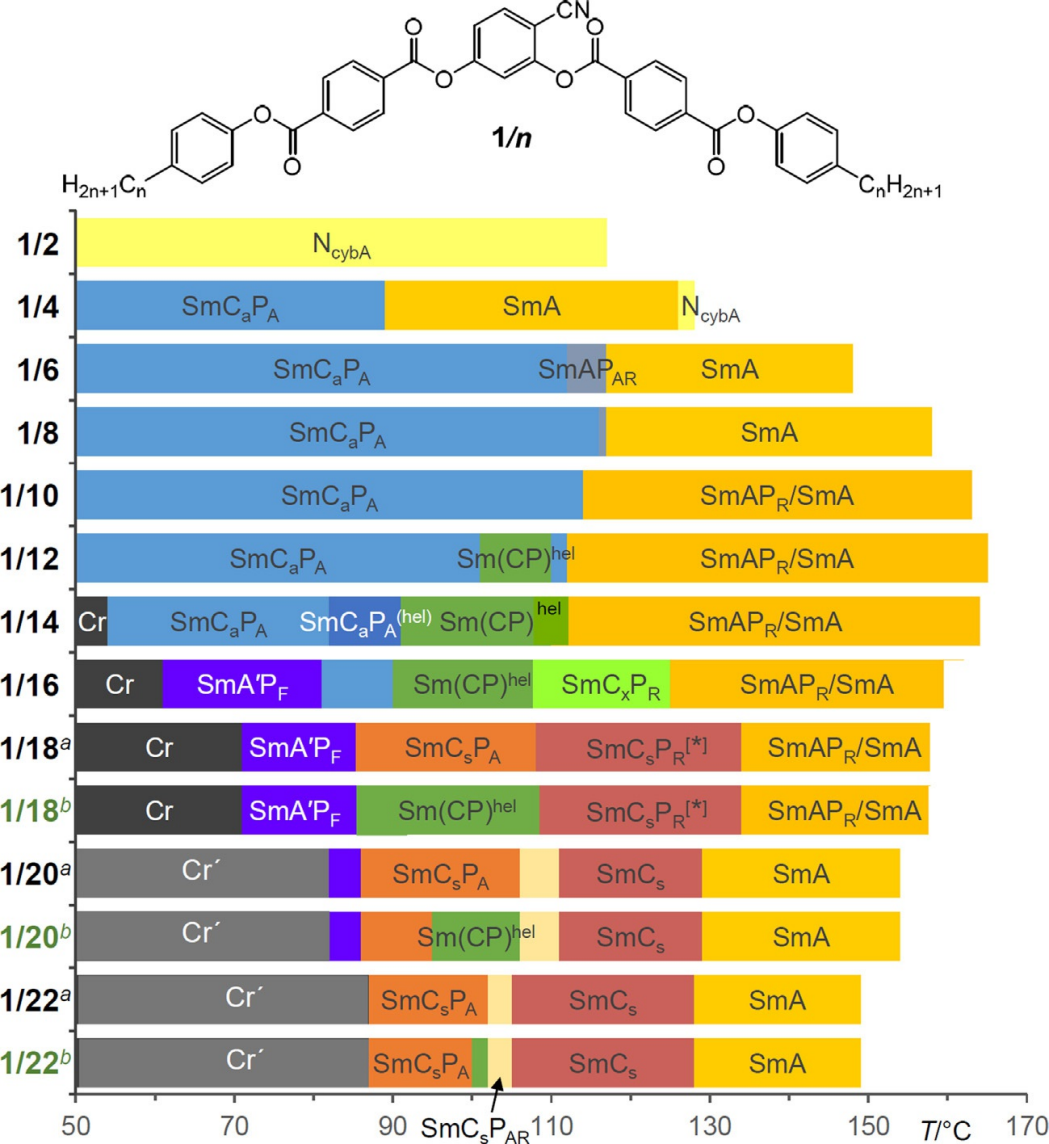
Bar diagram of compounds **1*/n*** showing the development of the LC phases on cooling and depending on chain length; for **1/18–1/22** the phase structures depend on pre‐treatment; [a] shows the phase transitions before applying an external electric field and [b] after application of a few cycles of an AC field (see Table 1 for abbreviations and Table S1 for numerical values).

The LC phases, transition temperatures and transition enthalpy values of all compounds, as recorded on heating, are collated in Table 1, whereas Figure 3 shows graphically the phase sequences on cooling (see Table S1 for the corresponding numerical values).

**Table 1:** Phase transitions of compounds **1*/n*** depending on chain length, measured upon heating.^[a]^

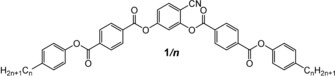	
**1/*n***	*T*/°C [Δ*H*/kJ mol^−1^]
**1/2**	Cr 117 [32.2] (N_cybA_ 117 [0.3]) Iso
**1/4**	Cr 153 [53.0] (SmC_a_P_A_ 90 [1.5] SmA 126 [1.7] N_CybA_ 129 [0.8]) Iso
**1/6** 55	Cr 128 [39.6] (SmC_a_P_A_ 113 [1.1] SmAP_AR_ ≈118 [–]) SmA 150 [5.3] Iso
**1/8**	Cr 125 [43.9] (SmC_a_P_A_ 116 [0.6] SmAP_AR_ ≈118 [–]) SmA 161 [6.8] Iso
**1/10**	Cr 112 [38.2] SmC_a_P_A_ 114 [0.5]) SmAP_R_/ SmA 165 [7.8] Iso
**1/12** 55	Cr 114 [42.6] (SmC_a_P_A_ 101 [–] Sm(CP)^hel^ 112 SmC_a_P_A_ 113 [0.8]) SmAP_R_/SmA^[b]^ 167 [8.0] Iso
**1/14**	Cr 113 [48.3] (SmC_a_P_A_^[c]^ 83 [–] SmC_a_P_A_^(hel)^ 99 [–] Sm(CP)^hel^ 112 [1.0]) SmAP_R_/SmA 165 [8.0] Iso
**1/16** 19	Cr 110 [47.9] (SmA′P_F_^[d]^ 83 [–] SmC_a_P_A_ 92 [–] Sm(CP)^hel^ 110 [0.8]) SmC_x_P_R_ 127 [–] SmAP_R_/SmA 162 [7.7] Iso
**1/18** 19	Cr 114 [60.8] (SmA′P_F_^[d]^ 85 [–] SmC_s_P_A_ 110 [1.1]) SmC_s_P_R_^[^*^]^ 135 [–] SmAP_R_/SmA 161 [7.4] Iso After field treatment:^[e]^ Cr 71 SmA′P_F_ 85 Sm(CP)^hel^ 110 SmC_s_P_R_^[^*^]^ 135 SmAP_R_/SmA 160 Iso
**1/20**	Cr 106 [79.5] (SmA′P_F_^[d]^ 87 [–]) SmC_s_P_A_ 108 [1.2] SmC_s_P_AR_ 113 [–] SmC_s_ 130 [–] SmA 156 [6.5] Iso After field treatment:^[e]^ Cr′ 82 SmA′P_F_ 86 SmC_s_P_A_ 95 Sm(CP)^hel^ 106 SmC_s_P_AR_ 111 SmC_s_ 129 SmA 155 Iso
**1/22**	Cr 108 [91.7] (SmC_s_P_A_ 105 [1.3] SmC_s_P_AR_ 108 [–]) SmC_s_ 130 [–] SmA 153 [5.8] Iso After field treatment:^[e]^ Cr′ 87 SmC_s_P_A_ 100 Sm(CP)^hel^ 102 SmC_s_P_AR_ 106 SmC_s_ 128 SmA 152 Iso

[a] Phase transitions on heating, for data on cooling, see Figure 3 and Table S1. The references refer to the first reported synthesis of the compounds, for a complete list of references, see Table S2. All transition temperatures with enthalpies represent peak maxima in the DSC traces, recorded on heating with a rate of 10 K min^−1^; transitions without enthalpies were determined under similar conditions by optical or electro‐optical methods in thin cells. Values in parenthesis refer to monotropic transitions, observed in second heating scans if crystallization could be suppressed. There could be a slight deviation of the DSC values given in Tables 1 and S1 from those observed by other investigations, usually performed in thin films (1–2 K, larger deviations can be found for the crystallization temperatures). Abbreviations: Cr, Cr′=crystalline solids (Cr′ indicates a crystalline phase, appearing with the same fan‐like optical texture as SmA and Sm(CP)^hel^ phases, see Figures S51, S53); Iso=isotropic liquid, N_cybA_=cybotactic nematic phase; SmA=de Vries type uniaxial lamellar phase; SmAP_R_=high permittivity paraelectric range of the SmA phase showing a single broad polarization peak per half period of an applied triangular E‐field; SmAP_AR_=high permittivity paraelectric SmA range showing two broad polarization peaks; SmA′P_F_=non‐tilted and ferroelectric switching lamellar phase; SmC_a_P_A_=anticlinic tilted and antiferroelectric switching lamellar phase; SmC_a_P_A_^(hel)^=SmC_a_P_A_ phase with short range helical structure; SmC_s_P_A_=synclinic tilted and antiferroelectric switching lamellar phase; Sm(CP)^hel^=heliconical lamellar phase; SmC_s_=synclinic tilted paraelectric lamellar phase; SmC_s_P_R_^[^*^]^=high permittivity paraelectric SmC_s_ phase showing a single broad polarization peak and domains with opposite optical rotation in homeotropic alignment (^[^*^]^); SmC_x_P_R_=high permittivity paraelectric SmC phase showing a single broad polarization peak; depending on the conditions the extinctions in the planar texture are either parallel or inclined to the direction of the polarizers. [b] There is no transition enthalpy for the SmA‐SmAP_R_ transition as mistakenly stated in ref. 55, the corrected DSC is shown in Figure 6 b. [c] This phase was designated as SmC_a_P_F_ in ref. 20, the reasons for using SmC_a_P_A_ are given in the section describing this helix‐free low temperature SmC_a_P_A_ phase. [d] This phase was designated as SmAP_A_ in ref. 57, the reasons for using SmA′P_F_ are given in the section describing this phase. [e] Phase transitions were determined by optical observations (crystallization temperatures were observed on cooling).

### Nematic and uniaxial smectic phases of the short chain compounds 1/2‐1/6

**Figure 4:**
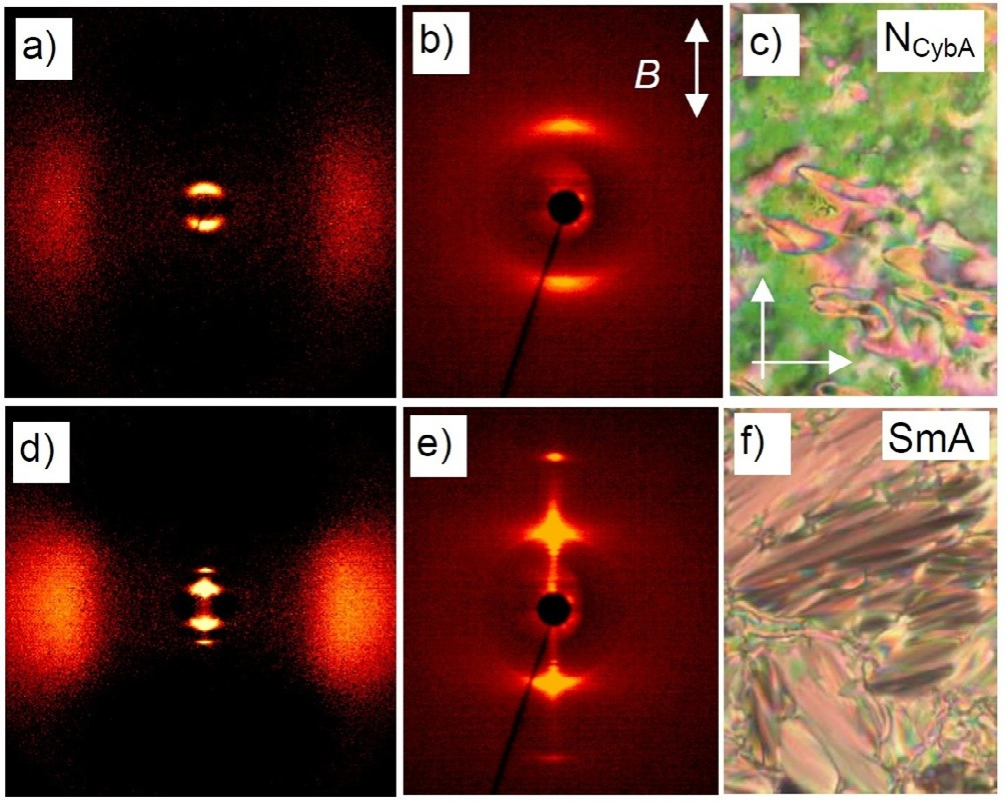
2D‐XRD patterns of a magnetically aligned sample and textures of compound **1/4**, a–c) in the nematic phase at *T=*133 °C (the double headed arrow indicates the direction of the magnetic field) and d–f) in the SmA phase at *T=*125 °C; a, d) wide angle scans, b, e) SAXS patterns and c, f) textures as observed between crossed polarizers; the arrows in c) indicate the directions of polarizer and analyzer.

**Figure 5:**
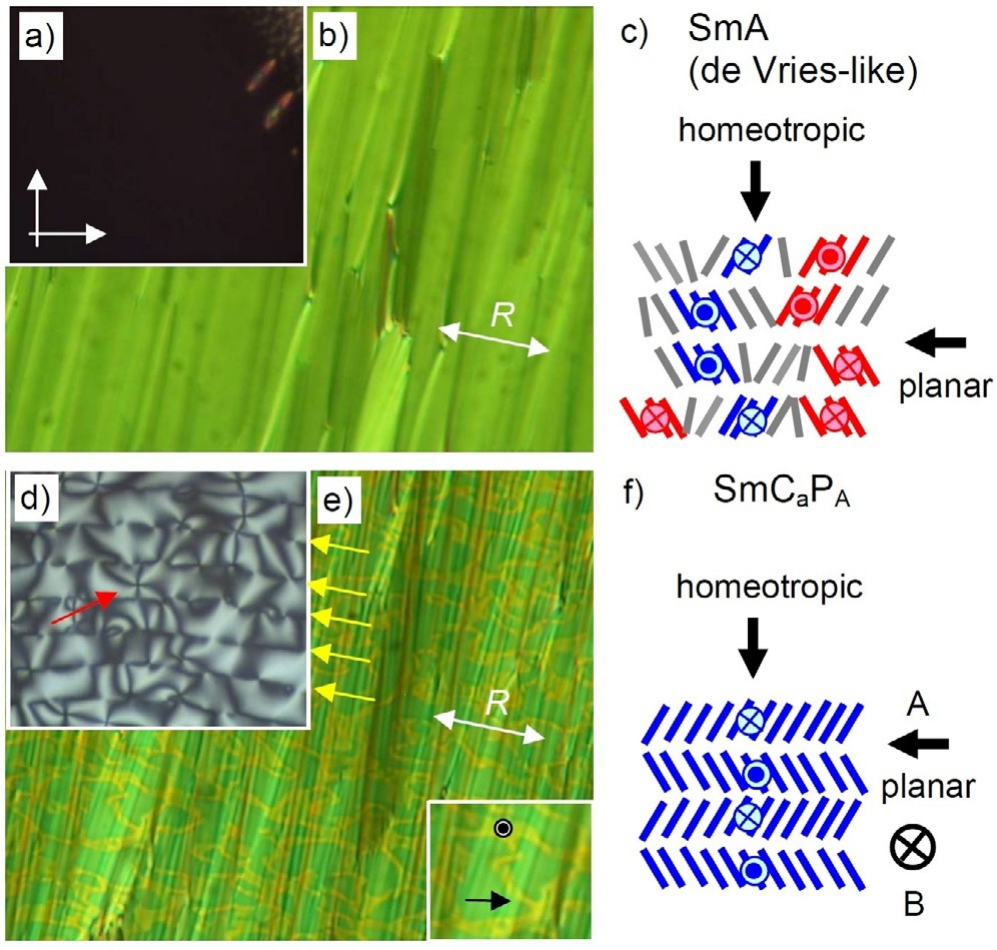
Optical investigation of compound **1/4** a, b) in the SmA phase at *T=*118 °C and d,e) in the SmC_a_P_A_ phase at *T=*80 °C. a, d) show the homeotropic textures between plane microscopic glass plates and b, e) the planar textures (PI coated ITO cell 6 μm, *R* is the rubbing direction of the substrates); b) show the smooth texture in the SmA phase and e) the “speckled” texture in the SmC_a_P_A_ phase; the inset in e) shows the polar directions in the distinct domains; the width of the images is 0.6 mm; c, f) show the models of the molecular organization in the two phases (the bent molecules are simplified and shown as rods; dots and crosses indicate the polar direction, corresponding to the bow direction, pointing out of and into the plane of projection, respectively; molecules in gray are tilted out of the plane and their polar direction is arbitrary); c, f) show the directions of view in homeotropic (perpendicular to the layer planes) and planar alignment (parallel to the layers); f) in the speckled texture the view in planar samples is along B in the green speckles and along direction A in the yellow continuum (for related textures of compounds **1/6** and **1/8**, see Figs. S1, S2, S7 and S11).

Only a nematic phase (N) was observed for the shortest compound **1/2**. From the XRD patterns of magnetically aligned samples it can be deduced that this nematic phase is of the cybotactic type, characterized by a relatively high intensity of the diffuse small‐angle scattering. This scattering is perpendicular to the maxima of the diffuse wide angle scattering, indicating an on average non‐tilted organization of the molecules in the cybotactic clusters (N_CybA_, see Figure 4 a, b for the diffraction patterns of **1/4**). This type of non‐tilted cybotactic nematic phases is rare for bent‐core mesogens^68^ which usually form the skewed cybotactic nematic phase (N_CybC_).^51^, ^68^ For **1/4** this N_CybA_ phase is observed as a high temperature phase only in a small temperature range above a uniaxial smectic phase (SmA) and it is removed for **1/6** and all following homologues. Upon cooling, at 126 °C, the diffuse small angle scattering of **1/4** condenses into a sharp Bragg peak (*d=*3.4 nm; *L*_mol_=3.9 nm, *d*/*L*_mol_=0.87) with its second order on the meridian (Figure 4 d, e) and perpendicular to the maxima of the diffuse wide angle scattering on the equator. This indicates the transition to an orthogonal smectic phase (SmA). In optical investigations the typical fan texture with extinction brushes parallel to the polarizers in planar alignment and the isotropic appearance in homeotropic alignment confirm the SmA phase (see Figure 5 a, b). The SmA phase is retained for all investigated compounds **1/*n*** and starting with **1/6** it is formed directly from the isotropic liquid phase on cooling.

### Transition from randomized to anticlinic tilt in the smectic phases of compounds 1/4‐1/10

On further cooling compound **1/4**, at *T=*90 °C, an additional phase transition is observed, associated with a small peak in the thermograms (Δ*H*=1.5 kJ mol^−1^, Table 1). Optical investigation of homeotropically aligned samples (layer planes organized parallel to the substrate surfaces) between crossed polarizers show that at this phase transition the isotropic appearance of the homeotropic SmA phase is replaced by a gray schlieren texture as typical for the transition to a biaxial smectic phase (Figure 5 a–d). The developing periodic stripe pattern (yellow arrows in Figure 5 d) is typical for biaxial SmA_b_ phases as well as for anticlinic tilted SmC_a_ phases.^69^, ^70^, ^71^ The presence of 4‐brush disclinations (red arrow) between the stripes is in favour for an anticlinic SmC_a_ structure of this biaxial smectic phase; in a non‐tilted biaxial SmA_b_ phase only two‐brush disclinations would be expected.^69^ In planar alignment, where the layers are organized perpendicular to the substrate surfaces, the dark extinctions retain their positions parallel to the directions of polarizer and analyzer, confirming the absence of a uniform (synclinic) tilt in the low temperature smectic phase (Figures S1, S2 and S7). Associated with this phase transition a speckled texture develops (Figure 5 b→e) which is unique for the anticlinic SmC_a_ phases (SmC_a_P_A_) of the compounds under investigation. It is assumed to be due to two different modes of planar alignments of the bent molecules, either with the bow plane parallel (low Δ*n*, green) or perpendicular to the substrate surfaces (high Δ*n*, yellow), see Figure 5 f, views B and A, respectively.^20^

**Figure 6:**
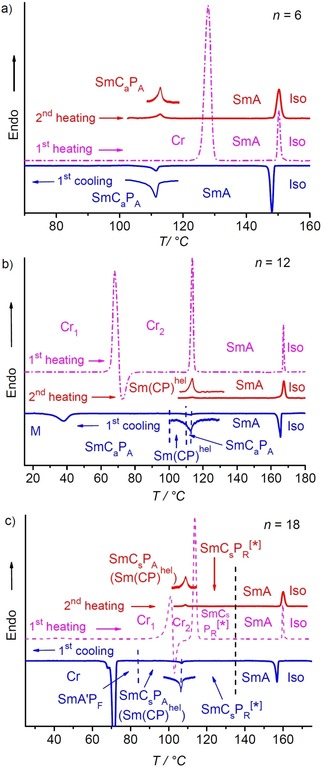
Representative DSC traces (10 K min^−1^) of compounds a) **1/6** b) **1/12** and c) **1/18** recorded on first heating (dashed magenta line) following cooling (blue) and on second heating after cooling to 100 °C (red). The insets show enlargements of the paraelectric‐(anti)ferroelectric transition peak in second heating and on cooling (for other DSCs, see Figures S6, S10, S22, S26, S43 and S52).

**Figure 7:**
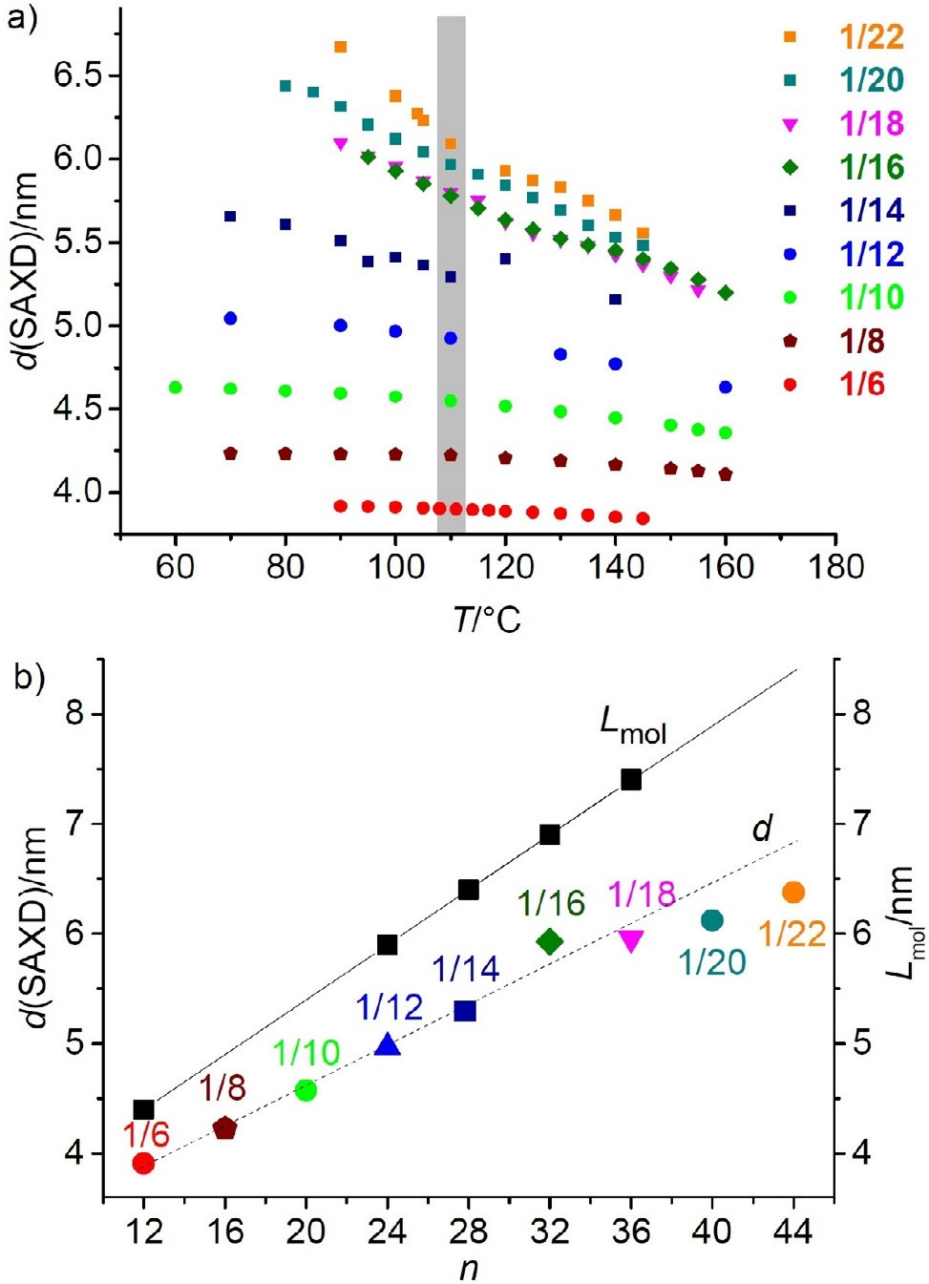
a) Dependence of the layer distance *d* on the chain length and temperature; the gray bar indicates the temperature range of the paraelectric‐(anti)ferroelectric transition; b) *d*‐values measured at 100 °C and molecular length (*L*_mol_, determined with CPK space filling molecular models in the most stretched conformation with 140° bending angle and all‐*trans* conformation of the alkyl chains) of compounds **1*/n***; the values of **1/14** were taken from ref. [20]; for individual plots of *d*‐values, see Figures S3, S8, S12, S16, S27, S33, S44 and S53.

This transition with small enthalpy is observed for all investigated compounds and it is the only transition in the whole LC range which is associated with an enthalpy change, and for all compounds **1/6**–**1/20** it takes place around 112±4 °C, as shown in Figure 6 for the DSC traces of compounds **1/6**, **1/12** and **1/18** as representative examples (see also Tables 1, S1 and Figure 3). For compounds **1/4** to **1/14** this transition coincides with the onset of phase biaxiality, whereas for all following compounds with longer chains phase biaxiality already develops before this transition. In the powder XRD patterns there is an almost continuous increase of the layer spacing with decreasing temperature, but neither the expected decrease of *d*, nor any step in the *d*‐values or change of the inclination of the *d*=*f*(*T*) curve takes place at this transition (Figure 7 a). The absence of a *d*‐value discontinuity at the transition from the SmA phase to the biaxial phase suggests that tilt is already present in the SmA phase, but it is randomized as typical for de Vries phases.^72^ The continuous increase of *d* with lowering temperature is attributed to a growing packing density of the molecules, leading to a stretching of the alkyl chains, compensating and exceeding the shrinkage of *d* due to the tilt.

The ratio *d*/*L*_mol_ is in the range between 0.85–0.89 for all compounds **1/4‐**‐**1/16** (Figure 7 b), which in principle would allow tilted as well as non‐tilted arrangements (see Section S4 in the Supporting Information). XRD of aligned samples also provides no clear indication of tilt (see Figures S4, S15 and S28). Only in few cases of compounds **1/12–1/16**, electro‐optical investigations uncover the actually tilted organization with an optical tilt (tilt of the aromatic cores) of 15–20°.^20^, ^57^, ^59^ For compounds **1/18–1/22** with longer chains the *d*/*L*_mol_ ratio becomes smaller (0.80, Figure 7 b), in line with an enhanced tendency to form synclinic tilted SmC_s_ phases (Table 1), though the tilt angle itself appears to be not increased. In the polar smectic phases of compounds **1/4–1/10** with short chains the switching takes place exclusively by rotation around the long axis and in this case a SmC_a_P_A_ phase cannot be distinguished from a non‐tilted SmAP_A_ phase by optical and electro‐optical investigations. However, the linear increase of *d* from **1/6** to **1/14** (Figure 7 b) suggests that the tilt should be almost identical in these smectic phases. The average birefringence of the planar sample does not significantly change at the de Vries to SmC_a_P_A_ transition (Figure 5 b–e), though an increase of birefringence is usually observed at the de Vries SmA to SmC_s_ transition of rod‐like molecules.72e In contrast, the de Vries SmA to anticlinic SmC_a_ transition is associated with a decrease of birefringence.^73^ The decrease of birefringence at this SmA‐SmC_a_P_A_ transition could be compensated by the increase of birefringence due to the developing polar order. In line with this, in the speckled texture below the phase transition around 112±4 °C there are areas with increased birefringence (yellow) due to the developing polar order (see below) as well as areas with slightly reduced birefringence (green) compared to the SmA texture (Figure 5 e), the latter associated with the emerging anticlinic tilt in the SmC_a_P_A_ phase.

### Development of polar order and the paraelectric‐(anti)ferroelectric transition

#### Electro‐optical investigations

**Figure 8:**
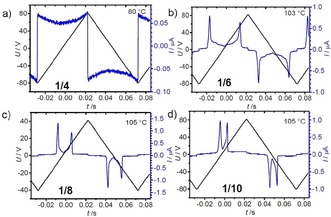
Polarization current response curves depending on alkyl chain length. a) **1/4**, b) **1/6**, c) **1/8** and d) **1/10** as measured in a 6 μm PI coated ITO cell at the indicated temperatures in the SmC_a_P_A_ range about 10 K below the paraelectric‐(anti)ferroelectric transition temperature under an applied peak‐to‐peak voltage of 160 V_pp_; for data at other temperatures, see Figures 9, S5, S9 and S14.

**Figure 9:**
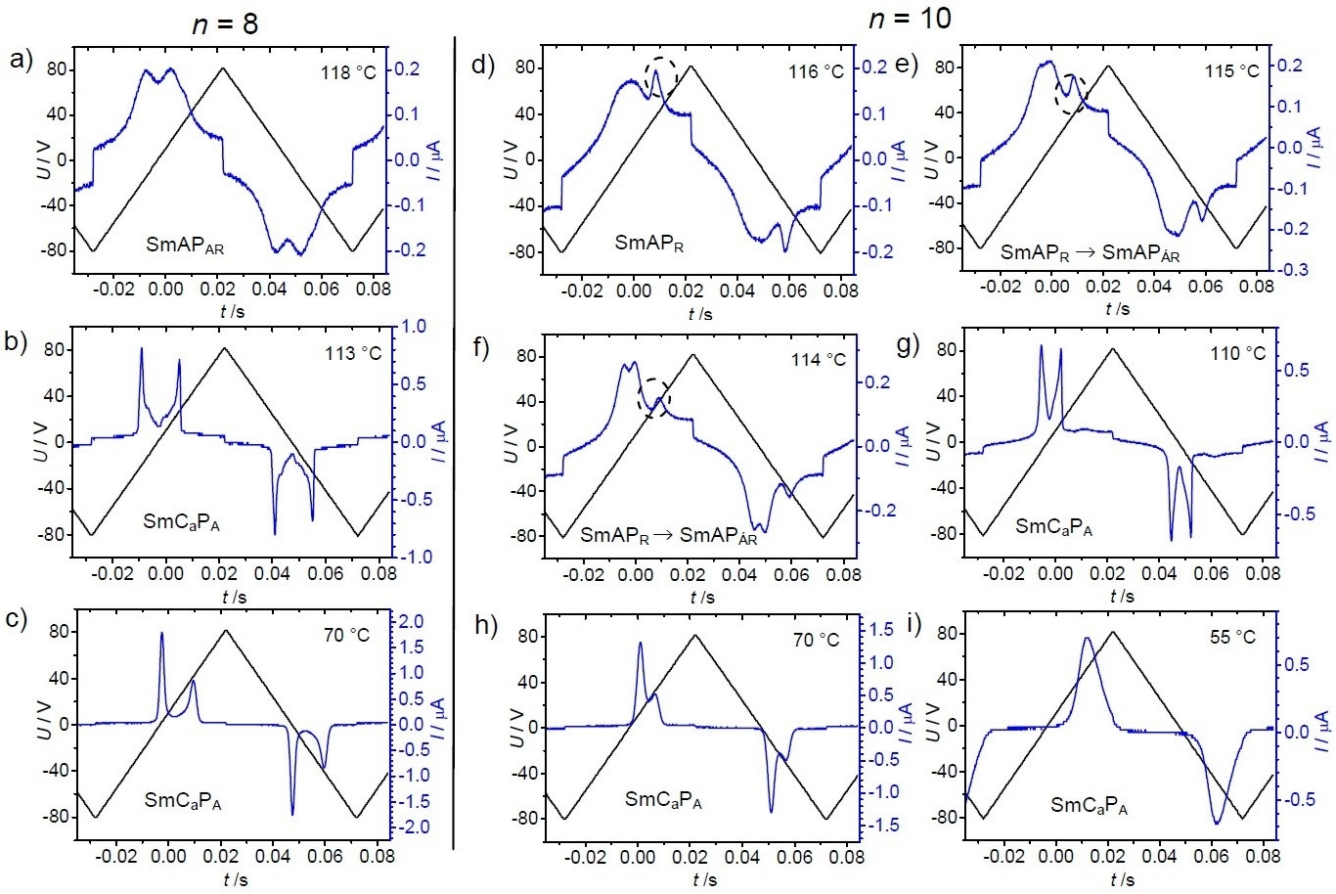
Distinct modes of development of the polarization current curves: a–c) via SmAP_AR_ for compound **1/8** and d–i) via SmAP_R_ for compound **1/10** (6 μm PI coated ITO cell, 160 Vpp, 10 Hz) measured with decreasing temperature, the encircled peak is due to conductivity; see Figure S9 and S14 for additional temperatures.

For the short chain compound **1/4** there is no response on an electric field in the SmA phase region, meaning that this SmA phase is considered as apolar. Also, in the biaxial smectic phase occurring below the transition peak at 90 °C no polarization peaks can be observed under a triangular wave AC field (Figure 8 a), even at an applied peak‐to‐peak voltage of 200 V_pp_ across a 6 μm cell. However, the shape of the polarization current curve shows that at higher voltage an antiferroelectric switching could be expected. For compound **1/6** two polarization current peaks could be observed already at a lower applied voltage of only 160 V_pp_ in the same 6 μm cell. Two widely separated and very broad peaks appear at 118 °C in the SmA range close to the DSC transition peak which become sharp at the phase transition at *T=*113 °C (see Figures 8 b and S5). This means that in this SmA phase polar cluster (ferroelectric grains) with antipolar correlation grow under the applied field, leading to a high permittivity paraelectric SmAP_AR_ range^74^ upon approaching the uniaxial‐biaxial transition (Figure 9 a).^48^, ^49^, ^53^ Below the phase transition two sharp polarization peaks indicate antiferroelectric switching which reaches polarization values of about 500–700 nC cm^−2^. Considering the anticlinic tilted organization of the molecules this phase is designated as SmC_a_P_A_. The two polarization current peaks come closer by alkyl chain elongation (Figure 8 c, d), indicating an increasing correlation length of polar order with growing chain length.

**Figure 10:**
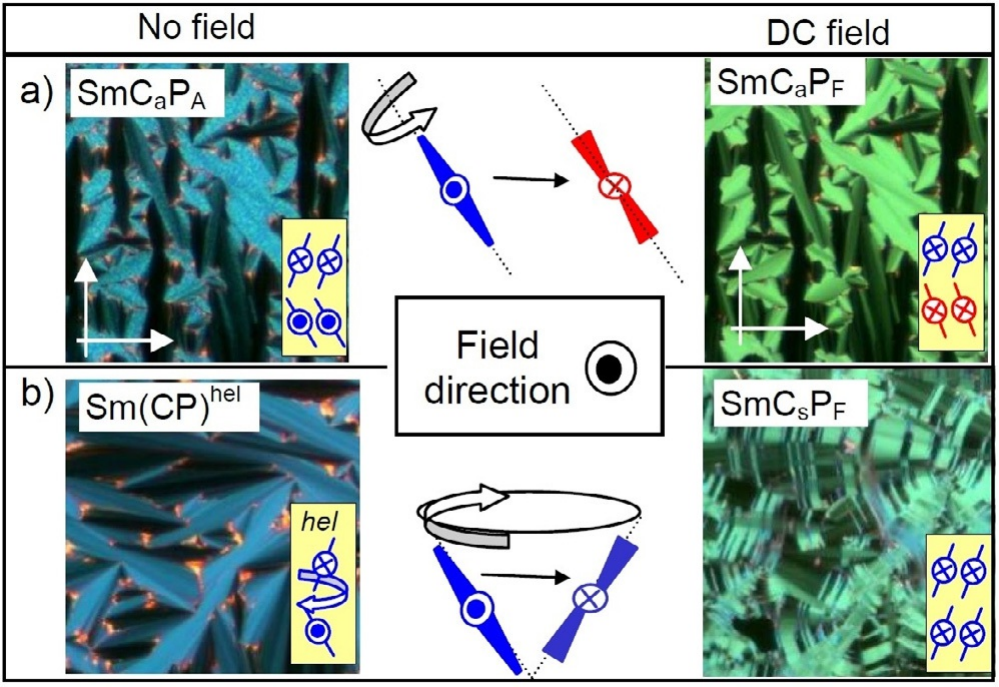
a) Switching in the helix‐free SmC_a_P_A_ phase by rotation around the long axis reverses the polar direction (dot vs. cross) and chirality in every second layer (indicated by red/blue color), the tilt direction does not change and therefore the position of the extinctions in the textures is retained (**1/10** at 100 °C); b) In the Sm(CP)^hel^ phase switching takes place by precession on a cone which reverses the polar direction and the tilt direction simultaneously, while the sign of layer chirality is retained; this switching is associated with a rotation of the optical axis (optical switching), often associated with tilt domain formation (**1/14** at 110 °C).

In the SmA phases of compounds **1/10**–**1/14** only one broad single polarization peak develops upon approaching the paraelectric‐(anti)ferroelectric phase transition on cooling (see Figure 9 d). This single peak indicates a Langevin switching of ferroelectric grains with SmC_s_P_F_ structure developing a synpolar correlation under an applied E‐field (SmAP_R_ range^53^, ^75^). As shown in Figure 9 d→e→f for compound **1/10**, it splits into two broad peaks just at the uniaxial‐biaxial transition, and on further cooling the peaks become sharper in the macroscopic polar SmC_a_P_A_ phase (Figure 9 g). Hence, with alkyl chain elongation the high permittivity paraelectric phase changes from SmAP_AR_ to SmAP_R_ (Figure 3, Table 1). For all compounds **1/4**– **1/10** the switching in the SmC_a_P_A_ phase takes place by rotation around the long axis (SmC_a_P_A_↔SmC_a_P_F_) under all conditions, that is, the switching can only be detected by a change of the birefringence, whereas the orientation of the dark extinctions, and thus the orientation of the optical axis does not change (Figures 10 a and S13).^76^ This mode of switching is preferred due to the easy rotation around the long axis of compounds **1/*n*** with a weakly bent 4‐cyanoresorcinol core.

#### Dielectric investigation

**Figure 11:**
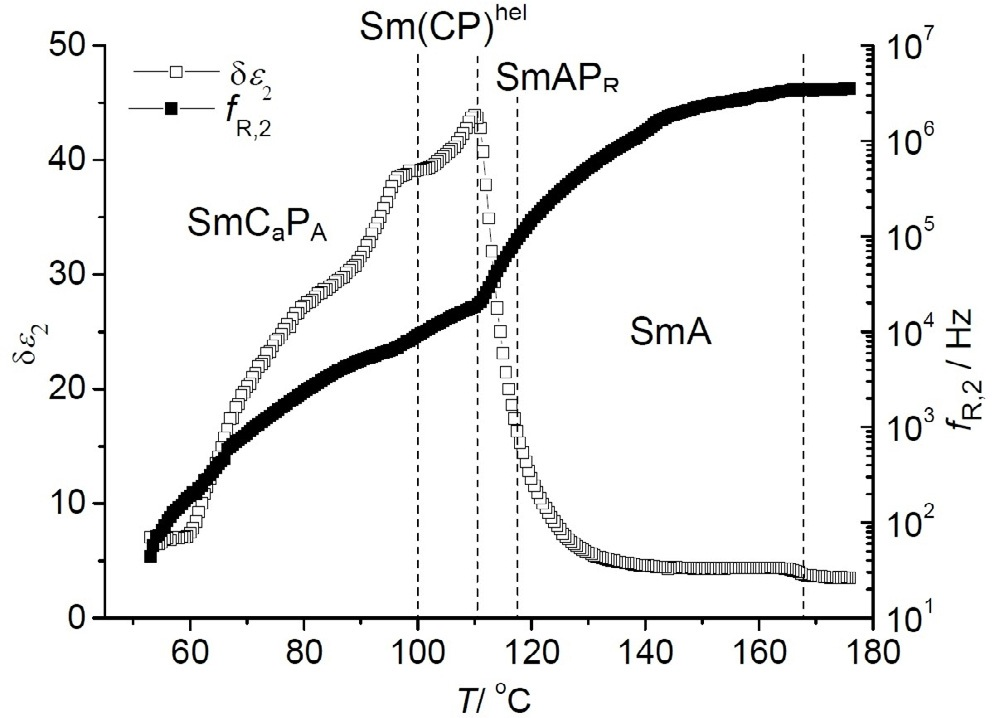
The plot of dielectric relaxation strength, δ*ϵ*_2_, and relaxation frequency, *f*_R,2_, of compound **1/12**, for the mid frequency process, P2, as a function of temperature.

Dielectric spectroscopy of **1/12**, as example, was performed in the frequency range between 1 Hz and 10 MHz and three relaxation processes, P1, P2 and P3 were observed in the measured frequency range (for details, see Figure S20 and related discussions in the Supporting Information). The medium frequency relaxation process P2, exists in the measured frequency range in all LC phases and was assigned to the polar switching mechanism. Figure 11 shows the temperature dependence of dielectric strength and relaxation frequency for P2. As the sample was cooled down from the isotropic phase, initially, δ*ϵ*_2_ does not change significantly whereas *f*_R,2_ continuously decreases. When the sample approaches 120 °C, a massive increase in δ*ϵ*_2_, changing from ≈5 to 45 and a significant decrease in *f*_R,2_ (from 3×10^6^ to 2×10^4^ Hz) were observed. The increase of δ*ϵ*_2_ confirms ferroelectric grains with growing polarization. Below *T=*112 °C, in the polar phase range, δ*ϵ*_2_ decreases, still remaining high in magnitude, with a smooth step like change in both δ*ϵ*_2_ and *f*_R,2_ at the Sm(CP)^hel^ to SmC_a_P_A_ phase transition (see next section). Further, δ*ϵ*_2_ continuously decreases until crystallization at 60 °C. This decrease is due to the significant growth of the ferroelectric grains, fusing to almost infinite layers in the polar smectic phases. Thus the phase transition around 112–113 °C can be attributed to the paraelectric‐(anti)ferroelectric transition with Curie–Weiss‐type divergence (see Figure 11).^53^, ^77^

### Helix formation in the smectic phases of the medium chain length compounds 1/12–1/16—the heliconical smectic phase Sm(CP)^hel^

**Figure 12:**
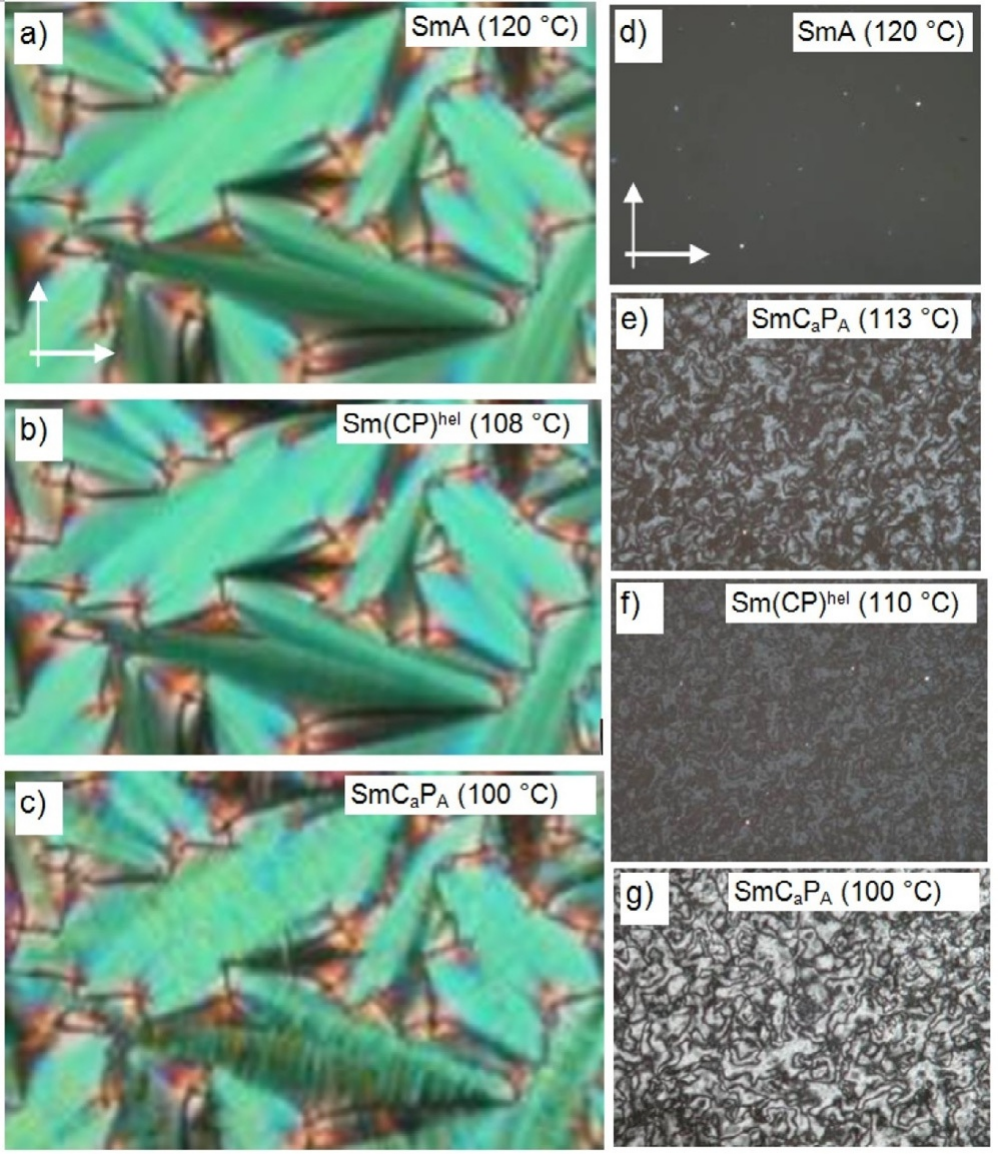
Textural changes observed at the SmA→Sm(CP)^*hel*^→SmC_a_P_A_ transitions of **1/12**; a–c) in a planar cell (6 μm PI‐coated ITO cell) and d‐g) in homeotropic alignment (ordinary, non‐treated microscopy glass plates) at the indicated temperatures; the width of the images is 0.6 mm (for textures of **1/14** and **1/16**, see Figures S23–S25 and S29 and S30).

For compounds **1/12‐**‐**1/16** additional uniaxial and polar smectic phases were observed below the paraelectric‐(anti)ferroelectric transition. Similar to the shorter homologues, on cooling in homeotropic samples of **1/12** a birefringent schlieren texture is formed at the onset of the transition from SmAP_R_ to SmC_a_P_A_ at 113 °C (Figure 12 d→e). However, the birefringence significantly decreases immediately after the phase transition and the homeotropically aligned phase becomes almost isotropic at the end‐set of the phase transition at *T=*111 °C (Figure 12 f). The birefringence increases again on further cooling below 101 °C (Figure 12 g), but without any indication of an additional phase transition in the DSC traces (Figure 6 b). For compounds **1/14** and **1/16** the homeotropic samples become even completely dark at the end‐set of this DSC peak, indicating the transition to a uniaxial LC phase (Figures S23 and S29). For these compounds the small birefringent range around the phase transition can only be observed upon fast cooling (>10 K min^−1^) whereas upon slower cooling or upon heating no intermediate birefringent state is observed. On further cooling the birefringence of the homeotropic sample emerges again at 91 °C and 90 °C, respectively (see Table S1 and Figures 3, S23 and S29). This change is also not associated with a DSC peak. In planar samples the smooth SmA‐like fan texture with extinctions parallel to the polarizers is observed at all temperatures; there is only a faint, almost invisible, stripe formation on the fans at the paraelectric‐(anti)ferroelectric phase transition (Figure 12 a→b) and a clearly visible strip pattern across the fans develops at the transition to the biaxial phase (Figure 12 b→c), which then changes to the speckled texture as typical for the SmC_a_P_A_ phase.

**Figure 13:**
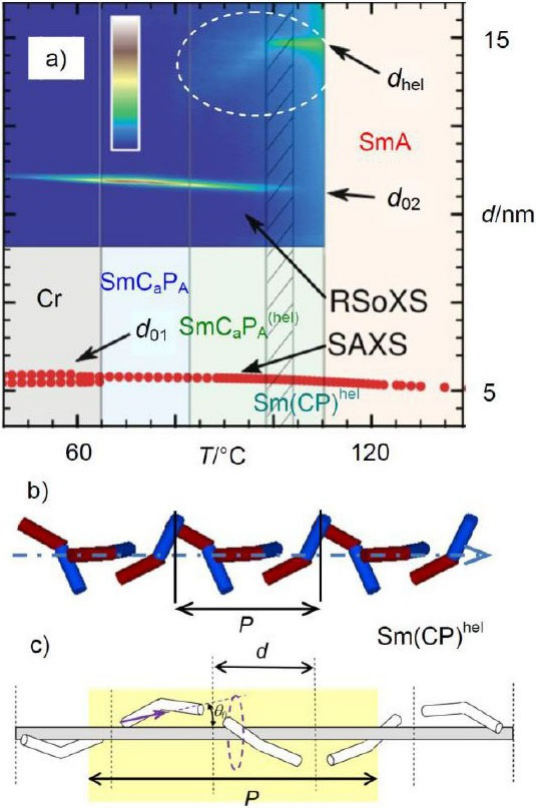
The Sm(CP)^hel^ phase. a) Development of the XRD data (SAXS and RSoXS) in the LC phases of **1/14** depending on temperature; b) shows a computer generated model of the helical structure with twist of 110 and 140° bent‐core angle^61^ and c) shows a simplified models; the helical pitch *P* in terms of a number of smectic layers is incommensurate and smaller than 3 times the thickness of a layer; a, b) were reprinted with permission from refs. 20 and 61, respectively, copyright (2019) by the American Physical Society (https://doi.org/10.1103/PhysRevLett.122.107801; https://doi.org/10.1103/PhysRevMaterials.3.045603).

Uniaxiality of a tilted smectic phase requires that either the tilt is randomized as in the de Vries SmA phases,^72^, ^73^, ^78^ or that a short pitch helical superstructure with a helix axis parallel to the layer normal is formed.^14^, ^78^ A helical structure was first proposed for the polar and uniaxial smectic phase of compound **1/14**.^58^ It was observed that an in‐plane electric field, applied to a homeotropic aligned sample at first induces a birefringence, but at a certain voltage a uniaxial state is formed.^58^, ^79^, ^80^ This was interpreted as a deformation of a helical phase, designated as SmAP_α_.^58^ The uniaxial state was assumed to result from helix deformation into a four layer structure with 90° twist between the secondary optical axes in adjacent layers.^58^ Later, as the tilted organization was recognized (see Section S4 in the Supporting Information), it was renamed to SmCP_α,_ 52 Sm(CP)_α_^[20]^ and SmC_s_P_F_^hel [19]^ and is herein designated as Sm(CP)^hel^. Helix formation was further confirmed for compound **1/16** by the observation of the so‐called deformed helical ferroelectric (DHF) effect in planar cells, indicating a helix deformation under an electric field^19^ similar to that previously known for helical SmC* and SmC_α_* phases of permanently chiral molecules.^81^ In addition, the helical superstructure with a pitch of only 14 nm was visualized by AFM of freeze fractured samples^19^ and finally unambiguously proven by RSoXS (see Figure 13 a), confirming a pitch of 15 nm for compound **1/14**.^20^ The pitch of almost three times the layer distance is not exactly commensurate with a three‐layer helix (see Figures 3, 13b, c and Table 1). The observation of only one resonant scattering without umklapp peak confirms the formation of a SmC_α_*‐like heliconical phase with incommensurate 2.8‐layer periodicity (Sm(CP)_α_). Herein we prefer to use Sm(CP)^hel^ as a general phase assignment of heliconical smectic phases, also including possible commensurate phase types.

In addition, the RSoXS investigation indicated the persistence of a local helical structure with a pitch between 13 and 15 nm (between two and three layers) in the birefringent SmC_a_P_A_ range of **1/14** below the Sm(CP)^hel^ phase from 99 °C down to 83 °C (Figure 13 a, encircled diffuse scattering).^20^ The decreasing intensity of the diffuse scattering is associated with an increasing intensity and sharpening of the resonant scattering corresponding to the double layer periodicity (*d*_02_). This short range heliconical structure is assumed to contribute to the special properties of this SmC_a_P_A_^(hel)^ phase occurring in the vicinity of the heliconical smectic phase.

### Effects of the heliconical structure on the switching mechanism

**Figure 14:**
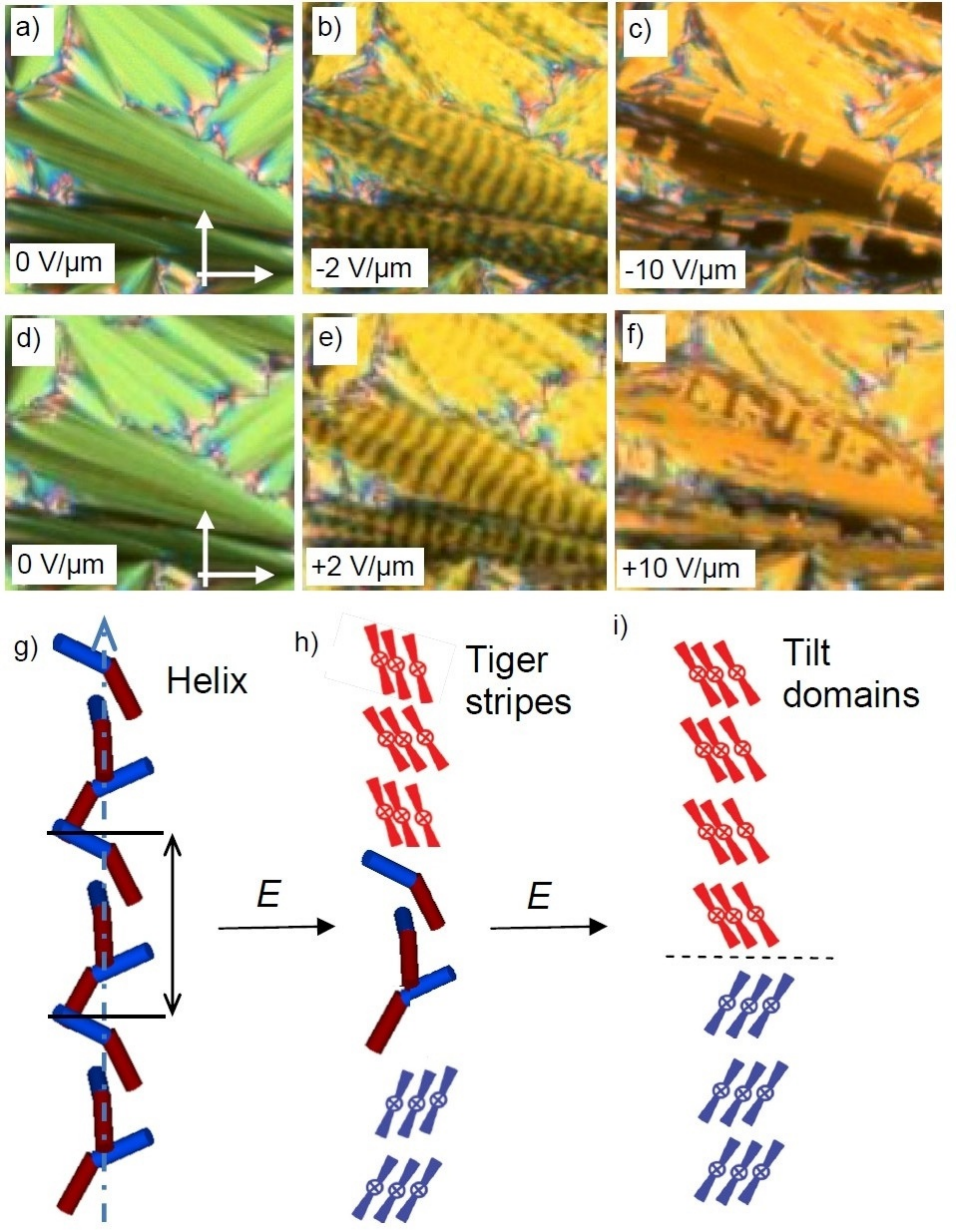
Electrooptical investigation of the switching process of compound **1/12** in planar alignment (6 μm PI coated ITO cell) under an applied DC field in the Sm(CP)^*hel*^ range at *T=*105 °C with models of the distinct field‐induced states; the width of the images is 0.2 mm.

Two distinct textures were induced in the temperature range of the Sm(CP)^hel^ phase by application of an electric field. For compounds **1/12** and **1/14** a unique periodic „tiger stripe“ texture develops in a limited temperature range of the Sm(CP)^*hel*^ phase under an applied electric field with a certain strength (Figure 14 b, e).^20^ Further increasing the applied voltage leads to the typical tilt domain texture indicating field induced SmC_s_P_F_ states with opposite tilt direction (Figures 10 b and 14 c, f). The transition from tiger stripes to tilt domains is shifted to higher temperature from **1/12** to **1/14**; for **1/12** the tiger stripes were observed around the middle of the Sm(CP)^*hel*^ phase, for **1/14** they can only be found at the transition from SmAP_R_ to Sm(CP)^*hel*^ and for **1/16** no tiger stripes and exclusively large tilt domains can be observed. In the same sequence the threshold fields for tiger stripe formation and tilt domain formation decrease, meaning that the synclinic tilted states become stabilized with growing chain length, being in line with the observation of a SmC_a_P_A_‐SmC_s_P_A_ transition by further alkyl chain elongation from **1/16** to **1/18** (see Figure 3). The dark and bright tiger stripes (Figures 14 b, e and S19) appear to represent regular arrays of SmC_s_P_F_ layer stacks with opposite tilt direction and chirality. The periodicity obviously results from a residual long pitch heliconical organization in the micrometer range, after partial unwinding of the short pitch heliconical Sm(CP)^*hel*^ structure (Figure 14 h). Complete unwinding of the helix at higher voltage leads to the tilt domains, indicating the formation of field stabilized SmC_s_P_F_ states (Figure 14 c, f, i), switching by precession on a cone between the two oppositely tilted polar states.

**Figure 15:**
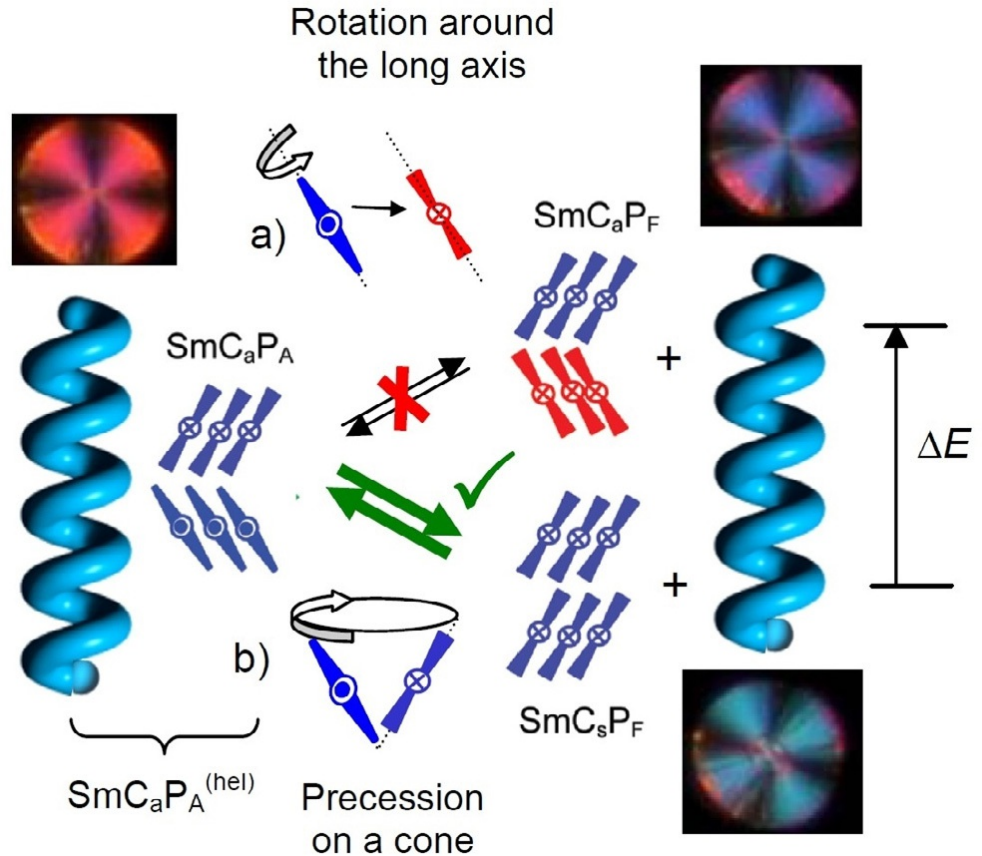
Diastereomeric relations between layer chirality and helix chirality (blue/red) in the SmC_a_P_A_^(hel)^ phase with short‐range helix; the blue‐blue interactions are arbitrarily assumed to be low energy whereas blue‐red combinations are considered to have higher energy (the used textures are only for illustration). a) Switching by rotation around the long axis reverses the layer chirality in half of the layers, which becomes chirality inverted and thus incompatible with the helical chirality; this leads to a higher energy for this diastereomeric pair between residual helix and developing layer chirality. b) Switching by precession on a cone retains the layer chirality and the low energy diastereomeric pair is retained during switching.

It appears that the helix modifies the mode of switching and that the influence of the helical superstructure increases with growing alkyl chain lengths from **1/12** 82 to **1/16** and with decreasing temperature. For compound **1/14** the switching with formation of field induced tilt domains takes place in the whole Sm(CP)^*hel*^ phase range and it is retained even in the adjacent SmC_a_P_A_^(hel)^ phase with short range heliconical organization, occurring below the Sm(CP)^hel^ phase.^20^ This means that in this case the helix clearly hinders the intrinsically preferred rotation around the long axis (see electro‐optical investigations), because this process would invert the layer chirality of every second layer, thus leading to the racemic SmC_a_P_F_ structure where half of the layers assumes the thermodynamically less stable (higher energy) state of the diastereomeric pair of layer chirality and helix sense (red layer+blue helix, Figure 15 a). Therefore, the presence of a short pitch helix changes the switching mechanism from SmC_a_P_A_↔SmC_a_P_F_ by rotation around the long axis (chirality flipping) to SmC_a_P_A_↔SmC_s_P_F_ by a precession on a cone, which retains the layer chirality of all layers, but flips the optical axis (optical switching, Figures 10 b and 15 b). Hence, in this class of compounds the switching by precession on a cone is an indication of a long‐ or short‐range helical organization. Upon further cooling of compound **1/14** the short range helix is completely removed at 83 °C, as indicated by the disappearance of the diffuse resonant scattering (Figure 13 a).^20^ The optical axis remains parallel to the layer normal, together with the *d*_02_ resonant scattering indicating an anticlinic tilted smectic phase. The loss of the helix leads to a change of the mode of switching from precession on a cone to a rotation around the long axis (Figure S25). For the shorter and longer homologues **1/12** and **1/16** no SmC_a_P_A_^(hel)^ phase is formed and the helix is completely removed already at the Sm(CP)^hel^ to SmC_a_P_A_ transition.

### The non‐helical low temperature phases of compounds 1/14–1/20

#### The helix‐free low temperature SmC_a_P_A_ phase range

In the SmC_a_P_A_ phases of **1/8**–**1/14** the two polarization current peaks become increasingly non‐symmetric on cooling (Figures 9, S9, S14 and S17) and can even fuse to a non‐symmetric broad single peak (Figures 9 g→h→i and S17). We attribute the broadening to a growing packing density with lowering temperature (see Figure 17), which slows down the switching. The unsymmetrical shape of the current peaks could indicate the coexistence of different switching mechanisms.

**Figure 16:**
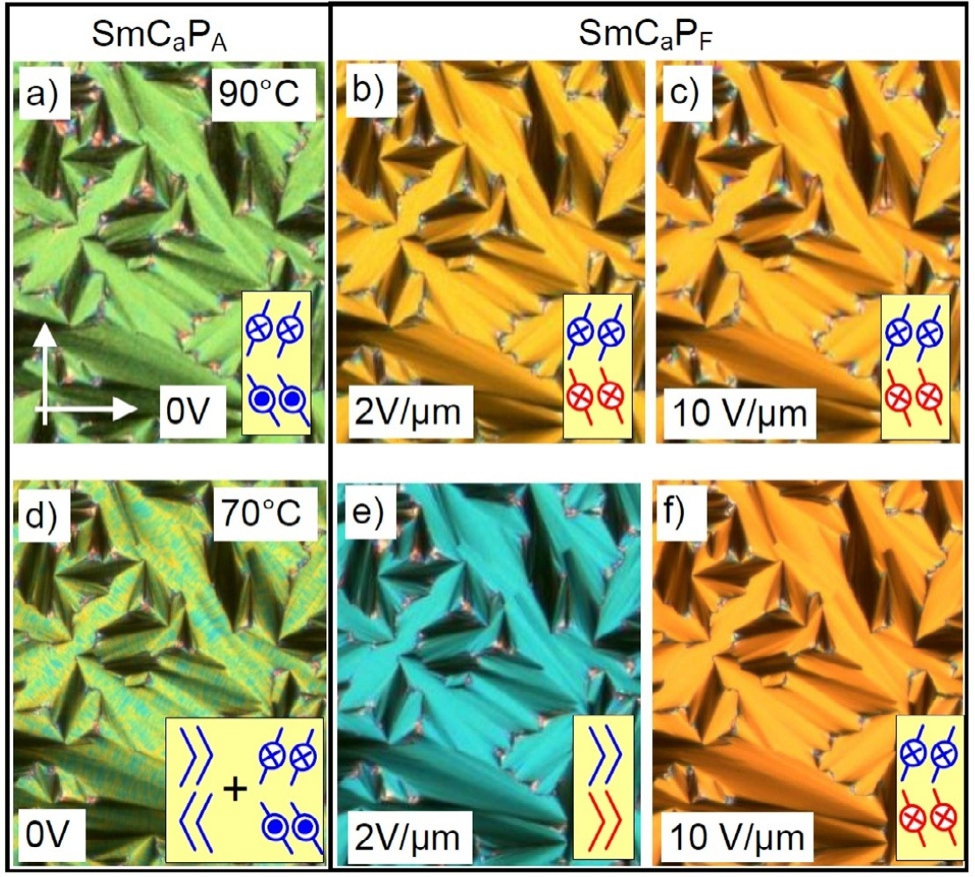
Electrooptical investigation of the switching process of compound **1/12** under an applied DC field in the SmC_a_P_A_ phase; a–c) at 90 °C and d–f) at 70 °C; the switching is fully reversible at both temperatures; in d) the speckled texture is composed of coexisting areas of the SmC_a_P_A_ phase with the bow planes parallel and perpendicular to the surface, whereas in e) all bow planes in the SmC_a_P_F_ state are parallel and in f) perpendicular to the surfaces; the width of the images is 0.5 mm; for more details of this switching process, see explanations associated with Figure S20.

In the whole SmC_a_P_A_ range the position of the extinctions does not change, but as shown in Figure 16 d–f for **1/12**, below 80 °C an additional low birefringent state (blue, Figure 16 e) is formed under low applied field before the transition to the high birefringent SmC_a_P_F_ state at higher voltage (orange, Figure 16 f). This low birefringent intermediate state is explained by the formation of a field‐induced ferroelectric state having an orientation of the bow‐planes predominately parallel to the surfaces,^83^ which realigns into an orientation perpendicular to the surfaces (having higher birefringence) at further increased voltage (Figure 16 e→f).

It appears that with growing chain length (**1/8→1/16**) and decreasing temperature there is a stabilization of the anchoring of the molecules with the bow‐planes parallel to the surface which is obviously associated with a stabilization of the SmC_a_P_F_ state at lower temperature (see below). For example, a surface stabilized (SS) SmC_a_P_F_ state was observed for the SmC_a_P_A_ phase of **1/16** 57 and SS ferroelectric switching was proposed for the helix‐free SmC_a_P_A_ phase of **1/14** (designated as SmC_a_P_F_ in ref. 20). However, because the polarization peak is not symmetric and the texture returns back to the speckled ground state texture after removal of the field (Figure S25 and ref. 20) we still consider the switching as antiferroelectric, and therefore we retain the phase assignment as SmC_a_P_A_. This is in line with a relatively high threshold voltage observed for this switching.^57^

However, for **1/16** a symmetric single polarization current peak forms on further cooling below 83 °C (Figure 19 f). Upon further chain elongation, starting with compound **1/18**, the synclinic tilt becomes dominating and the SmC_a_P_A_ phase is replaced by a synclinic SmC_s_P_A_ phase (Figure 3). Also for this compound an asymmetric double peak develops on cooling (Figure S36), which on further cooling turns into a symmetric single polarization current peak at 85 °C (Figure 21 p).

### The non‐tilted ferroelectric low temperature SmA′P_F_ phase of compounds 1/16–1/20

**Figure 17:**
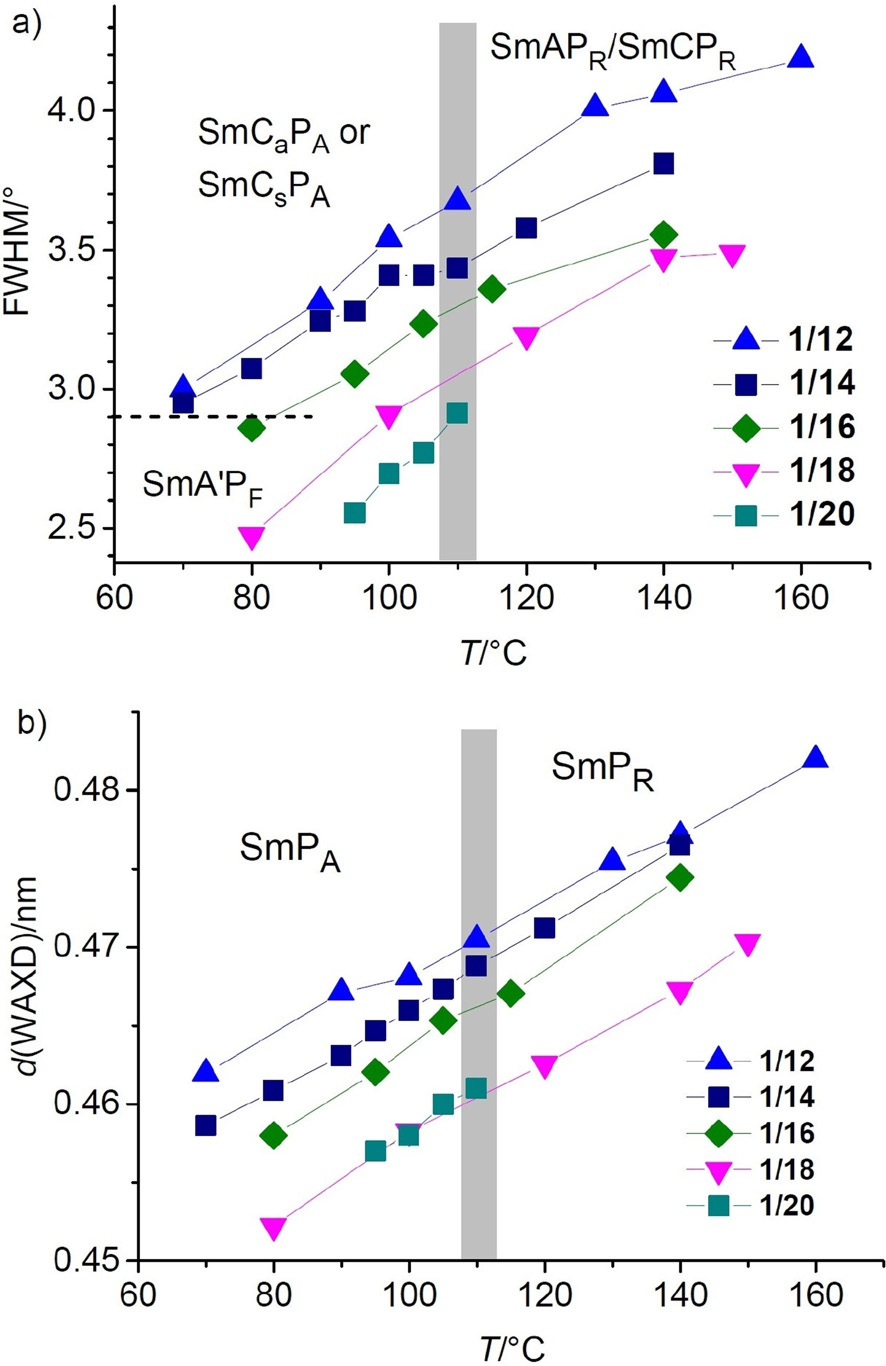
a) FWHM and b) position of the scattering maximum of the WAXS of compounds **1/*n*** depending on chain length and temperature, both indicating a growing packing density with decreasing temperature; the gray line indicates the paraelectric‐(anti)ferroelectric transition range.

**Figure 18:**
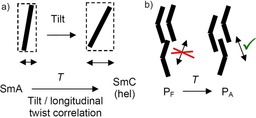
Schematic sketches a) of the effect of tilt on the strength of interlayer tilt‐ and twist‐correlation and b) the effect of inter‐layer fluctuations on the mode of polar order; the double headed arrows in a) indicate the secondary director and in b) the direction of the fluctuations.

**Figure 19:**
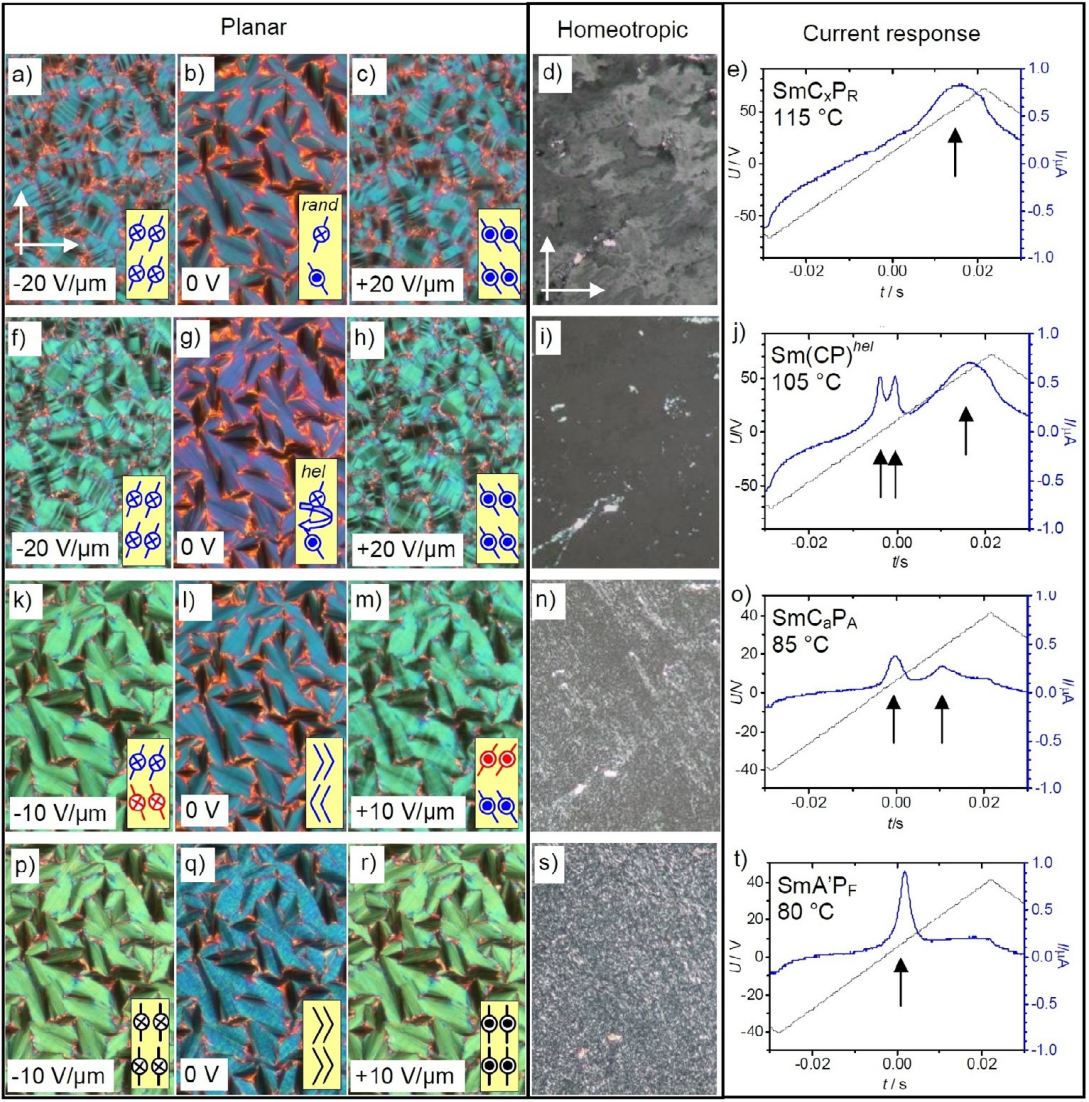
Electro‐optical investigation of the LC phases of compound **1/16**, a–e) SmC_x_P_R_ range at 115 °C (rand=randomized), f–j) Sm(CP)^hel^ range at 105 °C, k–o) SmC_a_P_A_ range at 85 °C and p–t) SmA′P_F_ phase at 80 °C; the width of the images is 0.4 mm for the planar textures and 0.6 mm for the homeotropic textures; see also Figures S31 and S32.

The development of a symmetric single polarization peak for compounds **1/16** and **1/18** is obviously associated with a transition from the anticlinic tilted SmC_a_P_A_ phase to a non‐tilted smectic phase (SmA′P_F_) taking place on cooling.^57^ This transition is only recognizable by a slight increase of the birefringence in the ground‐ and field‐induced states, whereas the extinctions retain their position parallel to the layer normal (Figures 19 k–m and p–r, S32, S39, S40, S42, S46, and S50) and no peak can be observed in the DSC for this transition (Figures 6 c, S26 and S43). This unusual inverted temperature dependence of the SmC‐SmA transition was discovered and described for **1/16** in more detail in ref. 57 and is shown to be associated with a decreasing tilt angle with lowering temperature. Below a certain critical tilt angle the secondary director might become too short for the transmission of the helical correlation between the layers (see Figure 18 a). The decreasing tilt is associated with a narrowing of the full width at half maximum (FWHM) of the wide angle scattering (Figure 17 a), indicating that the correlation length of in‐plane order grows continuously. Below a line width (FWHM) of about 2.9° the transition to the SmA′ phase takes place (Figure 17 a). That the increase of the packing density can indeed lead to a transition from tilted to non‐tilted lamellar phases is known for SmC*‐HexB* transitions.^84^ However, in our case the change of the line shape of the wide angle scattering is continuous and a Lorentzian line‐shape is retained,^85^ and therefore, we still consider the SmA′P_F_ phase as a smectic phase with enhanced in‐plane order instead of a hexatic phase with long range bond orientational order (HexB).^86^ Indeed, non‐polar reentrant SmA phases have previously been reported for chiral rod‐like LC compounds^87^ and an achiral mesogenic dimer.^88^

Associated with the transition to SmA′P_F_ the shape of the polarization current peak changes from a non‐symmetric double peak to a relatively sharp and fully symmetric single peak, which is especially well developed for **1/16** and **1/18** (Figures 19 o→t, 21 l→p, S31 and S36). This symmetric single peak, occurring shortly after zero‐voltage crossing, is a first indication of a ferroelectric switching (SmA′P_F_ phase). In line with a transition to ferroelectric switching the threshold voltage of the switching significantly decreases at the SmC_a_P_A_‐SmA′P_F_ transition (Figure 21).^57^ The reason for the growing contribution of SS ferroelectric switching with lowering temperature might be the reduced importance of interlayer fluctuations, entropically stabilizing the antiferroelectric state at higher temperature (Figure 18 b).^41^, ^89^ The reduced contribution of this entropic effect at lower temperature allows easier formation of polar (ferroelectric) order, which is then fixed by polar surface anchoring. Though there is a clear single peak switching, the birefringence decreases after switching off the applied field, indicating that after release of the applied field the bow‐planes assume an orientation parallel to the surfaces (Figure 19 p–r), as previously observed for the SmAP_F_ phases of silylated bent‐core molecules.^83^ For compounds **1/16** and **1/18** the SmA′P_F_ phase is formed below the anticlinic SmC_a_P_A_ phase, whereas for the longer homologue **1/20** there is a transition from the *synclinic* tilted SmC_s_P_A_ phase (see below) to SmA′P_F_. This means that the transition to the SmA′P_F_ phase is independent on the actual tilt correlation between the layers in the adjacent smectic phase as long as it is weak. The removal of the tilt and the stabilization of the ferroelectric state in the SmA′P_F_ phase are associated with the anticlinic to synclinic transition taking place by chain elongation from *n=*16 to 20. Thus, the SmA′P_F_ phase can be considered as an alternative intermediate structure at the transition from anticlinic to synclinic tilt, replacing the heliconical phase at lower temperature. Moreover, it provides a new alternative approach to materials with non‐tilted ferroelectric phases without requiring chain silylation.^83^

On further cooling the SmA′P_F_ phase crystallizes with formation of a crystalline phase (Cr′) having the optical axis parallel to the layer normal and showing a texture which is indistinguishable from the SmA and Sm(CP)^hel^ phases by optical investigation of planar samples (see Figure S51). Nevertheless, the high transition enthalpy values (≈60 kJ mol^−1^), the significant hysteresis of the phase transition (≈20 K, see Figure S43 and Table S1) and the impossibility to shear the sample, confirm a crystalline phase. For compound **1/22** the SmA′P_F_ phase is completely replaced by Cr′ (Table 1 and Figure 3). The XRD pattern of the Cr′ phase of **1/22** indicates a well developed lamellar structure with a layer spacing almost identical with that in the adjacent SmC_s_P_a_ phase (*d=*6.67 nm) and a 32–34° tilted organization of the molecules (see Figures S53 and S54). Despite of the tilt, the optical axis is parallel to the layer normal which requires an anticlinic or short pitch heliconical structure of this crystalline lamellar phase.

### Compound 1/16–emergence of tilt correlation in the paraelectric SmC_x_P_R_ phase

On cooling compound **1/16** a long range tilt develops already in the paraelectric smectic phase range as indicated by the transition to a weakly birefringent texture in homeotropic alignment at *T=*125 °C (Figure 19 d), that is, 15 K above the paraelectric‐(anti)ferroelectric transition. This means that starting with **1/16** tilt correlation sets in already before development of long range polar order. In the planar texture between 125 °C and the paraelectric‐(anti)ferroelectric transition at 108/110 °C (cooling/heating) the orientation of the extinctions is parallel to the polarizers (Figure 19 b). Application of an electric field to the planar sample leads to a tilt‐domain texture, indicating the switching into a polar state with synclinic tilt (SmC_s_P_F_) which relaxes back to the original smooth fan texture of the polarization randomized phase upon removing the applied field (Figure 19 a–c). This indicates a switching by precession on a cone and thus is likely to indicate that a short‐ or long‐range heliconical structure could already develop in the high permittivity paraelectric range. In the polarization current curves there is only one broad maximum, indicating a ferroelectric‐like paraelectric switching between 125 °C and the transition to the antiferroelectric phase around 110 °C (Figure 19 e),being typical for polarization randomized (P_R_ type) smectic phases.^46^, ^47^ This phase, tentative designated as SmC_x_P_R_, requires further clarification by RSoXS investigations. It transforms to the uniaxial Sm(CP)^hel^ phase at around 110 °C, as indicated by the disappearance of birefringence in the homeotropic samples (Figure 19 d→i), by the retention of a smooth fan texture with extinctions parallel to the polarizers (Figure 19 b→g) and by the emergence of two additional close polarization current peaks under a triangular wave voltage (Figure 19 j). The resulting three peak switching (two sharp and one broad) is considered as a ferrielectric switching as typical for heliconical smectic phases with three‐layer periodicity.^17^, ^18^, ^20^ For this compound the long range helical Sm(CP)^hel^ structure is stable down to 92 °C. At this temperature the transition to the SmC_a_P_A_ phase takes place and the two close polarization peaks merge to one, leading to the unsymmetrical double peak feature of the helix‐free low‐temperature SmC_a_P_A_ phase (Figure 19o→t).

### The long chain compounds 1/18–1/22: Developing synclinic tilt and the field‐induced heliconical phase

**Figure 20:**
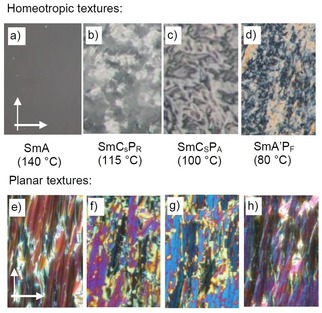
Textures of the LC phases of **1/18** at the indicated temperatures in the ground state: a–d) in homeotropic alignment and e–h) in planar alignment; the increased birefringence in d) is partly due to a change of the alignment; the width of the images is 0.2 mm for the planar textures and 0.5 mm for the homeotropic textures.

**Figure 21:**
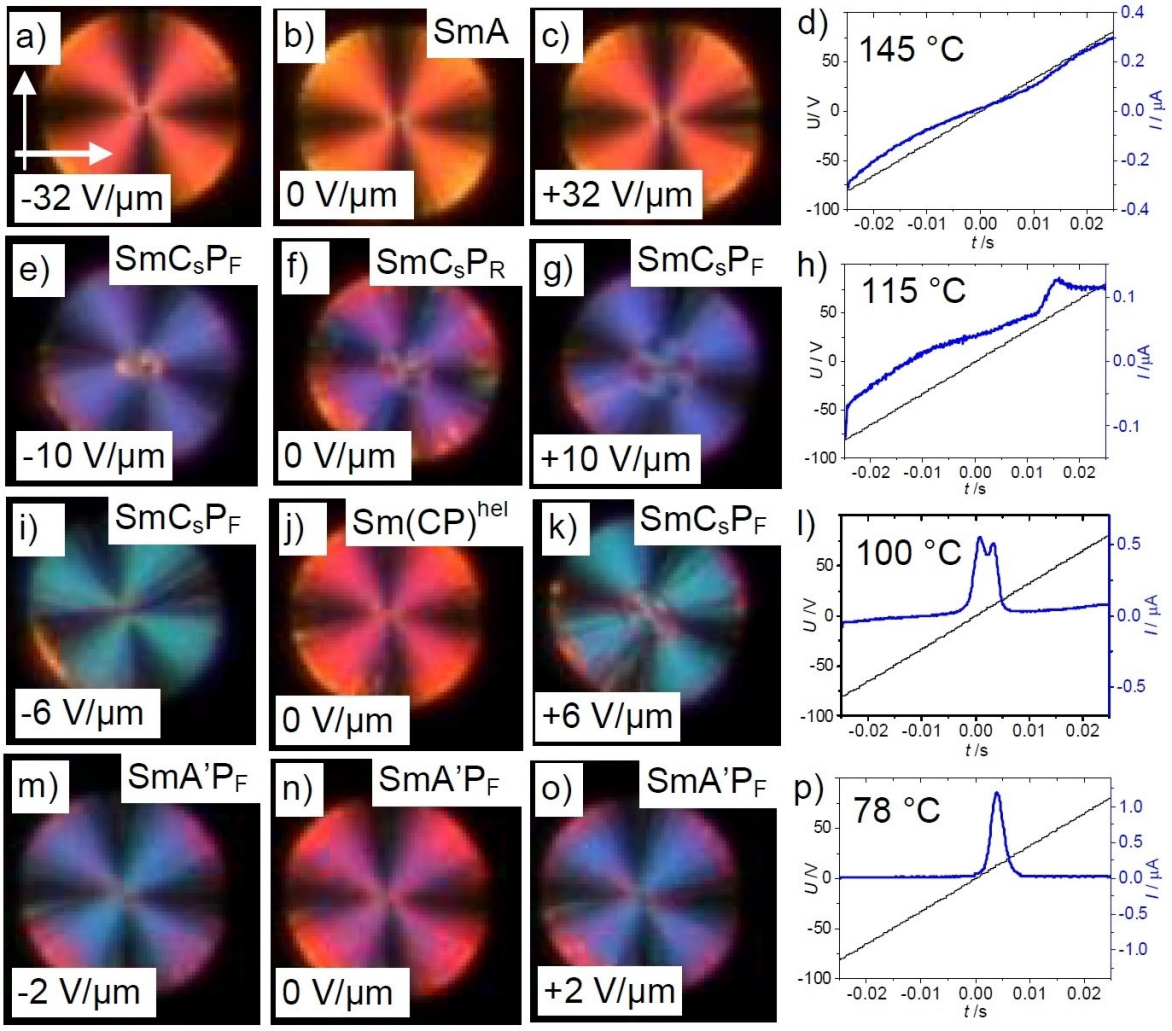
Electro‐optical investigation of the smectic phases of compound **1/18** at the indicated temperatures (6 μm non‐coated ITO cell, 160 V_pp_); a–d) SmA phase, e–h) SmC_s_P_R_^[^*^]^ phase, i–l) Sm(CP)^hel^ phase and m–p) SmA′P_F_ phase, with corresponding polarization current curves (right columns); the width of the images is 0.1 mm; see Figures S36–S42 for more details.

For the next homologue **1/18** phase biaxiality sets in already at 134 °C, in the paraelectric range (Figure 20 a→b). In this case the dark extinction brushes incline with the directions of the polarizers in the planar samples (Figures 20 e→f, 21 f). This confirms a uniform (synclinic) tilt emerging at 134 °C which is on further cooling retained across the paraelectric‐(anti)ferroelectric transition at 107/110 °C (cooling/heating) down to the transition at 85 °C without formation of a heliconical phase. In the paraelectric SmC_s_P_R_ phase the inclined extinction crosses do not rotate by inverting the field direction (Figures 21 e–g and S42), indicating a switching by rotation around the long axis and confirming the absence of a helix in the planar samples.^90^

However, the paraelectric and biaxial smectic phase between 110 and 135 °C shows the formation of a conglomerate of chiral domains in homeotropic alignment (Figure S34) which, together with the single broad polarization peak under a triangular wave field (Figure 21 h), confirms a high permittivity paraelectric SmC_s_P_R_^[^*^]^ phase. The optical rotation in this SmC_s_P_R_^[^*^]^ phase is assumed to be caused by surface assisted alignment of the ferroelectric SmC_s_P_F_ grains with a certain size to enable the formation of macroscopic SmC_s_P_F_ domains, then assuming a long‐pitch heliconical organization between the cell surfaces.^46^, ^47^, ^48^, ^49^ Thus, the SmC_s_P_R_^[^*^]^ phase is considered as a long pitch analogue of the short pitch Sm(CP)^hel^ phase with some similarity to the partly unwounded helical state, being responsible for the tiger stripe texture. This is a first indication that heliconical phases can also appear if the phase structure is SmC_s_P_F_. It also indicates that helix formation can be supported or suppressed by surface anchoring.

At the small DSC peak around 110 °C two broad polarization peaks emerge and develop into a pair of close peaks while the synclinic tilt is retained (Figures 20 g and 21l), indicating that the polar phase is no more an anticlinic SmC_a_P_A_ phase as that of the shorter homologues, but has changed into a synclinic tilted SmC_s_P_A_ phase for **1/18** and the following homologues.^59^ In this SmC_s_P_A_ phase application of an AC‐field, followed by switching off the field leads to a decrease of birefringence (blue to red, see Figure 21 i–k) and the orientation of the extinction crosses at 0 V becomes parallel to the polarizers, indicating a field‐induced transition to a state with the main optical axis parallel to the layer normal (Figure 21 j) as known for the Sm(CP)^hel^ phases. That a heliconical Sm(CP)^hel^ phase is indeed induced by the electric field is confirmed by the helix deformation (DHF effect, V‐shaped switching) and a helix unwinding to a field‐induced polar SmC_s_P_F_ state upon application of a DC field to a planar sample (Figure 21 i–k), being identical to that observed for the Sm(CP)^hel^ phase of **1/16**.^19^, ^60^, ^61^ Field‐induced and field modified helical structures are known for LC systems formed by permanently chiral molecules,^13^ but to the best of our knowledge there is only one recent report about field induced helix formation in a (nematic) LC phase of achiral molecules.^91^ The SmC_s_P_A_ phase capable of assuming a field induced helix is found for all compounds **1/18**–**1/22** with long chains, though the temperature range of the field‐induced helical state decreases with chain elongation. The heliconical Sm(CP)^hel^ phase is induced in the whole SmC_s_P_A_ range of all three compounds, but only for **1/18** it is retained in the whole SmC_s_P_A_ range also after removing the field. For **1/20** and **1/22** it is only stable in the upper temperature range of the SmC_s_P_A_ phase and at lower temperature it relaxes back to SmC_s_P_A_. The Sm(CP)^hel^‐SmC_s_P_A_ transition temperature, which is easily determined by the change of the extinction direction under the polarizing microscope, rises with growing chain length, meaning that with growing synclinic tilt correlation the field‐induced Sm(CP)^hel^ range tightens (see Figure 3).

### The long chain compounds 1/18–1/22—paraelectric SmC_s_P_AR_ phase and inversion of birefringence

In contrast to **1/18**, for compounds **1/20** and **1/22** the paraelectric SmC_s_ phase above the SmC_s_P_A_ phase does not show chiral domains under any conditions. However, two widely separated broad current peaks emerge about 5 K above the transition to the polar smectic phase (Figures S48 and S55), indicating a short SmC_s_P_AR_ range. The antipolar coupling between the ferroelectric grains leads to a racemic structure and thus might inhibit the development of a surface stabilized helix in the whole paraelectric SmC_s_ range.

As another typical feature of the synclinic tilted phases of compounds **1/18**–**1/22** there is an inversion of birefringence in the vicinity of the paraelectric‐(anti)ferroelectric transition (see Figure S35 and S47). This is assumed to be due to the changing orientation of the secondary optical axis, which depends on polarization and tilt which are perpendicular and compete with each other in the polar SmC phases of bent‐core molecules.^52^ This inversion takes place around or a few degrees below the paraelectric‐(anti)ferroelectric transition, at 110 °C for **1/18**, at 98 °C for **1/20** and at 88 °C for **1/22**. The shift to lower temperatures becomes larger with growing alkyl chain length, that is, with growing synclinic tilt correlation. Thus, the direction of the main optical axis is likely to be mainly determined by the tilt at higher temperature and at lower temperature by the direction of polar order, becoming stronger with growing packing density.

### Understanding of heliconical phase formation based on stereochemical rules

**Figure 22:**
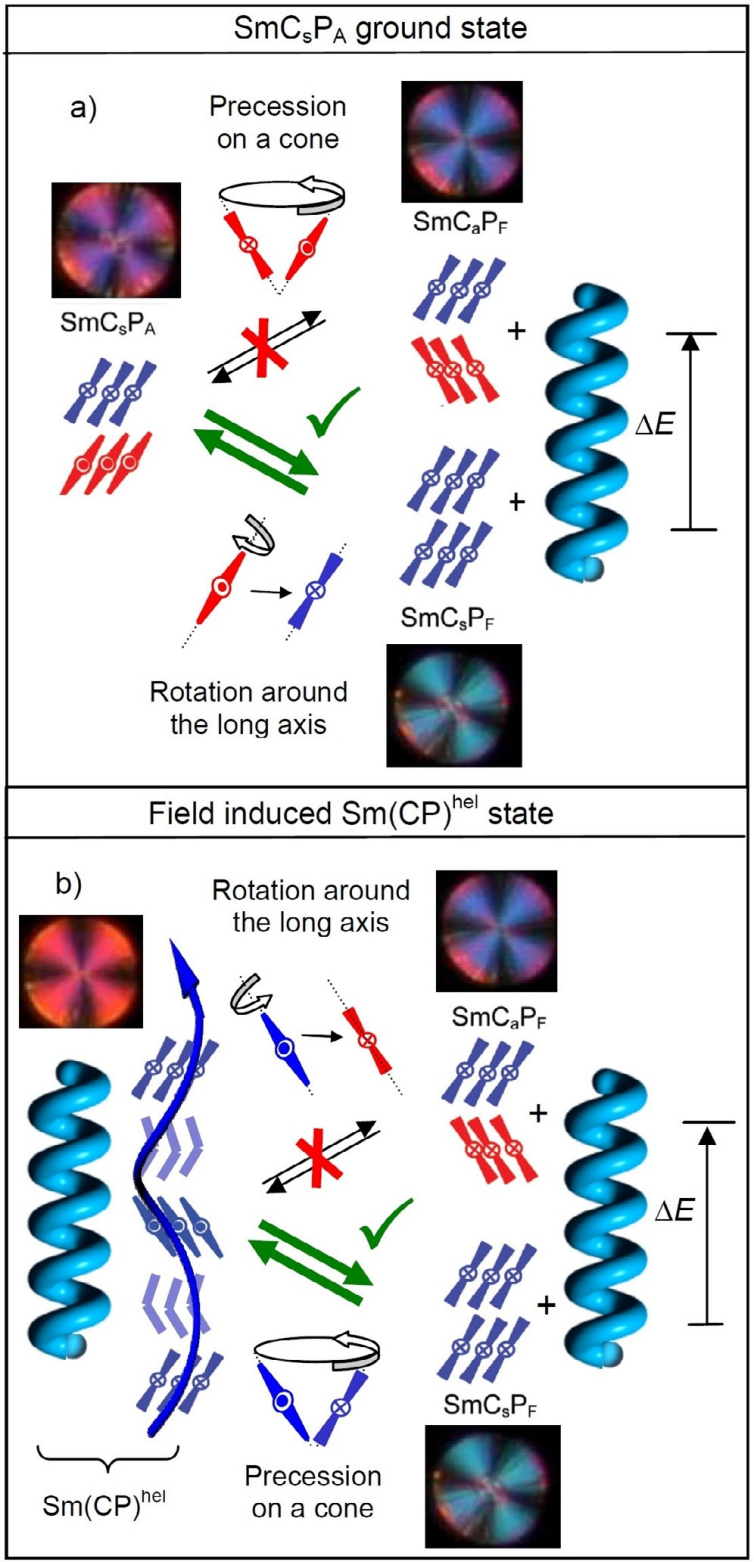
Diastereomeric relations between layer chirality and developing helix chirality in the SmC_s_P_A_ phase. a) Starting with the ground state, the rotation of the molecules in every second layer takes place around the long axis which changes the SmC_s_P_A_ ground state to the field induced SmC_s_P_F_ state; this relaxes to the heliconical Sm(CP)^hel^ structure after removal of the field; the alternative precession on a cone would lead to a racemic SmC_a_P_F_ structure which is incompatible with helix formation. b) For the field‐induced Sm(CP)^hel^ phase the switching by rotation around the long axis reverses the chirality of every second layer and thus would lead to a racemic structure, which is incompatible with the residual helix, whereas precession on a cone retains the chirality sense as well as the energy minimum diastereomeric state between layer chirality and helix sense (the shown textures are only for illustration of the expected optical response).

As shown in the previous Sections, helix formation in the ground state takes place in the SmC_a_P_A_ phase, having uniform layer chirality, but as soon as the tilt changes from anticlinic to long range synclinic (from *n=*16 to 18) the Sm(CP)^*hel*^ phase is completely removed and replaced by a non‐helical SmC_s_P_A_ phase. This phase is racemic with alternating layer chirality between the layers and this is incompatible with helix formation, because the diastereomeric pair SmC_s_P_A_+helix would have a higher energy than SmC_a_P_A_+helix (see Figures 15 and 22 a; any red‐blue combination is considered to have higher energy).

However, as soon as the chiral SmC_s_P_F_ structure is induced under an applied E‐field (SmC_s_P_A_ to SmC_s_P_F_ transition by rotation around the long axis) a helix can develop in the field‐induced chiral SmC_s_P_F_ structure (Figure 22 a) by a helical distribution of the polar layers during removal of the field (escape from macroscopic polarization). This field induced heliconical phase has the same structure composed of SmC_s_P_F_ layers twisted around a helix axis parallel to the layer normal as the spontaneously formed Sm(CP)^*hel*^ phase and can be considered as the common degenerated helix structure derived from any of the two polar smectic phases with uniform chirality, either the antiferroelectric SmC_a_P_A_ or the ferroelectric SmC_s_P_F_ phase; it makes no difference from which of the two phases it is formed. Based on stereochemical principles, there is an inherent driving force for helix formation associated with the homochirality of the SmC_a_P_A_ and SmC_s_P_F_ phases. Usually it develops as a transversal twist leading to frustrated smectic phases (HNF, DC12b, 30, 39). The alternative longitudinal twist along the layer normal, reported here, can obviously only develop if the interlayer coupling is sufficiently weak, that is, close to the transition from a de Vries SmA phase to a weakly tilted SmC phase being close to the transition between anticlinic and synclinic tilt. The heliconical structure once formed (in the ground state or as a field‐induced state) disfavors the chirality inverting switching around the long axis, which would lead to a racemic state, whereas the precession on a cone retains the layer chirality (Figure 22 b). Therefore, in the presence of a helix the switching process changes from chirality flipping by rotation around the long axis to the chirality preserving rotation on a cone. Thus, the complex phase behavior of the homologous series of compounds **1/*n*** can be understood by the fundamental principles of stereochemistry.

### Relations with other heliconical phases

The heliconical Sm(CP)^*hel*^ phase has some similarity with the long pitch heliconical SmC_s_* and SmC_a_* phases formed by permanently chiral rod‐like molecules and especially with the short pitch SmC_α_* phase formed by molecules with high enantiomeric purity, known as a incommensurate heliconical phase with 5–50 layer pitch.^17^ In common with the Sm(CP)^*hel*^ phase its formation is associated with a small tilt in the SmC* phases (*β*≈8°) and it occurs at the transition from SmA* to synclinic SmC_s_* which on further cooling form anticlinic SmC_a_* phases.^17^ Hence, both series of compounds have only weak coupling of the tilt between the layers, combined with a molecular chirality, being either permanent or transient. In the case of the SmC* phases of rod‐like mesogens a permanent molecular chirality biases a specific sense of the helical molecular conformation^92^ whereas for the achiral bent‐core mesogens the pronounced transient helicity of the conformers of bent‐core mesogens^31^ is responsible for the helix formation. The helix is energetically favored as it leads to a denser packing of the molecules than in the disordered structure in the absence of the helix. The developing helix sense is arbitrary (stochastic) in the case of achiral molecules.^12^ The synchronized helical conformers prefer a correlation of the tilt between the layers being unequal to 0° (synclinic) or 180° (anticlinic) thus leading to an intermediate angle, providing the helical twist between the layers. A weak synclinic or anticlinic layer coupling, together with the inherent layer chirality of SmC_a_P_A_ and SmC_s_P_F_ phases, support the development of the helical twist either in the ground state (SmC_a_P_A_) or in the field‐induced (SmC_s_P_F_) state. The helix pitch is about only 3 layers as this allows a twist of around 120° between adjacent layers. This angle is presumably close to that provided between the alkyl chains in the relevant helical conformations,^31^ thus allowing easy fluctuations of the molecules between adjacent layers and leading to an entropic advantage. Commensurate heliconical phases with 3‐ or 4‐layer pitch are known to occur for rod‐like molecules with high enantiomeric purity at the transition between SmC_s_* and SmC_a_* phases (SmC_FI1_*, SmC_FI2_* phases).^14^, ^17^, ^18^ It is possible that besides the incommensurate SmC_α_*‐like helix also structures with SmC_FI_‐like commensurate 3 and 4‐layer or other periodicities could be found for the transiently chiral bent‐core molecules.

Recently, heliconical smectic phases (designated as SmC_TB_) have also been reported for bent mesogenic dimers; in this case predominately commensurate 4‐layer structures have been reported for these non‐polar smectic phases.^67^ That the pitch in the Sm(CP)^hel^ phases of the bent‐core compounds **1/*n*** (<3 layers) is shorter than in the SmC_TB_ phases of bent dimesogens (4 layers) might be the result of the increased helicity of the molecular conformations of the bent‐core mesogens, and especially due to the possibility to escape from the significant macroscopic polarization occurring in the layers of their polar smectic phases.^93^, ^94^ For the bent‐core molecules the combination of polar order and helicity provides the unique possibility of affecting the helical structure by application of external electric fields, leading to numerous potential applications.^13^, ^79^, ^80^ The V‐shaped switching due to helix deformation (DHF effect)^19^, ^60^ and an extremely fast optical switching between biaxial and optical uniaxial states in homeotropic device configurations^79^ represent first examples for such applications.

## Conclusions

A full series of 4‐cyanoresorcinol bisterephthalates **1/*n*** with chain lengths ranging from *n=*2 to 22 was synthesized and investigated with respect to LC self‐assembly and spontaneous development of heliconical superstructures. This allowed an understanding of the development of the complex phase sequence observed in this unique series of compounds, which was not possible in previous work considering only some individual compounds.

**Figure 23:**
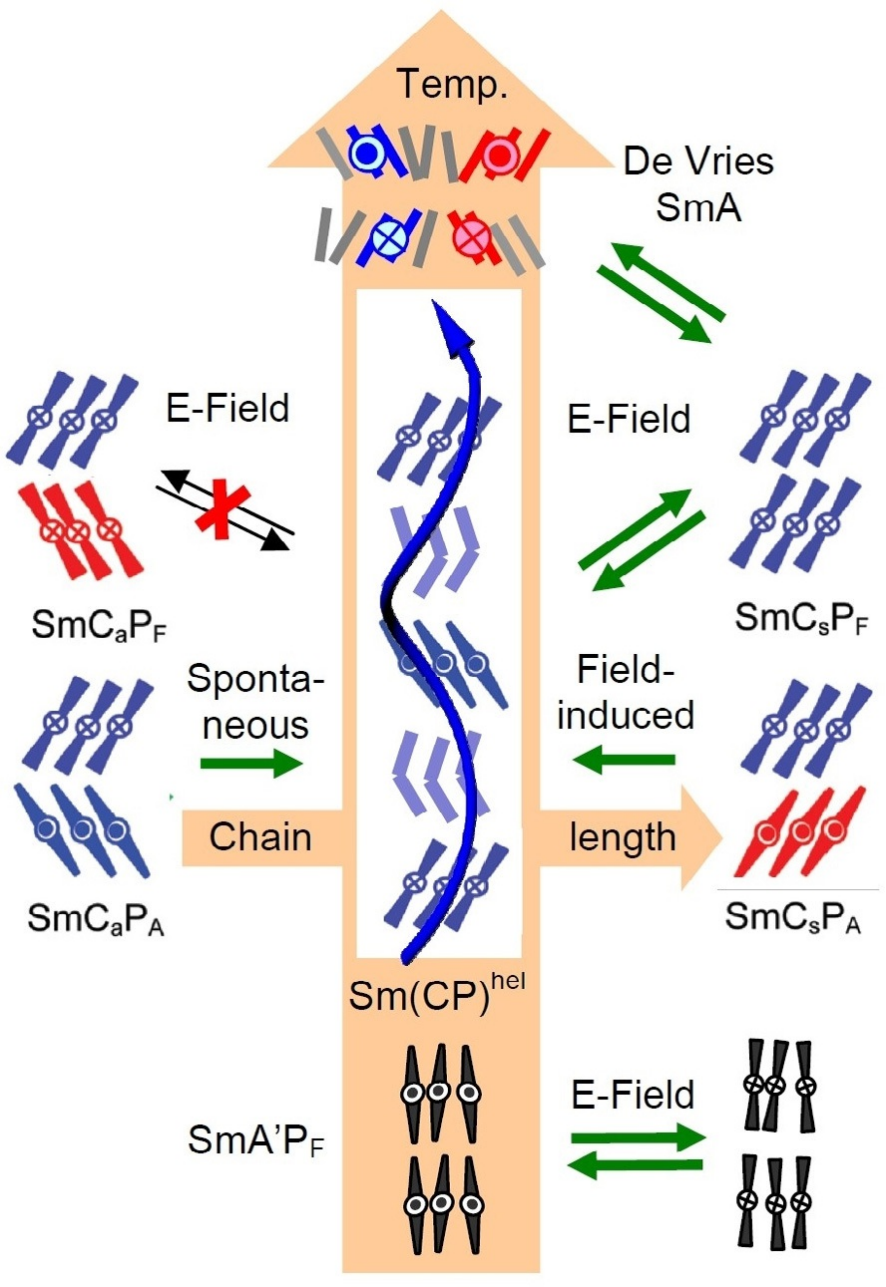
Summary of the smectic phases of compounds **1/*n*** depending on chain length and temperature and their field‐induced transformations.

All compounds with *n*≥4 form a uniaxial de‐Vries‐like SmA phase at high temperatures. With decreasing temperature ferroelectric grains grow, leading to paraelectric and superparaelectric smectic phases, being non‐tilted (SmAP_R_, SmAP_AR_) for relatively short chains (*n*≤14) and tilted (SmC_x_P_R_, SmC_s_P_R_, SmC_s_P_AR_) for compounds with longer chains (*n*>14). All compounds with *n*≥6 show a transition from these paraelectric and high permittivity paraelectric to polar smectic phases, in all cases indicated by a small DSC peak around 112±4 °C. The polar smectic phases below this transition are either anticlinic tilted (SmC_a_P_A_) for compounds with *n* up to 16 or synclinic tilted (SmC_s_P_A_) for those with longer chains (*n*≥18). The tilt is relatively small (15–20°) and only slightly changing with growing alkyl chain length. At the transition from the anticlinic SmC_a_P_A_ to the synclinic SmC_s_P_A_ phase new phases with unique properties emerge. At low temperature a non‐tilted and ferroelectric switching lamellar phase (SmA′P_F_) is observed for compounds with *n=*16–20, leading to the unusual SmA‐SmC‐SmA′ phase sequence with a reentrant SmA phase^87^, ^88^ on cooling. At higher temperature a short pitch heliconical smectic phase replaces this SmA′P_F_ phase for compounds with *n=*12–16, reaching its highest stability for *n=*14–16 (Figures 3 and 23). It represents a new mode of spontaneous mirror symmetry breaking in LC phases of bent‐core mesogens. In contrast to all previously reported cases with transversal twist (HNF^33^ and DC phases,^39^, ^40^, ^41^, ^42^ see Figures 1 b, f, g), for compounds **1/*n*** the twist develops longitudinal, leading to a helix parallel to the layer normal (Figures 1 a, c). There are three prerequisites for this kind of helix formation, (*i*) a polar phase with inherent layer chirality (SmC_a_P_A_ or SmC_s_P_F_), (*ii*) a weak layer coupling in the vicinity of the transition from randomized to long range tilt correlation (de Vries SmA to SmC transition) and from short range to long range polar order (paraelectric to (anti)ferroelectric transition), and (*iii*) a small tilt and weak tilt‐correlation at the synclinic‐anticlinic cross‐over. Once the Sm(CP)^hel^ phase, representing the common degenerated helical state of the SmC_a_P_A_ as well as the (field induced) SmC_s_P_F_ phase, is formed the mode of switching changes from the originally preferred chirality inverting rotation around the long axis to a chirality retaining precession on a cone, even if the heliconical structure is only short range. This is a consequence of fundamental stereochemical principles based on the diastereomeric relations between the two spontaneously and simultaneously developing modes of superstructural chirality, the layer chirality and the helix formation (Figure 23). Thus, this work shows that fundamental stereochemical rules can determine the phase structures and properties of LC systems formed by achiral compounds. This contributes to the understanding of the complex behavior of bent‐core mesogens depending on the conditions^95^ and the observations made with other weakly polar LC systems at the paraelectric–(anti)ferroelectric cross‐over, as for example, with some hockey‐stick molecules.^96^ In addition, it contributes to an improved understanding of mirror symmetry breaking in other soft matter systems, as for example the bicontinuous cubic and related phases,^12^, ^97^, ^98^ the dark conglomerate phases,^25^, ^39^, ^41^, ^42^ the heliconical nematic^62^‐^66^ and smectic phases,^67^ and the spontaneously chiral isotropic liquids.^9^ Further investigations in these directions might lead to a similar diversity of different heliconical phases as known for the permanently chiral rod‐like molecules.^14^, ^17^, ^18^ Besides the fundamental scientific question of emergence of chirality in ordered fluids, these heliconical phases are of significant technological interest for fast optical switches, as tunable circular polarizing emitters, and for use as photonic materials.^13^, ^99^

## Conflict of interest

The authors declare no conflict of interest.

## Supporting information

As a service to our authors and readers, this journal provides supporting information supplied by the authors. Such materials are peer reviewed and may be re‐organized for online delivery, but are not copy‐edited or typeset. Technical support issues arising from supporting information (other than missing files) should be addressed to the authors.

Supplementary
