# Supplementary material for: Stereochemical Rules Govern the Soft Self‐Assembly of Achiral Compounds: Understanding the Heliconical Liquid‐Crystalline Phases of Bent‐Core Mesogens
Source: Chemistry. 2020 Feb 28;26(21):4714–33. doi: 10.1002/chem.201904871 (PMC7186843; doi:10.1002/chem.201904871)
Supplement: Supplementary file 1 — Supplementary [file CHEM-26-4714-s001.pdf]

Chemistry–A European Journal

Supporting Information

Stereochemical Rules Govern the Soft Self-Assembly of Achiral Compounds: Understanding the Heliconical Liquid-Crystalline Phases of Bent-Core Mesogens

Anne Lehmann,[a] Mohamed Alaasar,[a, b] Marco Poppe,[a] Silvio Poppe,[a] Marko Prehm,[a] Mamatha Nagaraj,[c] Sithara P. Sreenilayam,[c] Yuri P. Panarin,[c] Jagdish K. Vij,[c] and Carsten Tschierske*[a]

Supporting Information

Contents

1. Methods………………………………………………………………………... S2

2. Synthesis……………………………………………………………………….. S3

3. Additional Data……………………………………………………………….... S9
3.1 Phase transitions………………………………………………………………. S9
3.2 Compound **1/6**……………………………………………………………… S10
3.3 Compound **1/8**……………………………………………………………… S13
3.4 Compound **1/10**…………………………………………………………….. S14
3.5 Compound **1/12**…………………………………………………………….. S17
3.6 Compound **1/14**…………………………………………………………….. S21
3.7 Compound **1/16**…………………………………………………………….. S24
3.8 Compound **1/18**…………………………………………………………….. S27
3.9 Compound **1/20**…………………………………………………………….. S32
3.10 Compound **1/22**…………………………………………………………… S37

4. Development of phase assignments of compounds 1/*n*………………………… S41

5. References……………………………………………………………………… S42

1. Methods

1.2 Polarizing microscopy

The particular textures of the LC phases were recorded by polarization microscopy with a DMRXP, Leica Microsystems. The sample were placed between two microscopy glass slides in a heating stage with temperature controller (Mettler FP82 HT). The textures were recorded with a Nikon Coolpix E 4500 camera or a Leica MC120HD.

1.2 DSC

The DSC investigations were carried out on a DSC 7 (Perkin-Elmer) with a constant heating and cooling rate of 10 K/min. The transition temperatures are characterized by the peak temperatures.

1.3 Switching experiments and electrooptical studies

For the switching experiments the compounds were filled in commercially available cells (EHC Japan) which consist of two glass plates with a constant distance of usually 6 µm. Afterwards the cell was placed in a heating stage (Mettler FP 82 HT) and the appropriate electric field was applied. The electric field was generated by an AC/DC generator (3322 A, Agilent). The switching response was guided through a resistance cascade (type 1435, FLC Electronics) and tracked by an oscilloscope (TDS 2014, Tectronics).

a) AC field

The AC field experiments were carried out with a triangular wave voltage at a constant frequency of 10 Hz and a resistance of 5 kΩ in PI-coated or non-coated ITO cells 6 µm distance, antiparallel rubbing and a measurement area of 1cm^2.

b) DC field

The DC field experiments were carried out with a sinusoidal wave at a constant frequency of 20 MHz and a resistance of 5 kΩ in PI-coated or non-coated ITO cells 6 µm distance, antiparallel rubbing and a measurement area of 1cm^2.

c) Determination of the spontaneous polarization

To determine the spontaneous polarization the area of the switching peak, the cell area and the resistance are needed. The determination also depends on the type of the switching response (antiferroelectric or ferroelectric case).

$$P_s = \frac{A_{peak}}{A_{cell} \cdot R}$$

- for antiferroelectric switching:

$$A_{peak} = \frac{\sum A_{i,peak}}{2}$$

- for ferroelectric switching:

$$A_{peak} = \frac{A_{i,peak}}{2}$$

1.4 Dielectric spectroscopy
The dielectric spectroscopy of **1/12** was carried out on a 10 μm planar aligned device with indium tin oxide electrodes. Experiments were done on cooling the sample from the isotropic phase from 175 °C to 50 °C, and in the frequency range between 1 Hz and 10 MHz.

1.5 XRD
X-ray investigations on powder-like samples were carried out at Cu-K_α line (λ = 1.54 Å) using standard Coolidge tube source with a Ni-filter. Samples were prepared in the isotropic state on a glass plate. The sample was cooled (rate: 5 K min^{-1}) to the measuring temperature. The samples were held on a temperature-controlled heating stage and the diffraction patterns were recorded with a 2D detector (Vantec 500, Bruker); exposure time was 15-30 min. For the WAXS measurement the distance between the sample and the detector defined to be 9.0 cm; for SAXS measurement the distance is 26.8 cm. As result the obtained XRD pattern was transformed in a 1D plot over the full Chi-range by using GADDS.

2. Syntheses

2.1 General

The compounds were synthesized according to Scheme 1. It is noted that the use of the acylation catalyst 4-dimethyaminopyridine (DMAP)[S1] should be excluded, as it catalyzes acyl group transfer reactions of the formed 4-cyanoresorcinol benzoates acting as active esters, thus leading to complex, almost inseparable mixtures. Also after isolation and purification, the remaining traces would lead to reduced thermal stability of the compounds at elevated temperature.
The purity of the compounds and intermediates was checked by thin-layer chromatography (TLC, silica gel 60 F254, Merck). Column chromatography was performed with silica gel 60 (0.063-0.2, Merck), flash-chromatography with silica gel 60 (0.040-0.063, Merck). DCM was dried over P_2O_5 and stored over molecular sieve. ^{1}H-, ^{13}C-NMR spectra (Varian Unity 500 and Varian Unity 400 spectrometers) were recorded in $CDCl_3$ or pyridine-d_5 solutions, with tetramethylsilane as internal standard. All measurements were operated at 27 °C. Elemental analyses were performed using a Leco CHNS-932 elemental analyzer.
4-Benzyloxy-2-hydroxybenzonitrile[S2], 4-cyanoresorcinol[S2], 4-butylphenol, 4-hexylphenol, 4-octylphenol, 4-decylphenol, 4-tetradecylpheno, 4-eicosylphenol and 4-docosylphenol were prepared according to the procedures given in ref. [S3]. The synthesis of the final compounds **C/6,6**, [S4] **C/12,12**,[S4] **C/16,16**[S5] and **C/18,18**[S5] is described in the given references. 4-Formyl benzoic acid was used as obtained from Merck. 4-Ethylphenol was used as obtained from *Sigma-Aldrich*.

2.2. Experimental procedures

P1: Esterification. [S6] - To the appropriate benzoic acid **A/*n*** (1 equ), thionyl chloride (5 equ) is added and the mixture is refluxed for 2 hours. Excess thionyl chloride is removed under reduced pressure and the residue is dissolved in dry DCM (1 mL/mmol). After addition of the appropriate alcohol or phenol (1.3 equ), triethylamine (1.3 equ) and pyridine (1 mL) the reaction mixture was refluxed for additional 2 hours. The reaction was monitored by TLC. The mixture was cooled to room temperature quenched with water and the resulting phases were separated. The organic layer was washed with aqu. HCl (10%, 50 ml), aqu. $NaHCO_3$ and brine (50 ml each). The organic layer was dried over Na_2SO_4.The solvent was removed under removed pressure and the obtained crude product was purified by column chromatography.

P2: Oxidation with sodium chlorite[S7]. - The appropriate benzaldehyde **B/*n*** (1 equ) and resorcinol (1.3 equ) were dissolved in *tert*-butanol (5 mL/mmol). An aqueous solution of sodium chlorite (5.8 equ) and potassium dihydrogenphosphate dihydrate (1.3 equ) was added slowly to the reaction mixture und stirred for 1 hour. The solvent was removed under reduced pressure und the obtained residue was suspended in water. Hydrochloric acid (10%) was added to pH = 2-3. The solid was filtered off washed with water and *n*-hexane und crystallized from ethanol.

2.3 Intermediates

2.3.1 4-(4-Alkylphenoxycarbonyl)benzaldehydes (B/*n*)

4-(4-Ethylphenoxycarbonyl)benzaldehyde (B/2): Synthesized according to **P1** from 4-ethylphenol (2.56 g, 0.02 mol), 4-formylbenzoic acid (3.00 g, 0.02 mol), thionyl chloride (30 mL), triethylamine (2.90 mL, 0.03 mol), pyridine (0.08 mL) in DCM (50 mL). Purification by column chromatography (eluent: $CHCl_3$). Colourless solid, $C_{16}H_{14}O_3$, M = 254.28 g/mol, mp. 62 °C, yield: 3.12 g (58%). **^{1}H-NMR** (400 MHz, $CDCl_3$): δ 10.13 (s, 1H, Ar-CHO), 8.34 (d, ^3J = 8.3 Hz, 2H, Ar-H), 8.00 (d, ^3J = 8.3 Hz, 2H, Ar-H), 7.25 (d, ^3J = 7.5 Hz, 2H, Ar-H), 7.12 (d, ^3J = 8.5 Hz, 2H, Ar-H), 2.67 (q, ^3J = 7.6 Hz, 2H, Ar-CH_2), 1.25 (t, ^3J = 7.6 Hz, 3H, CH_3) ppm.

4-(4-Butylphenoxycarbonyl)benzaldehyde (B/4): Synthesized according to **P1** from 4-butylphenol (5.00 g, 33 mmol), 4-formylbenzoic acid (5.25 g, 35 mmol), thionyl chloride (30 mL), triethylamine (3.85 mL, 0.04 mol), pyridine (0.10 mL) in DCM (50 mL). Purification by column chromatography (eluent: $CHCl_3$). Colourless solid, $C_{18}H_{18}O_3$, M = 282.33 g/mol, mp. 86 °C, yield: 4.00 g (43%). **^{1}H-NMR** (400 MHz, $CDCl_3$): δ 10.12 (s, 1H, Ar-CHO), 8.34 (d, ^3J = 8.5 Hz, 2H, Ar-H), 8.00 (d, ^3J = 8.1 Hz, 2H, Ar-H), 7.22 (d, ^3J = 8.2 Hz, 2H, Ar-H), 7.11 (d, ^3J = 8.4 Hz, 2H, Ar-H), 2.67-2.58 (m, 2H, Ar-CH_2), 1.66-1.55 (m, 2H, CH_2), 1.42-1.30 (m, 2H, CH_2), 0.92 (t, ^3J = 7.3 Hz, 3H, CH_3) ppm.

4-(4-Octylphenoxycarbonyl)benzaldehyde (B/8): Synthesized according to **P1** from 4-octylphenol (5.77 g, 28 mmol), 4-formylbenzoic acid (4.00 g, 26 mmol), thionyl chloride (30 mL), triethylamine (4.80 mL, 35 mmol), pyridine (0.10 mL) in DCM (60 mL). Purification by column chromatography (eluent: *n*-hexane/ethyl acetate 95:5). Colourless solid, $C_{22}H_{26}O_3$, M = 338.44 g/mol, mp. 125 °C, yield: 4.20 g (48%). **^{1}H-NMR** (400 MHz, $CDCl_3$): δ 10.13 (s, 1H, Ar-CHO), 8.34 (d, ^3J = 8.3 Hz, 2H, Ar-H), 8.00 (d, ^3J = 8.3 Hz, 2H, Ar-H), 7.22 (d, ^3J = 8.5 Hz, 2H, Ar-H), 7.11 (d, ^3J = 8.5 Hz, 2H, Ar-H), 2.69-2.56 (m, 2H, Ar-CH_2), 1.72-1.57 (m, 2H, CH_2), 1.38-1.18 (m, 10H, CH_2), 0.87 (t, ^3J = 6.8 Hz, 3H, CH_3) ppm.

4-(4-Decylphenoxycarbonyl)benzaldehyde (B/10): Synthesized according to **P1** from 4-decylphenol (6.56 g, 28 mmol), 4-formylbenzoic acid (4.00 g, 26 mmol), triethylamine (4.80 mL, 35 mmol), thionyl chloride (35 mL), pyridine (0.1 mL) in DCM (60 mL). Purification by column chromatography (eluent: *n*-hexane/ethyl acetate 95:5). Colourless solid, $C_{24}H_{30}O_3$, M = 366.49 g/mol, mp. 132 °C, yield: 4.40 g (46%). **^{1}H-NMR** (400 MHz, $CDCl_3$): δ 10.12 (s, 1H, Ar-CHO), 8.34 (d, ^3J = 8.3 Hz, 2H, Ar-H), 8.00 (d, ^3J = 8.3 Hz, 2H, Ar-H), 7.22 (d, ^3J = 8.5 Hz, 2H, Ar-H), 7.11 (d, ^3J = 8.5 Hz, 2H, Ar-H), 2.66-2.56 (m, 2H, Ar-CH_2), 1.64-1.58 (m, 2H, CH_2), 1.35-1.20 (m, 14H, CH_2), 0.87 (t, ^3J = 6.8 Hz, 3H, CH_3) ppm.

4-(4-Tetradecylphenoxycarbonyl)benzaldehyde (B/14): Synthesized according to **P1** from 4-tetradecylphenol (2.28 g, 8.0 mmol), 4-formylbenzoic acid (1.13 g, 8.0 mol), thionyl chloride (15 mL), triethylamine (1.40 mL, 10.0 mmol), pyridine (0.08 mL) in DCM (40 mL) and THF (20 mL). Purification by column chromatography (eluent: $CHCl_3$). Colourless solid, $C_{28}H_{38}O_3$, M = 422.60 g/mol, mp. 145 °C, yield: 1.80 g (53%). **^{1}H-NMR** (400 MHz, $CDCl_3$): δ 10.12 (s, 1H, Ar-CHO), 8.34 (d, ^3J = 8.5 Hz, 2H, Ar-H), 8.00 (d, ^3J = 8.1 Hz, 2H, Ar-H), 7.22 (d, ^3J = 8.3 Hz, 2H, Ar-H), 7.11 (d, ^3J = 8.2 Hz, 2H, Ar-H), 2.65-2.57 (m, 2H, Ar-CH_2), 1.65-1.56 (m, 2H, CH_2), 1.36-1.21 (m, 22H, CH_2), 0.86 (t, ^3J = 6.9 Hz, 3H, CH_3) ppm.

4-(4-Eicosylphenoxycarbonyl)benzaldehyde (B20): Synthesized according to **P1** from 4-eicosylphenol (1.1 g, 2.9 mol), 4-formylbenzoic acid (0.40 g, 2.9 mol), thionyl chloride (20 mL), triethylamine (0.43 mL, 3.1 mmol), pyridine (0.1 mL) in THF (40 mL). Purification by column chromatography (eluent: $CHCl_3$). Colourless solid, $C_{34}H_{50}O_3$, M = 506.76 g/mol, mp. 101-102 °C, yield: 0.64 g (43%). **^{1}H-NMR** (500 MHz, $CDCl_3$): δ 10.15 (s, 1H, Ar-CHO), 8.35 (d, ^3J = 8.1 Hz, 2H, Ar-H), 8.02 (d, ^3J = 8.2 Hz, 2H, Ar-H), 7.20 (d, ^3J = 8.4 Hz, 2H, Ar-H), 7.11 (d, ^3J = 8.5 Hz, 2H, Ar-H), 2.68-2.56 (m, 2H, Ar-CH_2), 1.70-1.57 (m, 2H, CH_2), 1.41-1.15 (m, 34H, CH_2), 0.88 (t, ^3J = 6.5 Hz, 3H, CH_3) ppm.

4-(4-Docosylphenoxycarbonyl)benzaldehyde (B22): Synthesized according to **P1** from 4-docosylphenol (1.05 g, 2.6 mol), 4-formylbenzoic acid (0.36 g, 2.6 mol), thionyl chloride (20 mL), triethylamine (0.43 mL, 3.1 mmol), pyridine (0.1 mL) in THF (40 mL). Purification by column chromatography (eluent: $CHCl_3$). Colourless solid, $C_{36}H_{54}O_3$, M = 534.81 g/mol, mp. 99-100 °C, yield: 0.67 g (48.2%). **^{1}H-NMR** (400 MHz, $CDCl_3$): δ 10.15 (s, 1H, Ar-CHO), 8.36 (d, ^3J = 8.2 Hz, 2H, Ar-H), 8.02 (d, ^3J = 8.1 Hz, 2H, Ar-H), 7.24 (d, ^3J = 8.5 Hz, 2H, Ar-H), 7.13 (d, ^3J = 8.4 Hz, 2H, Ar-H), 2.68-2.56 (m, 2H, Ar-CH_2), 1.70-1.57 (m, 2H, CH_2), 1.43-1.18 (m, 38H, CH_2), 0.88 (t, ^3J = 6.5 Hz, 3H, CH_3) ppm.

2.3.2 4-(4-Alkylphenoxycarbonyl)benzoic acids (A/*n*)

4-(4-Ethylphenoxycarbonyl)benzoic acid (A/2): Synthesized according to **P2** from **B/2** (3.12 g, 12.3 mmol), using resorcinol (1.76 g, 16.0 mmol), sodium chlorite (6.44 g, 71.2 mmol) and sodium dihydrogenphosphate dihydrate (5.74 g, 37.0 mmol) in *t*-BuOH (120 mL) and water (70 mL). Purification by crystallization from EtOH. Colourless solid, $C_{16}H_{14}O_4$, M = 270.28 g/mol, mp. 245 °C, yield: 2.88 g (87%). **^{1}H-NMR** (400 MHz, DMSO-d$_6$): δ 8.19 (d, ^3J = 8.3 Hz, 2H, Ar-H), 8.09 (d, ^3J = 8.4 Hz, 2H, Ar-H), 7.27 (d, ^3J = 8.5 Hz, 2H, Ar-H), 7.17 (d, ^3J = 8.5 Hz, 2H, Ar-H), 2.61 (q, ^3J = 7.6 Hz, 2H, Ar-CH_2), 1.17 (t, ^3J = 7.6 Hz, 3H, CH_3) ppm.

4-(4-Butylphenoxycarbonyl)benzoic acid (A/4): Synthesized according to **P2** from **B/4** (4.00 g, 18.0 mmol), using resorcinol (2.03 g, 18.0 mmol), sodium chlorite (7.43 g, 82.0 mmol) and sodium dihydrogenphosphate dihydrate (6.63 g, 43.0 mmol) in *t*-BuOH (150 mL) and water (80 mL). Purification by crystallization from EtOH. Colourless solid, $C_{18}H_{18}O_4$, M = 298.33 g/mol, mp. 251 °C, yield: 3.50 g (83%). **^{1}H-NMR** (400 MHz, DMSO-d$_6$): δ 8.21 (d, ^3J = 8.5 Hz, 2H, Ar-H), 8.11 (d, ^3J = 8.5 Hz, 2H, Ar-H), 7.27 (d, ^3J = 8.5 Hz, 2H, Ar-H), 7.18 (d, ^3J = 8.5 Hz, 2H, Ar-H), 2.64-2.56 (m, 2H, Ar-CH_2), 1.63-1.50 (m, 2H, CH_2), 1.37-1.24 (m, 2H, CH_2), 0.89 (t, ^3J = 7.3 Hz, 3H, CH_3) ppm.

4-(4-Octylphenoxycarbonyl)benzoic acid (A/8): Synthesized according to **P2** from **B/5** (4.20 g, 12.4 mmol), using resorcinol (1.80 g, 16.0 mmol), sodium chlorite (6.50 g, 72.0 mmol) and sodium dihydrogenphosphate dihydrate (5.80 g, 37.0 mmol) in *t*-BuOH (150 mL) and water (70 mL). Purification by crystallization from EtOH. Colourless solid,

$C_{22}H_{26}O_4$, M = 354.44 g/mol, mp. 223 °C, yield: 3.30 g (74%). **^{1}H-NMR** (400 MHz, DMSO-d$_6$): δ 8.21 (d, ^3J = 8.1 Hz, 2H, Ar-H), 8.11 (d, ^3J = 8.2 Hz, 2H, Ar-H), 7.26 (d, ^3J = 7.8 Hz, 2H, Ar-H), 7.18 (d, ^3J = 7.9 Hz, 2H, Ar-H), 2.58 (t, ^3J = 7.5 Hz, 2H, Ar-CH_2), 1.62-1.51 (m, 2H, CH_2), 1.34-1.17 (m, 10H, CH_2), 0.84 (t, ^3J = 6.2 Hz, 3H, CH_3) ppm.

4-(4-Decylphenoxycarbonyl)benzoic acid (A/10): Synthesized according to **P2** from **B/10** (4.40 g, 11.5 mmol), using resorcinol (1.64 g, 15.0 mmol), sodium chlorite (6.00 g, 70.0 mmol) and sodium dihydrogenphosphate dihydrate (5.40 g, 35.0 mmol) in *t*-BuOH (160 mL) and water (75 mL). Purification by crystallization from EtOH. Colourless solid, $C_{24}H_{30}O_4$, M = 382.49 g/mol, mp. 221 °C, yield: 3.20 g (72%). **^{1}H-NMR** (400 MHz, DMSO-d$_6$): δ 8.27 (d, ^3J = 8.3 Hz, 2H, Ar-H), 8.18 (d, ^3J = 8.4 Hz, 2H, Ar-H), 7.34 (d, ^3J = 8.3 Hz, 2H, Ar-H), 7.26 (d, ^3J = 8.4 Hz, 2H, Ar-H), 2.66 (t, ^3J = 7.6 Hz, 2H, Ar-CH_2), 1.71-1.60 (m, 2H, CH_2), 1.41-1.24 (m, 14H, CH_2), 0.92 (t, ^3J = 6.6 Hz, 3H, CH_3) ppm.

4-(4-Tetradecylphenoxycarbonyl)benzoic acid (A/14): Synthesized according to **P2** from **B/14** (1.80 g, 4.3 mmol), using resorcinol (0.61 g, 5.5 mmol), sodium chlorite (2.23 g, 25.0 mmol) and sodium dihydrogenphosphate dihydrate (2.00 g, 13.0 mmol) in *t*-BuOH (60 mL) and water (25 mL). Purification by crystallization from EtOH. Colourless solid, $C_{28}H_{38}O_4$, M = 438.60 g/mol, mp. 218 °C, yield: 1.73 g (93%). **^{1}H-NMR** (400 MHz, DMSO-d$_6$): δ 8.10 (d, ^3J = 8.3 Hz, 2H, Ar-H), 8.05 (d, ^3J = 8.3 Hz, 2H, Ar-H), 7.25 (d, ^3J = 8.4 Hz, 2H, Ar-H), 7.16 (d, ^3J = 8.5 Hz, 2H, Ar-H), 2.60 (t, ^3J = 7.6 Hz, 2H, Ar-CH_2), 1.65-1.53 (m, 2H, CH_2), 1.35-1.16 (m, 22H, CH_2), 0.85 (t, ^3J = 6.7 Hz, 3H, CH_3) ppm.

4-(4-Eicosylphenoxycarbonyl)benzoic acid (A/20): Synthesized according to **P2** from **B/20** (0.64 g, 1.26 mmol), using resorcinol (0.16 g, 1.75 mmol), sodium chlorite (0.7 g, 7.5 mmol) and sodium dihydrogenphosphate dihydrate (0.62 g, 4.0 mmol) in *t*-BuOH (50 mL) and water (20 mL). Used for the next step without further purification. White solid, $C_{34}H_{50}O_4$, M = 522.76 g/mol, yield: 0.63 g (98.43%). **^{1}H-NMR** (500 MHz, DMSO-d$_6$): δ 8.22 (d, ^3J = 8.4 Hz, 2H, Ar-H), 8.09 (d, ^3J = 8.4 Hz, 2H, Ar-H), 7.25 (d, ^3J = 8.5 Hz, 2H, Ar-H), 7.19 (d, ^3J = 8.5 Hz, 2H, Ar-H), 2.62-2.53 (m, 2H, Ar-CH_2), 1.63-1.52 (m, 2H, CH_2), 1.22-1.16 (m, 34H, CH_2), 0.83 (t, ^3J = 6.8 Hz, 3H, CH_3) ppm.

4-(4-Docosylphenoxycarbonyl)benzoic acid (A22): Synthesized according to **P2** from **B/22** (0.94 g, 1.7 mmol), using resorcinol (0.25 g, 2.3 mmol), sodium chlorite (0.92 g, 9.8 mmol) and sodium dihydrogenphosphate dihydrate (0.81 g, 5.3 mmol) in *t*-BuOH (50 mL) and water (20 mL). Used for the next step without further purification. White solid, $C_{36}H_{54}O_4$, M = 550.81 g/mol, yield: 0.93 g (98.93%). **^{1}H-NMR** (400 MHz, DMSO-d$_6$): δ 8.18 (d, ^3J = 8.4 Hz, 2H, Ar-H), 8.07 (d, ^3J = 8.4 Hz, 2H, Ar-H), 7.26 (d, ^3J = 8.5 Hz, 2H, Ar-H), 7.15 (d, ^3J = 8.5 Hz, 2H, Ar-H), 2.61-2.55 (m, 2H, Ar-CH_2), 1.63-1.52 (m, 2H, CH_2), 1.22-1.16 (m, 38H, CH_2), 0.83 (t, ^3J = 6.8 Hz, 3H, CH_3) ppm.

2.4 4-Cyano-1,3-phenylen bis[4-(4-alkylphenoxycarbonyl)benzoates] (1/*n*)

4-Cyano-1,3-phenylen bis[4-(4-ethylphenoxycarbonyl)benzoate] (1/2). Synthesized according to P1 from A/2 (135 mg, 0.5 mmol), 4-cyanoresorcinol (37 mg, 0.2 mmol), thionyl chloride (25 mL), triethylamine (0.09 mL, 0.6 mmol), DMAP (5 mg) in DCM (30 mL). Purification by column chromatography (eluent: $CHCl_3$) and crystallization from EtOH/$CHCl_3$ 9:1. Colourless solid, $C_{39}H_{29}NO_8$, M = 639.65 g/mol, yield: 131 mg (82%). ^{1}H-NMR (500 MHz, $CDCl_3$): δ 8.39-8.28 (m, 8H, Ar-H), 7.82 (d, ^3J = 8.6 Hz, 1H, Ar-H), 7.59 (d, ^4J = 2.1 Hz, 1H, Ar-H), 7.36 (dd, ^3J = 8.5 Hz, ^4J = 2.2 Hz, 1H, Ar-H), 7.27-7.22 (m, 4H, Ar-H), 7.14 (d, ^3J = 8.6 Hz, 2H, Ar-H), 7.13 (d, ^3J = 8.6 Hz, 2H, Ar-H), 2.68 (q, ^3J = 7.6 Hz,

4H, Ar-CH$_2$), 1.25 (t, ^3J = 7.6 Hz, 6H, CH$_3$) ppm. ^{13}C-NMR (126 MHz, CDCl$_3$): δ 164.20, 163.11, 162.79, 154.54, 153.22, 148.56, 148.53, 142.23, 142.18, 134.86, 134.74, 134.10, 132.53, 132.13, 130.61, 130.47, 130.41, 130.39, 128.90, 128.88, 121.18, 121.15, 120.12, 117.24, 114.52, 104.49, 28.29, 15.52 ppm. EA: calc. for $C_{39}H_{29}NO_8$: C 73.23, H 4.57, N 2.19; found C 72.87, H 4.43, N 2.36.

4-Cyano-1,3-phenylen bis[4-(4-butylphenoxycarbonyl)benzoate] (1/4). Synthesized according to **P1** from **A/4** (401 mg, 1.4 mmol), 4-cyanoresorcinol (100 mg, 0.7 mmol), thionyl chloride (25 mL), triethylamine (0.25 mL, 1.7 mmol), pyridine (0.1 mL) in DCM (35 mL). Purification by column chromatography (eluent: CHCl$_3$) and crystallization from EtOH/CHCl$_3$ 9:1. Colourless solid, $C_{43}H_{37}NO_8$, M = 695.76 g/mol, yield: 301 mg (64%). **^{1}H-NMR** (500 MHz, CDCl$_3$): δ 8.34-8.22 (m, 8H, Ar-H), 7.76 (d, ^3J = 8.5 Hz, 1H, Ar-H), 7.53 (d, ^4J = 2.1 Hz, 1H, Ar-H), 7.30 (dd, ^3J = 8.5 Hz, ^4J = 2.2 Hz, 1H, Ar-H), 7.21-7.14 (m, 4H, Ar-H), 7.07 (d, ^3J = 8.6 Hz, 2H, Ar-H), 7.07 (d, ^3J = 8.6 Hz, 2H, Ar-H), 2.61-2.53 (m, 4H, Ar-CH$_2$), 1.59-1.51 (m, 4H, CH$_2$), 1.35-1.26 (m, 4H, CH$_2$), 0.87 (t, ^3J = 7.4 Hz, 6H, CH$_3$) ppm. **^{13}C-NMR** (126 MHz, CDCl$_3$): δ 164.18, 163.11, 162.78, 154.54, 153.22, 148.56, 148.53, 140.92, 140.87, 134.87, 134.75, 134.10, 132.53, 132.13, 130.61, 130.46, 130.40, 130.40, 129.43, 129.41, 121.10, 121.06, 120.12, 117.24, 114.52, 104.49, 35.04, 33.56, 22.28, 13.90, 13.57 ppm. **EA**: calc. for $C_{43}H_{37}NO_8$: C 74.23, H 5.36, N 2.01; found C 74.15, H 5.33, N 2.17.

4-Cyano-1,3-phenylen bis[4-(4-octylphenoxycarbonyl)benzoate] (1/8). Synthesized according to **P1** from **A/8** (213 mg, 0.6 mmol), 4-cyanoresorcinol (45 mg, 0.3 mmol), thionyl chloride (20 mL), triethylamine (0.11 mL, 0.8 mmol), pyridine (0.05 mL) in DCM (30 mL). Purification by column chromatography (eluent: CHCl$_3$) and crystallization from EtOH/CHCl$_3$ 9:1. Colourless solid, $C_{51}H_{53}NO_8$, M = 807.97 g/mol, yield: 165 mg (68%). **^{1}H-NMR** (500 MHz, CDCl$_3$): δ 8.42-8.28 (m, 8H, Ar-H), 7.84 (d, ^3J = 8.5 Hz, 1H, Ar-H), 7.61 (d, ^4J = 2.1 Hz, 1H, Ar-H), 7.38 (dd, ^3J = 8.5 Hz, ^4J = 2.2 Hz, 1H, Ar-H), 7.28-7.21 (m, 4H, Ar-H), 7.15 (d, ^3J = 8.5 Hz, 2H, Ar-H), 7.14 (d, ^3J = 8.5 Hz, 2H, Ar-H), 2.68-2.60 (m, 4H, Ar-CH$_2$), 1.68-1.59 (m, 4H, CH$_2$), 1.40-1.20 (m, 10H, CH$_2$), 0.89 (t, ^3J = 6.9 Hz, 6H, CH$_3$) ppm. **^{13}C-NMR** (101 MHz, CDCl$_3$): δ 164.08, 163.04, 162.72, 154.52, 153.21, 148.56, 148.53, 140.94, 140.88, 134.90, 134.78, 134.06, 132.55, 132.15, 130.60, 130.44, 130.38, 129.39, 129.37, 121.09, 121.06, 120.09, 117.23, 114.51, 104.56, 35.53, 32.02, 31.58, 29.60, 29.43, 29.39, 22.81, 14.24 ppm. **EA**: calc. for $C_{51}H_{53}NO_8$: C 75.81, H 6.61, N 1.73; found C 75.91, H 6.28, N 1.68.

4-Cyano-1,3-phenylen bis[4-(4-decylphenoxycarbonyl)benzoate] (1/10). Synthesized according to **P1** from **A/10** (230 mg, 0.6 mmol), 4-cyanoresorcinol (45 mg, 0.3 mmol), thionyl chloride (20 mL), triethylamine (0.11 mL, 0.8 mmol), pyridine (0.05 mL) in DCM (30 mL). Purification by column chromatography (eluent: CHCl$_3$) and crystallization from EtOH/CHCl$_3$ 9:1. Colourless solid, $C_{55}H_{61}NO_8$, M = 864.1 g/mol, yield: 218 mg (84%). **^{1}H-NMR** (500 MHz, CDCl$_3$): δ 8.42-8.27 (m, 8H, Ar-H), 7.84 (d, ^3J = 8.6 Hz, 1H, Ar-H), 7.61 (d, ^4J = 2.1 Hz, 1H, Ar-H), 7.38 (dd, ^3J = 8.5 Hz, ^4J = 2.2 Hz, 1H, Ar-H), 7.27-7.22 (m, 4H, Ar-H), 7.15 (d, ^3J = 8.5 Hz, 2H, Ar-H), 7.14 (d, ^3J = 8.5 Hz, 2H, Ar-H), 2.68-2.59 (m, 4H, Ar-CH$_2$), 1.67-1.60 (m, 4H, CH$_2$), 1.39-1.21 (m, 32H, CH$_2$), 0.89 (t, ^3J = 6.9 Hz, 6H, CH$_3$) ppm. **^{13}C-NMR** (101 MHz, CDCl$_3$): δ 164.08, 163.04, 162.71, 154.52, 153.21, 148.56, 148.53, 140.94, 140.89, 134.90, 134.78, 134.06, 132.55, 132.16, 130.60, 130.45, 130.38, 129.39, 129.38, 121.09, 121.06, 120.10, 117.24, 114.51, 104.56, 35.54, 32.04, 31.58, 29.76, 29.74, 29.64, 29.47, 29.43, 22.83, 14.26 ppm. **EA**: calc. for $C_{55}H_{61}NO_8$: C 76.45, H 7.11, N 1.62; found C 76.28, H 6.88, N 1.58.

4-Cyano-1,3-phenylen bis[4-(4-tetradecylphenoxycarbonyl)benzoate] (1/14). Synthesized according to **P1** from **A/14** (400 mg, 0.9 mmol), 4-cyanoresorcinol (68 mg, 0.5 mmol), thionyl chloride (25 mL), triethylamine (0.17 mL, 1.2 mmol), pyridine (0.05 mL) in DCM (30 mL). Purification by column chromatography (eluent: $CHCl_3$) and crystallization from EtOH/$CHCl_3$ 9:1. Colourless solid, $C_{63}H_{77}NO_8$, M = 976.29 g/mol, yield: 366 mg (75%). **^{1}H-NMR** (500 MHz, $CDCl_3$): δ 8.40-8.30 (m, 8H, Ar-H), 7.84 (d, ^3J = 8.6 Hz, 1H, Ar-H), 7.60 (d, ^4J = 2.1 Hz, 1H, Ar-H), 7.38 (dd, ^3J = 8.5 Hz, ^4J = 2.2 Hz, 1H, Ar-H), 7.27-7.21 (m, 4H, Ar-H), 7.15 (d, ^3J = 8.6 Hz, 2H, Ar-H), 7.14 (d, ^3J = 8.5 Hz, 2H, Ar-H), 2.66-2.60 (m, 4H, Ar-CH_2), 1.66-1.60 (m, 4H, CH_2), 1.37-1.21 (m, 44H, CH_2), 0.88 (t, ^3J = 6.9 Hz, 6H, CH_3) ppm. **^{13}C-NMR** (126 MHz, $CDCl_3$): δ 164.17, 163.13, 162.78, 154.54, 153.22, 148.54, 140.93, 134.87, 134.75, 134.09, 132.59, 132.07, 130.61, 130.46, 130.39, 129.41, 129.40, 121.08, 121.05, 120.18, 117.24, 114.46, 104.49, 35.37, 31.89, 31.43, 29.66, 29.64, 29.56, 29.47, 29.32, 29.26, 22.66, 14.08 ppm. **EA**: calc. for $C_{63}H_{77}NO_8$: C 77.51, H 7.95, N 1.43; found C 77.32, H 7.72, N 1.45.

4-Cyano-1,3-phenylen bis[4-(4-eicosylphenoxycarbonyl)benzoate] (1/20). Synthesized according to **P1** from **A/20** (320 mg, 0.6 mmol), 4-cyanoresorcinol (40 mg, 0.3 mmol), thionyl chloride (25 mL), triethylamine (0.11 mL, 0.8 mmol), pyridine (0.05 mL) in DCM (30 mL). Purification by column chromatography (eluent: $CHCl_3$) and crystallization from EtOH/$CHCl_3$ 9:1. Colourless solid, $C_{75}H_{101}NO_8$, M = 1144.61 g/mol, yield: 230 mg (71.9%). **^{1}H-NMR** (500 MHz, $CDCl_3$): δ 8.45-8.26 (m, 8H, Ar-H), 7.84 (d, ^3J = 8.5 Hz, 1H, Ar-H), 7.61 (d, ^4J = 2.2 Hz, 1H, Ar-H), 7.38 (dd, ^3J = 8.6 Hz, ^4J = 2.2 Hz, 1H, Ar-H), 7.25 (m, 4H, Ar-H), 7.19-7.10 (m, 4H, Ar-H), 2.71-2.52 (m, 4H, Ar-CH_2), 1.72-1.56 (m, 4H, CH_2), 1.45-1.15 (m, 64H, CH_2), 0.88 (t, ^3J = 6.8 Hz, 6H, CH_3) ppm. **^{13}C-NMR** (101 MHz, $CDCl_3$): δ 164.18, 163.12, 162.79, 154.54, 153.23, 148.55, 148.52, 140.99, 134.88, 134.76, 134.10, 132.53, 132.14, 130.62, 130.47, 130.40, 129.42, 129.41, 121.10, 121.06, 120.13, 117.25, 114.46, 104.50, 35.38, 31.90, 31.45, 29.68, 29.64, 29.58, 29.48, 29.34, 29.28, 22.67, 14.09 ppm. **EA**: calc. for $C_{75}H_{101}NO_8$: C 78.70, H 8.89, N 1.22; found C 78.61, H 8.80, N 1.18.

4-Cyano-1,3-phenylen bis[4-(4-docosylphenoxycarbonyl)benzoate] (1/22). Synthesized according to **P1** from **A/22** (310 mg, 0.6 mmol), 4-cyanoresorcinol (40 mg, 0.3 mmol), thionyl chloride (25 mL), triethylamine (0.11 mL, 0.6 mmol), pyridine (0.05 mL) in DCM (30 mL). Purification by column chromatography (eluent: $CHCl_3$) and crystallization from EtOH/$CHCl_3$ 9:1. Colourless solid, $C_{79}H_{109}NO_8$, M = 1200.71 g/mol, yield: 250 mg (80.6%). **^{1}H-NMR** (400 MHz, $CDCl_3$): δ 8.47-8.25 (m, 8H, Ar-H), 7.84 (d, ^3J = 8.6 Hz, 1H, Ar-H), 7.60 (d, ^4J = 2.2 Hz, 1H, Ar-H), 7.38 (dd, ^3J = 8.6 Hz, ^4J = 2.2 Hz, 1H, Ar-H), 7.30-7.20 (m, 4H, Ar-H), 7.18-7.07 (m, 4H, Ar-H), 2.71-2.54 (m, 4H, Ar-CH_2), 1.73-1.57 (m, 4H, CH_2), 1.47-1.13 (m, 56H, CH_2), 0.88 (t, ^3J = 6.9 Hz, 6H, CH_3) ppm. **^{13}C-NMR** (101 MHz, $CDCl_3$): δ 164.19, 163.13, 162.79, 154.54, 153.23, 148.55, 148.52, 140.99, 134.88, 134.76, 134.10, 132.53, 132.14, 130.62, 130.47, 130.40, 129.42, 129.41, 121.09, 121.06, 120.13, 117.25, 114.46, 104.50, 35.38, 31.90, 31.45, 29.67, 29.64, 29.58, 29.48, 29.33, 29.28, 22.66, 14.09 ppm. **EA**: calc. for $C_{79}H_{109}NO_8$: C 79.02, H 9.15, N 1.17; found C 78.93, H 9.00, N 1.15.

3. Additional Data

3.1 Phase transitions

Table S1. Phase transitions of compounds **1/*n*** on cooling.[a]

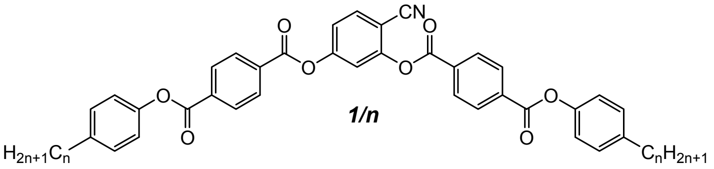

**1/*n***	*T*/ °C [Δ*H*/ kJ·mol^{-1}][a]
**1/2**	Cr <40 N_{cybA} 117 [-0.3]) Iso←
**1/4**	Cr <40 SmC_aP_A 89 [-1.5] SmA 126 [-1.7] N_{CybA} 128 [-0.8]) Iso←
**1/6**	Cr <40 SmC_aP_A 112 [-1.1]) $SmAP_{AR}$ ~117 SmA 148 [-5.3] Iso←
**1/8**	Cr <40 SmC_aP_A 116 [-0.6]) $SmAP_{AR}$ ~117 SmA 158 [-6.8] Iso←
**1/10**	Cr <40 SmC_aP_A 114 [-0.5]) $SmAP_R$/SmA 163 [-7.8] Iso←
**1/12**	Cr 37 [-11.2] SmC_aP_A 101 [-] **Sm(CP)^{hel}** 110 [-] SmC_aP_A 112 [-0.8]) $SmAP_R$/SmA 165 [-8.0] Iso←
**1/14**	Cr 54 [-39.0] SmC_aP_A 82 [-] $SmC_aP_A{}^{(hel)}$ 91 [-] **Sm(CP)^{hel}** 110 [-1.0]) $SmAP_R$/SmA 163 [-8.0] Iso←
**1/16**	Cr 61 -[38.8][b] SmA'P_F 82 [-] SmC_aP_A 92 [-] **Sm(CP)^{hel}** 108 [-0.8]) SmC_xP_R 125 [-] $SmAP_R$/SmA 159 [-7.9] Iso←
**1/18**	Cr 71 [-54.2][b] SmA'P_F 85 [-] SmC_sP_A 107 [-0.9] $SmC_sP_R{}^{[*]}$ 134 [-] $SmAP_R$/SmA 157 [-7.4] Iso←
**1/20**	Cr' 82 [-61.2] SmA'P_F 86 [-] SmC_sP_A 106 [-1.4] SmC_sP_{AR} 111 [-] SmC_s 129 [-] SmA 154 [-6.4] Iso←
**1/22**	Cr' 87 [-56.6] SmC_sP_A 102 [-1.4] SmC_sP_{AR} 104 [-] SmC_s 128 SmA 149 [-5.8] Iso←

[a] For abbreviations, see Table 1. The development of the phase assignment in the progress of understanding the behavior of compounds **1/*n*** and the designation of the LC phases is documented in Table S2. The data in this table refer to the peak temperatures in the DSC cooling traces at a rate of 10 K min^{-1} for transitions associated with enthalpies (and from optical/electro-optical investigations in thin films between microscopy glass plates for transitions without enthalpy) and therefore can slightly deviate (by 1-2K) from the temperatures given in previous reports (Refs. S4, S5 and S8-S23) due to different conditions; for crystallization temperatures even larger differences can be observed, due to different confinement conditions and surface effects. [b] a crystallization temperature of 77 and 76 °C was observed in electro-optical cells for compounds **1/16** and **1/18**, respectively.[S5,S19-S23]

3.2 Compound 1/6

a) b)

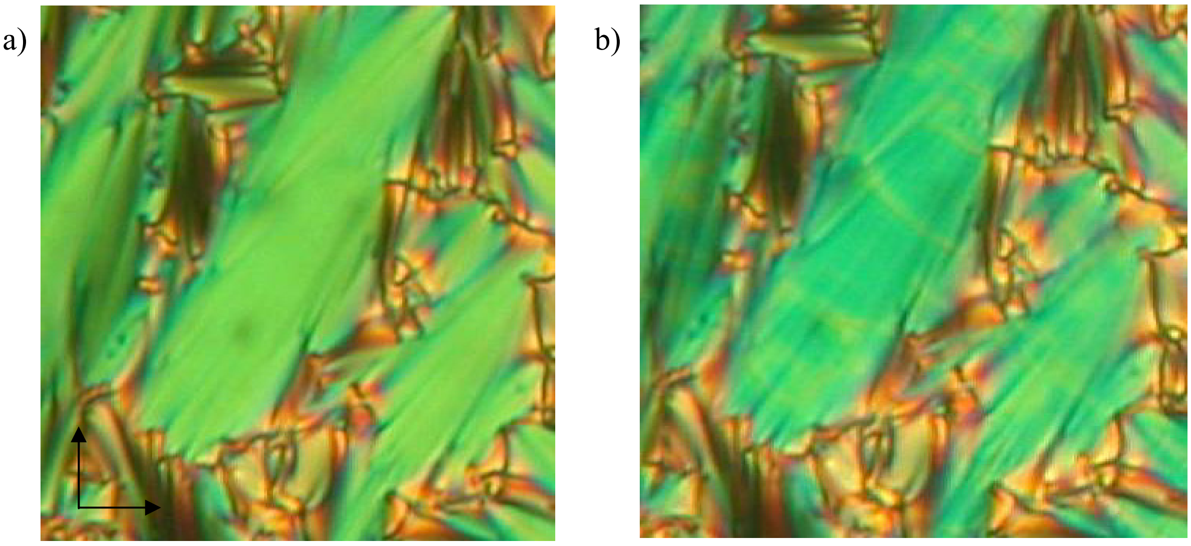

Figure S1. Planar textures of **1/6** a) at T = 113 °C in the SmA phase and b) at T = 103 °C in the SmC_aP_A phase with speckled texture.

a) b)

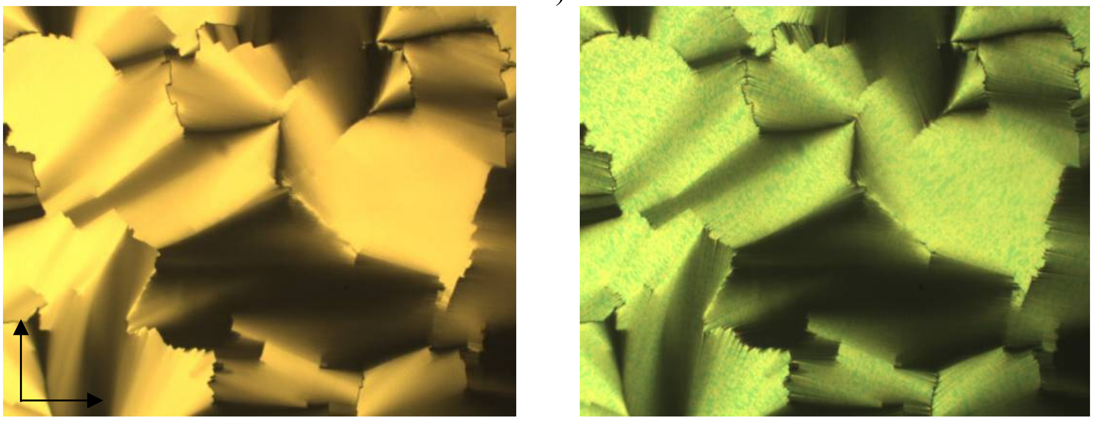

Figure S2. Planar textures of **1/6** in the SmC_aP_A phase at T = 90 °C, a) smooth texture under an applied DC field (15 Vpp, in a 6 μm ITO cell) and b) speckled texture after switching off the applied field at 0V.

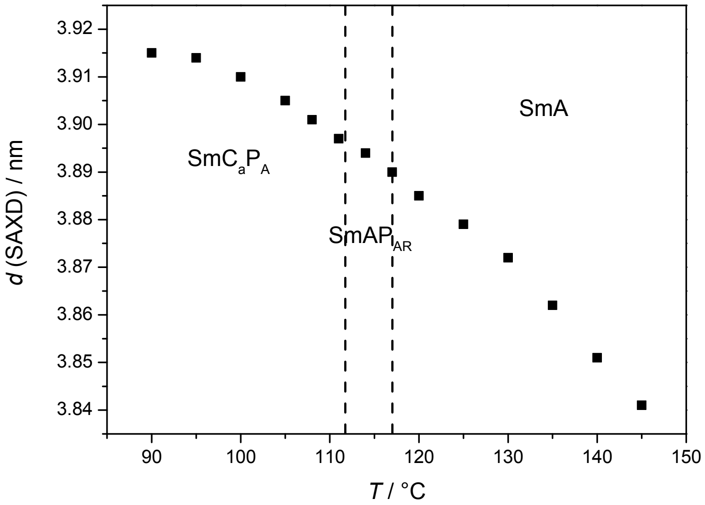

Figure S3. Temperature dependence of the *d*-values of compound **1/6**.

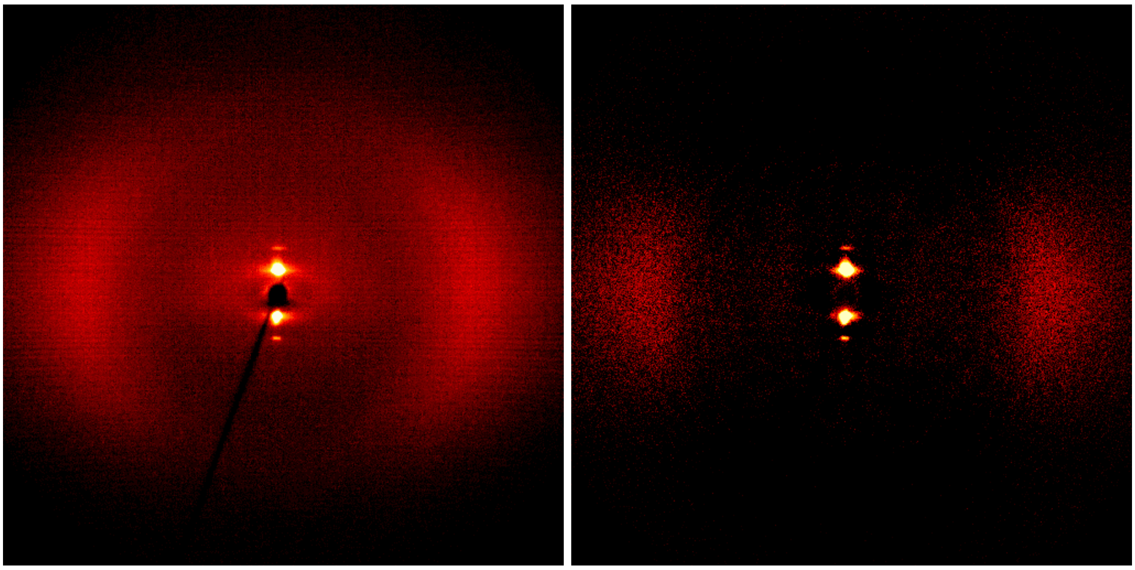

a) SmA at 130 °C

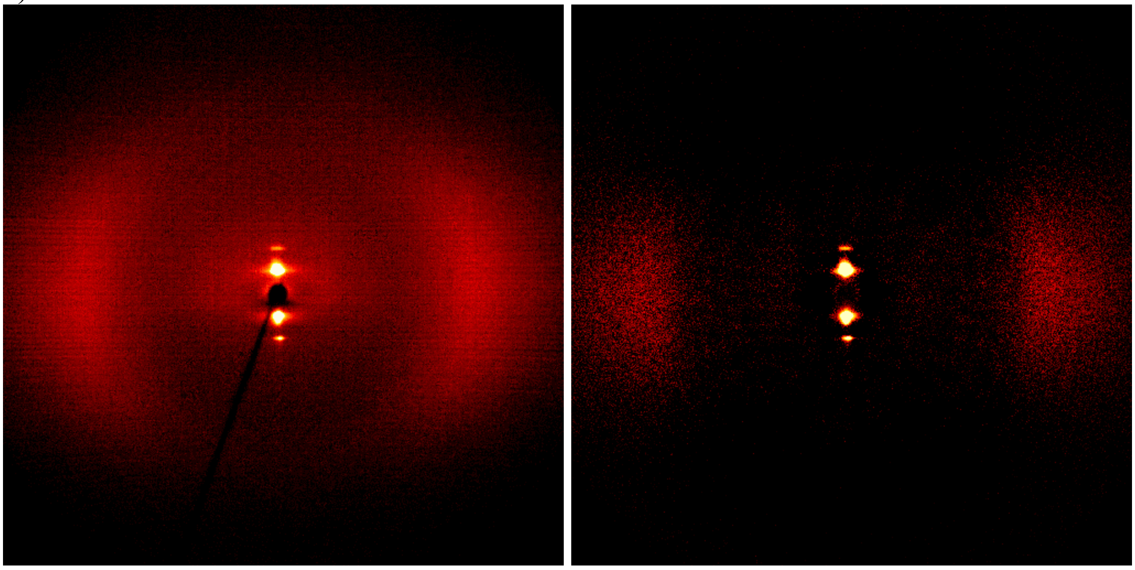

b) $SmAP_{AR}$ at 110 °C

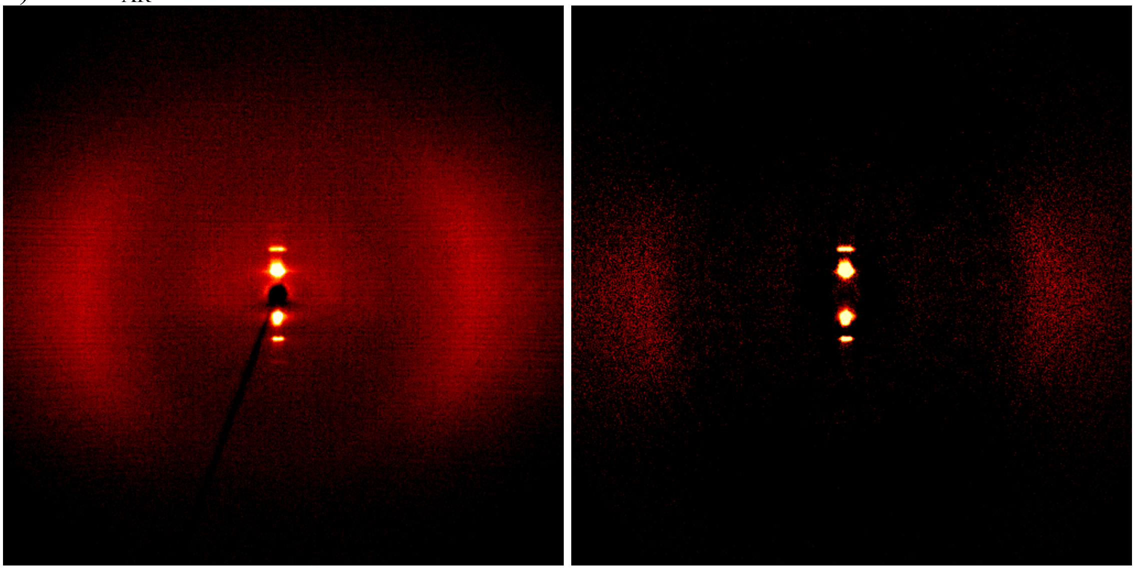

c) SmC_aP_A at 90 °C

Figure S4. XRD pattern of a surface aligned sample of **1/6** at the given temperatures, left original patterns, right patterns after subtraction of the isotropic pattern.

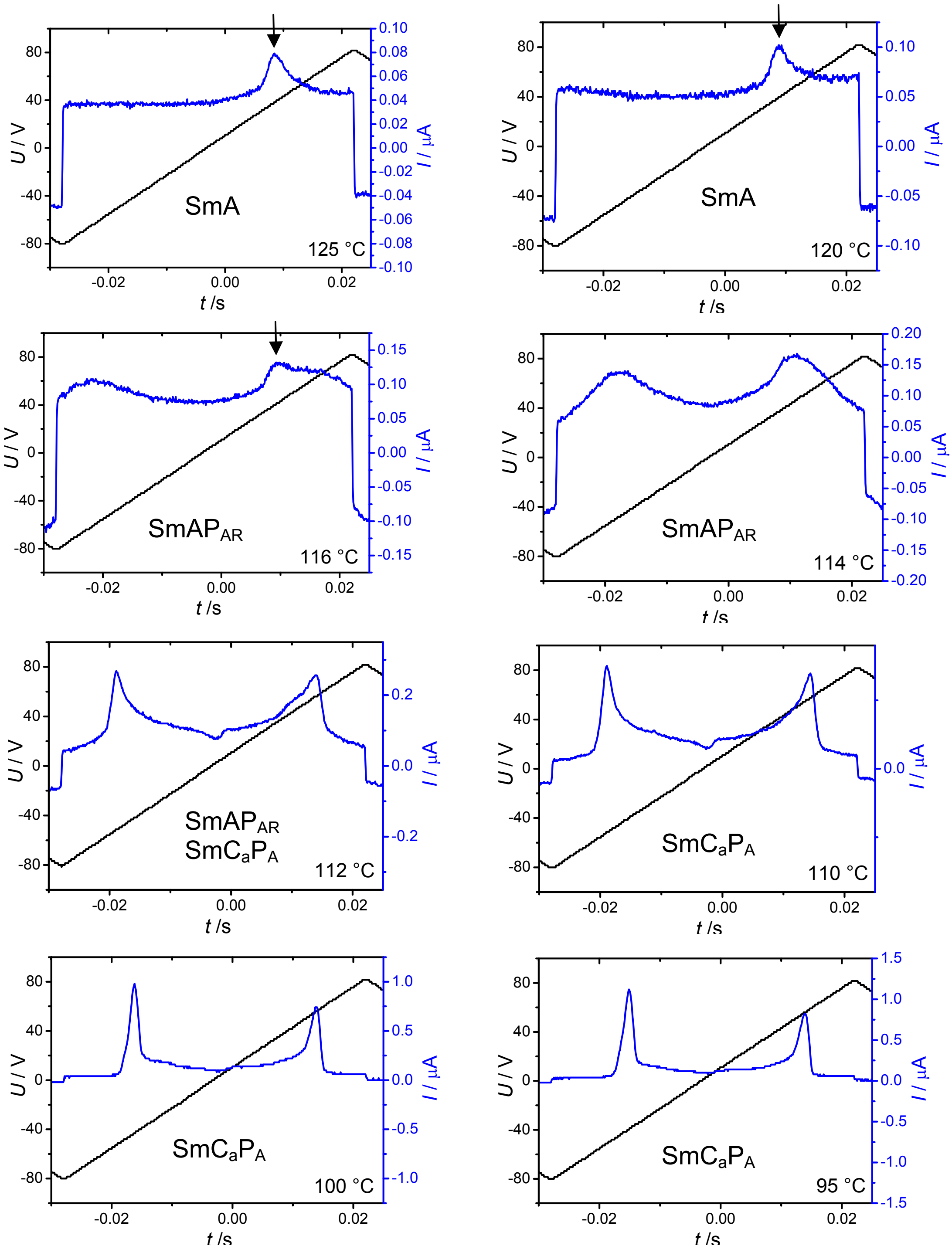

Figure S5. Development of the polarization current peaks of **1/6** depending on temperature; the sharp single peak indicated with an arrow is attributed to conductivity.

3.3 Compound 1/8

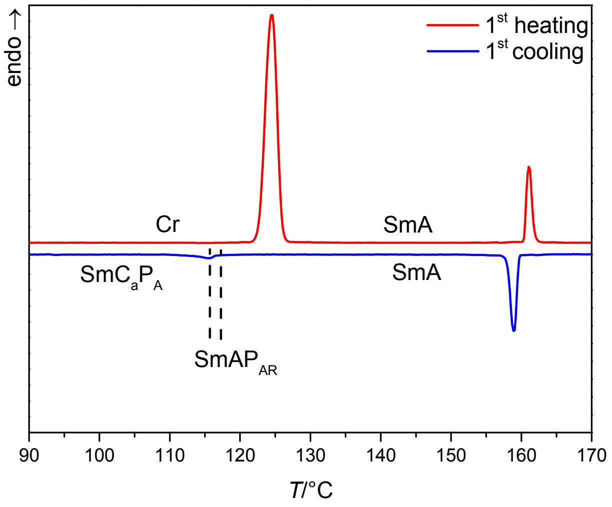

Figure S6. DSC traces of compound **1/8** (10 K min-1).

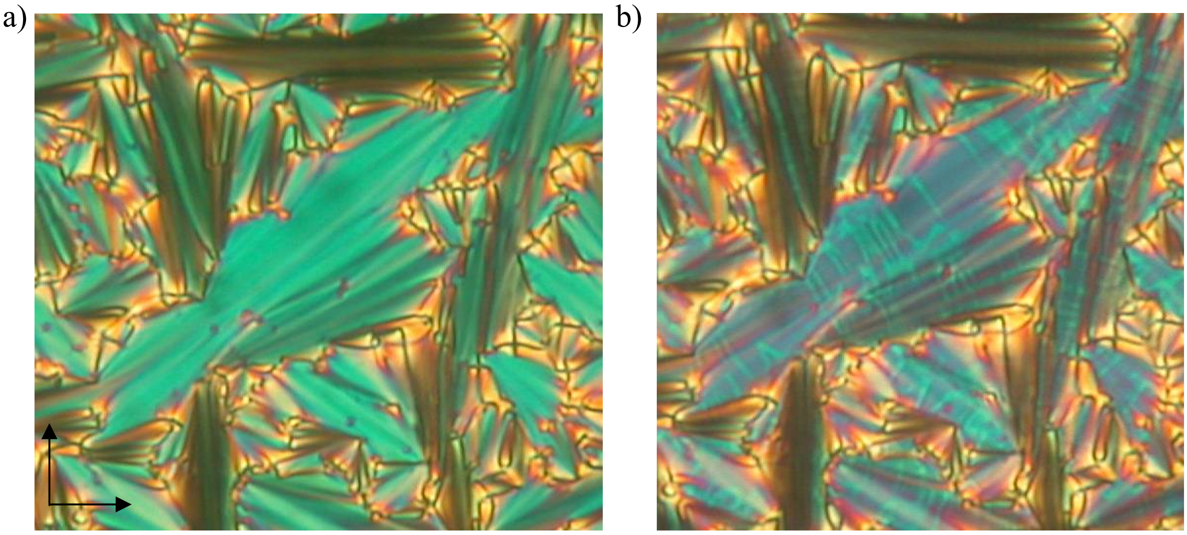

Figure S7. Planar textures of **1/8** a) at T = 120 °C in the SmA phase and b) at T = 102 °C in the SmC_aP_A phase with speckled texture.

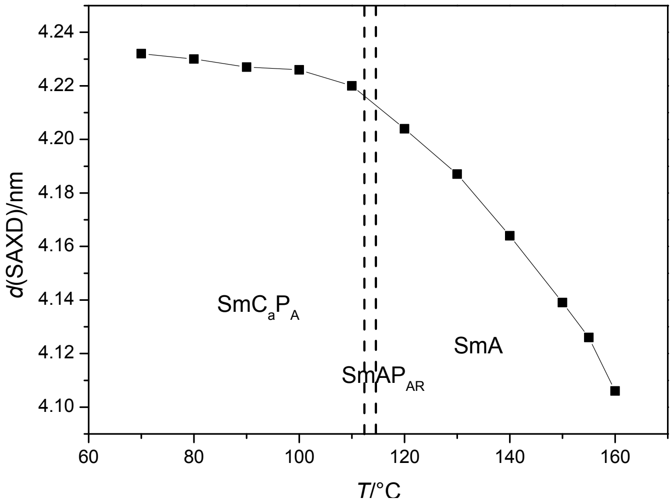

Figure S8. Temperature dependence of the d-values of compound **1/8**.

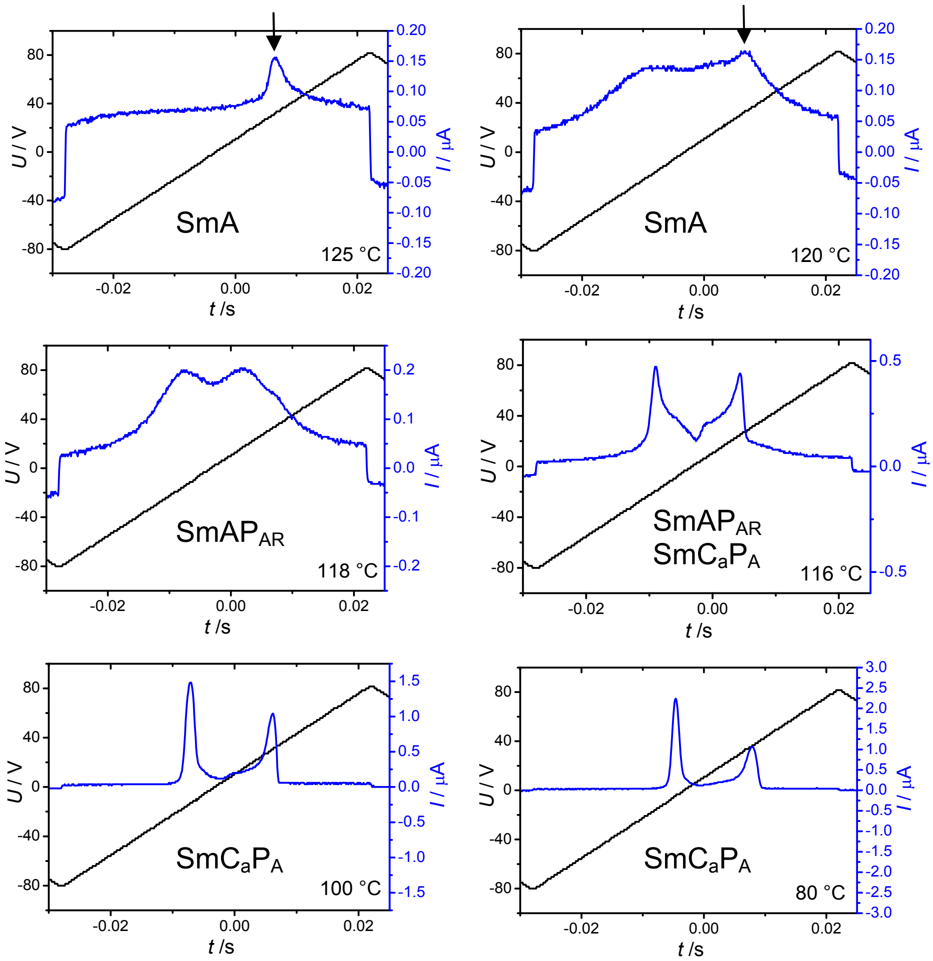

Figure S9. Development of the polarization current peaks of **1/8** depending on temperature; the sharp single peak indicated with an arrow is attributed to conductivity.

3.4 Compound 1/10

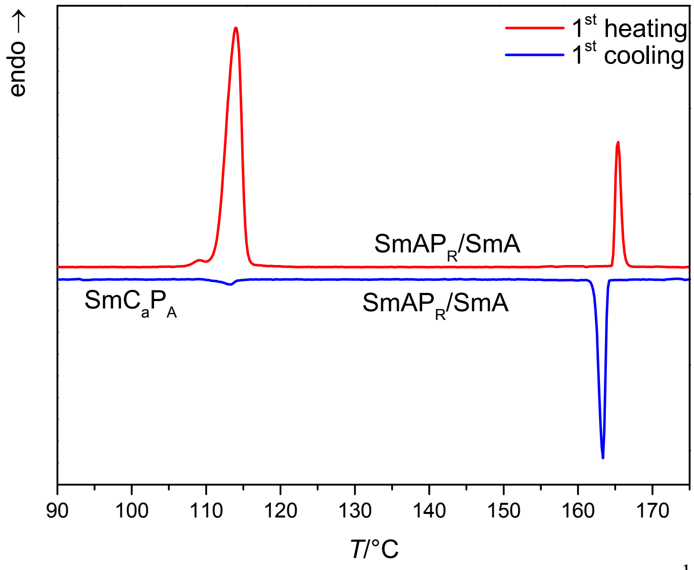

Figure S10. DSC traces of compound **1/10** (10 K min^{-1}).

a) c) b) d)

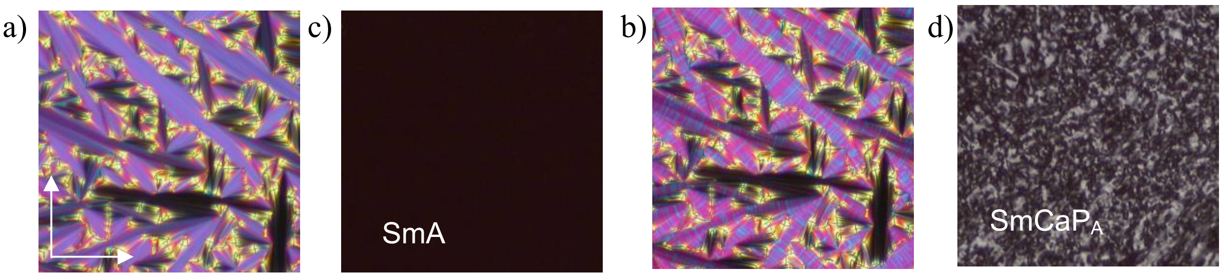

Figure S11. a,c) Planar textures and b,d) homeotropic textures of **1/10** a) at a,b) $T = 120$ °C and c,d) at $T = 110$ °C.

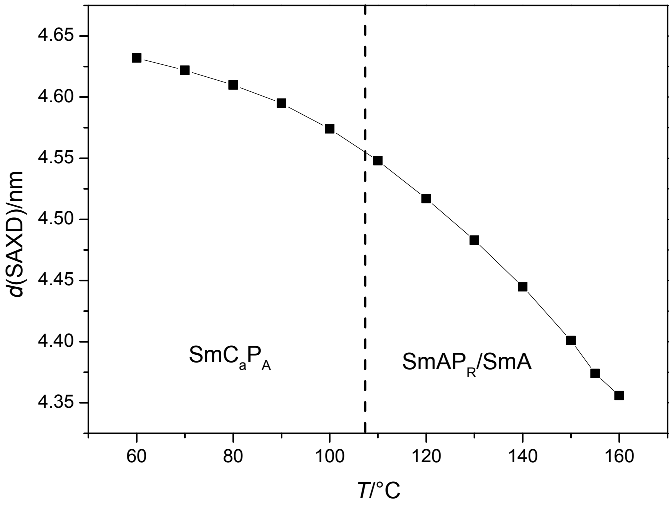

Figure S12. Temperature dependence of the *d*-values of compound **1/10**.

SmC$_a$P$_A$ at 100 °C

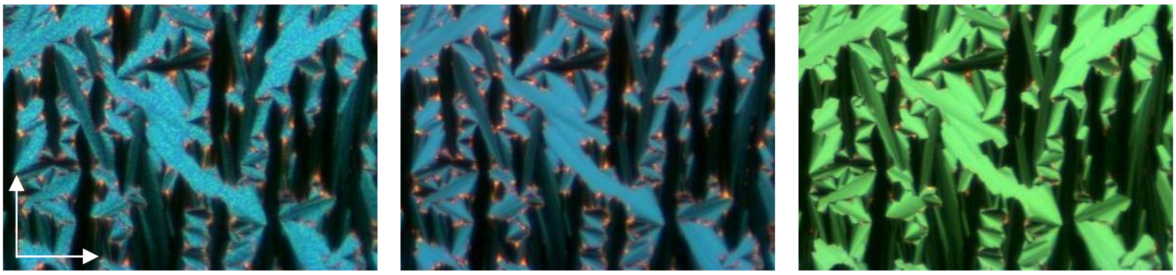

0V +2V/µm +4V/µm

SmC$_a$P$_A$ at 75 °C

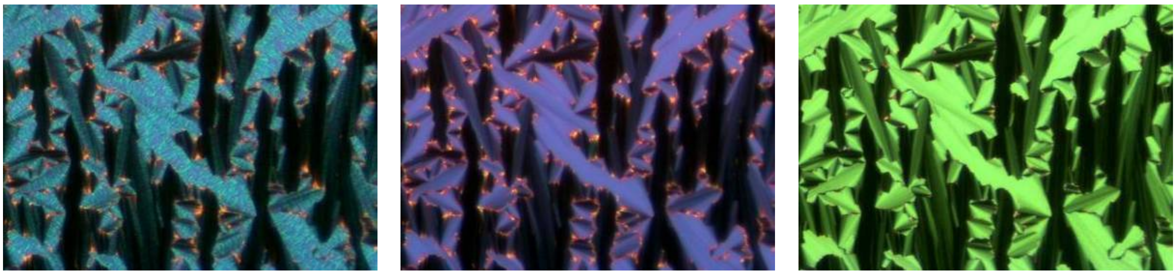

0V +2V/µm +4V/µm

Figure S13. Planar textures of **1/10** at the given applied voltages (6 µm ITO cell), indicating a switching around the molecular long axis in the SmC$_a$P$_A$ phase; for a more detailed discussion, see also Fig. S20.

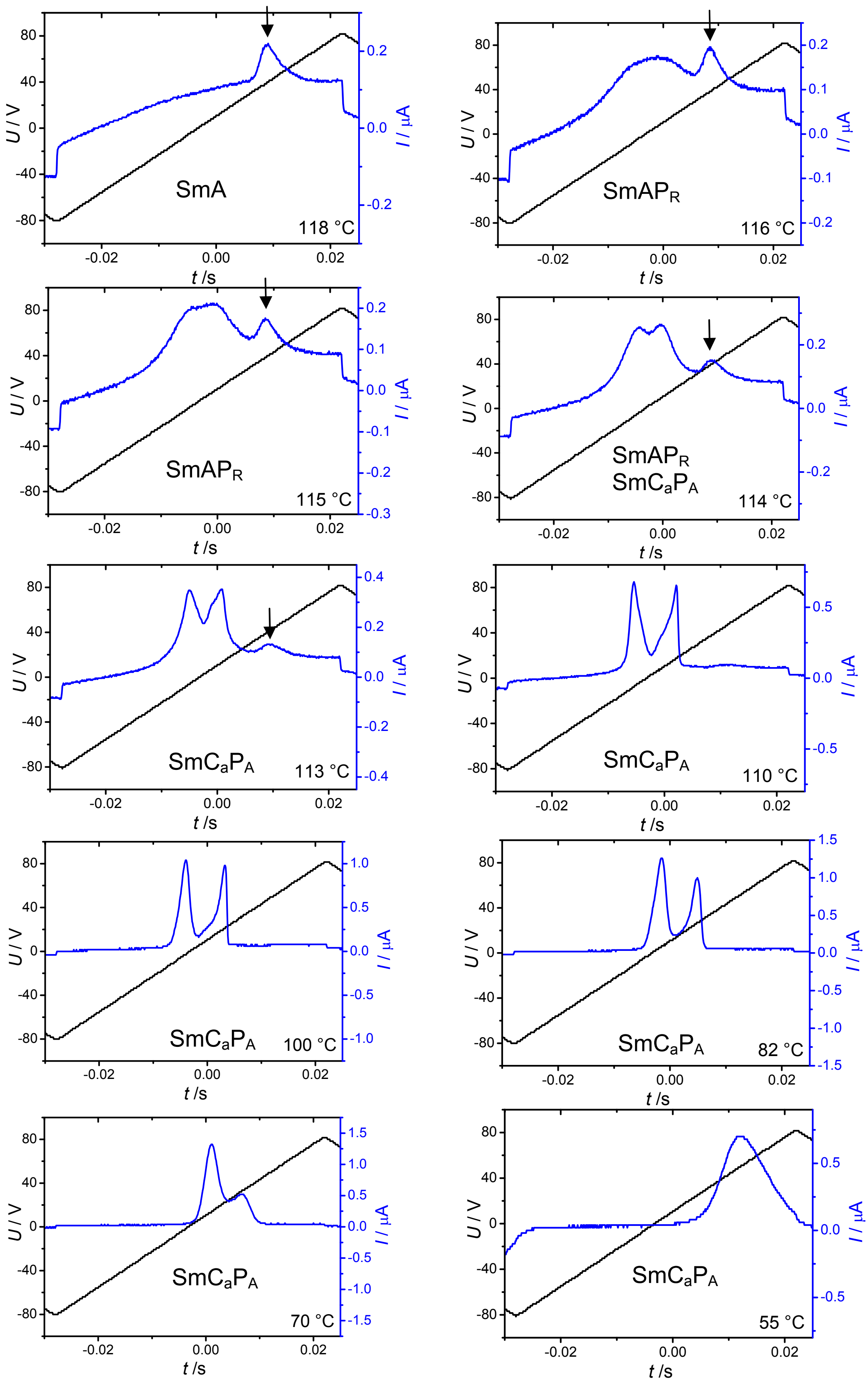

Figure S14. Development of the polarization current peaks of **1/10** depending on temperature; the sharp single peak indicated with an arrow is attributed to conductivity.

3.5 Compound 1/12

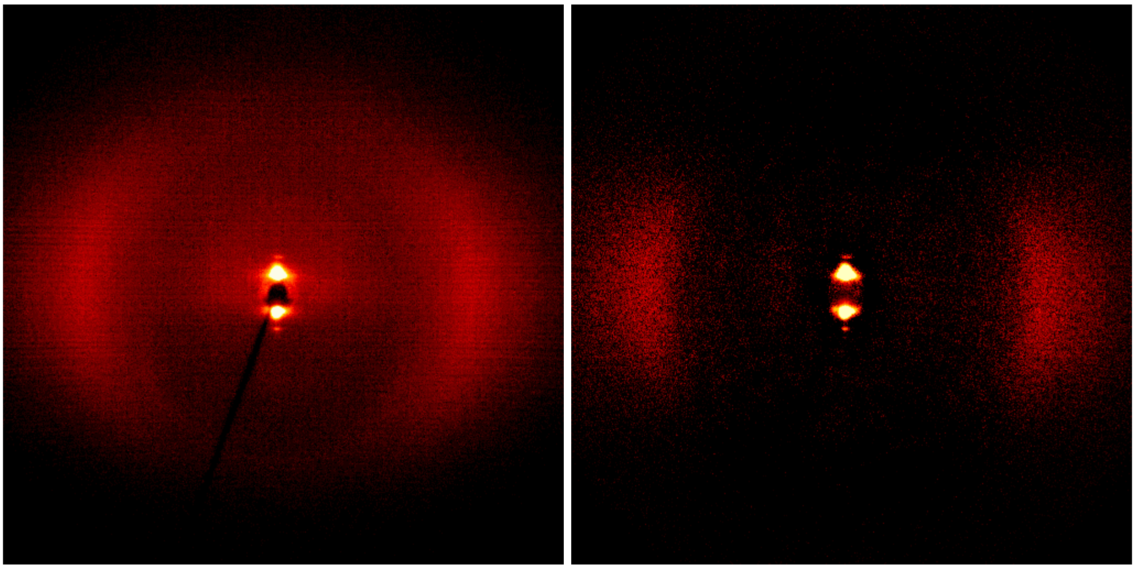

a) SmA at 130 °C

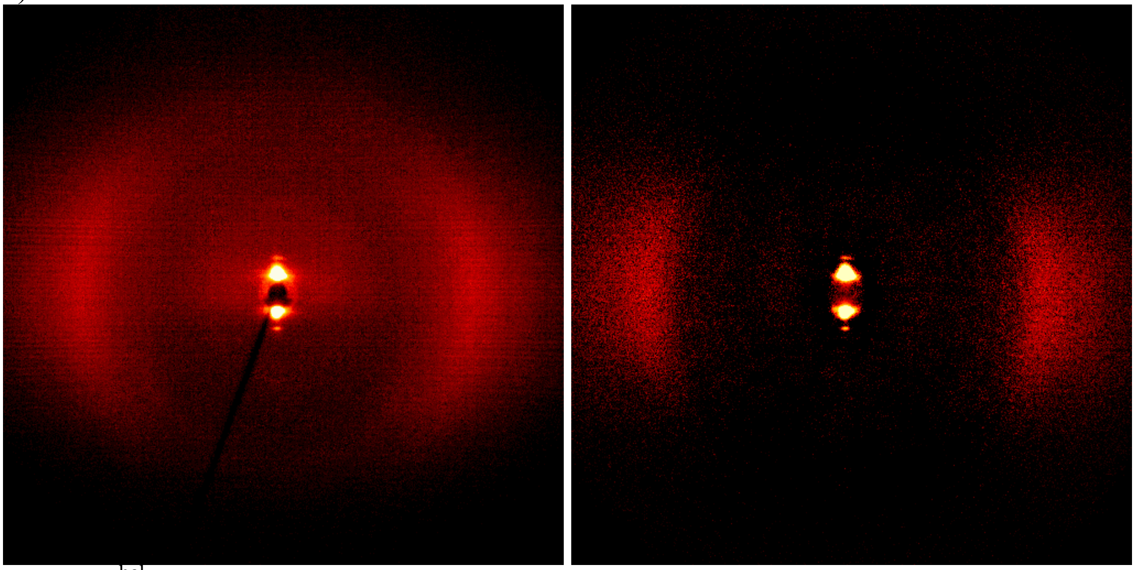

b) $SmsPF^{hel}$ at 110 °C

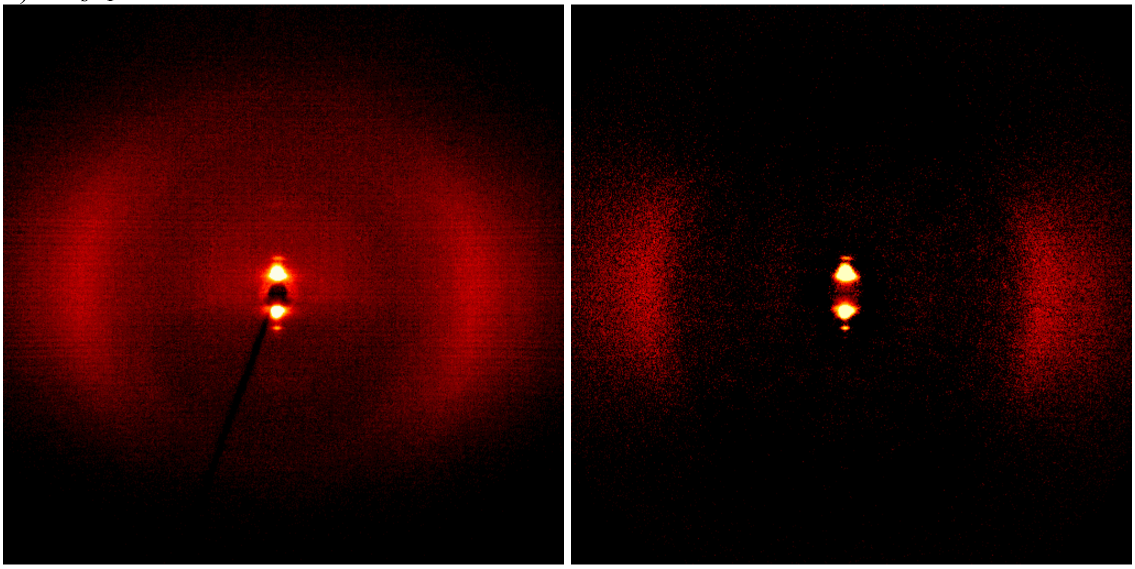

c) SmC_aP_A at 100 °C

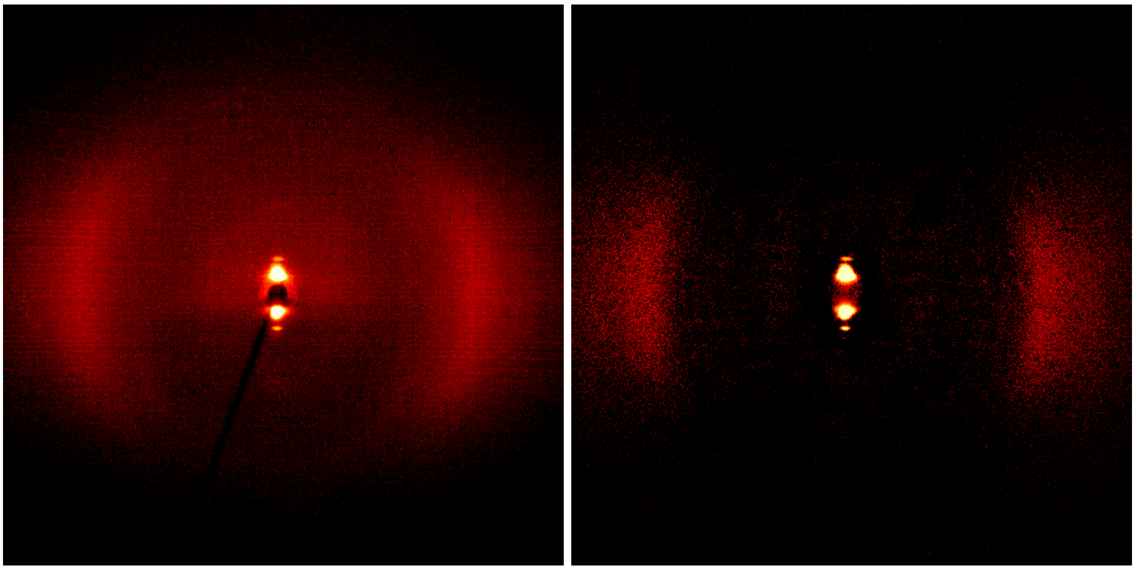

d) SmC_aP_A at 90 °C

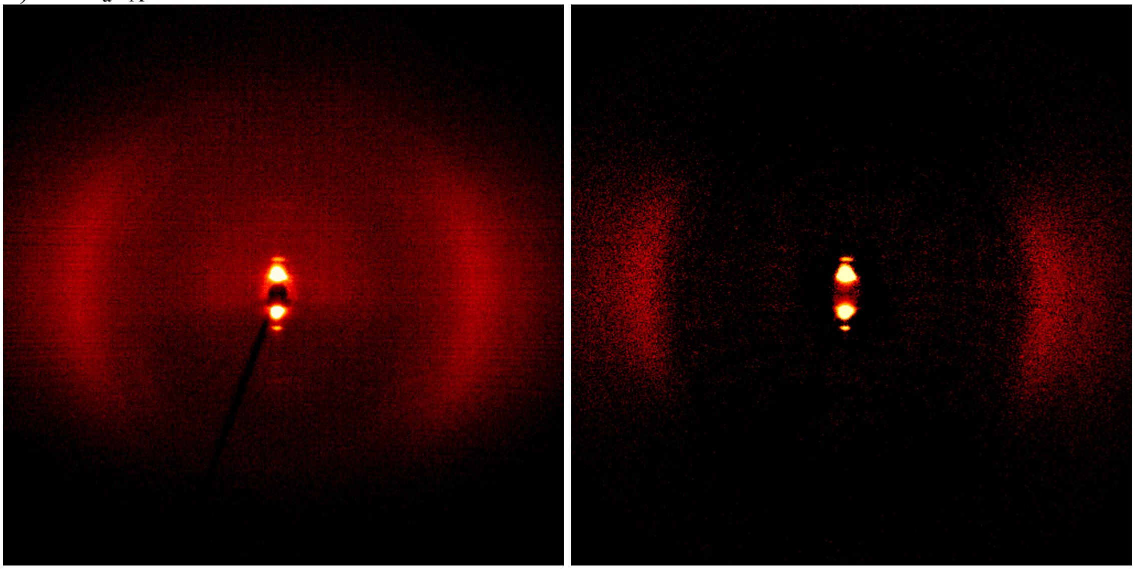

e) SmC_aP_A at 70 °C

Figure S15. XRD pattern of a surface aligned sample of **1/12** at the given temperatures, left original patterns, right patterns after subtraction of the isotropic pattern.

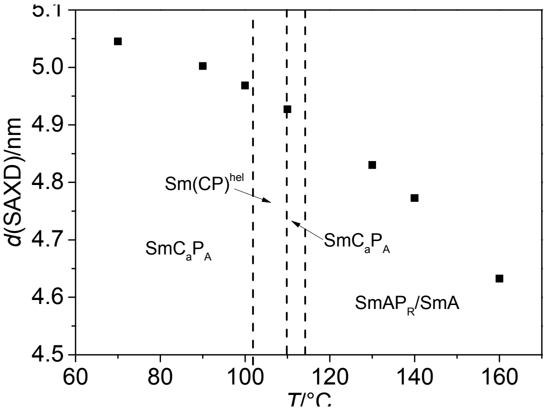

Figure S16. Temperature dependence of the *d*-values of compound **1/12**.

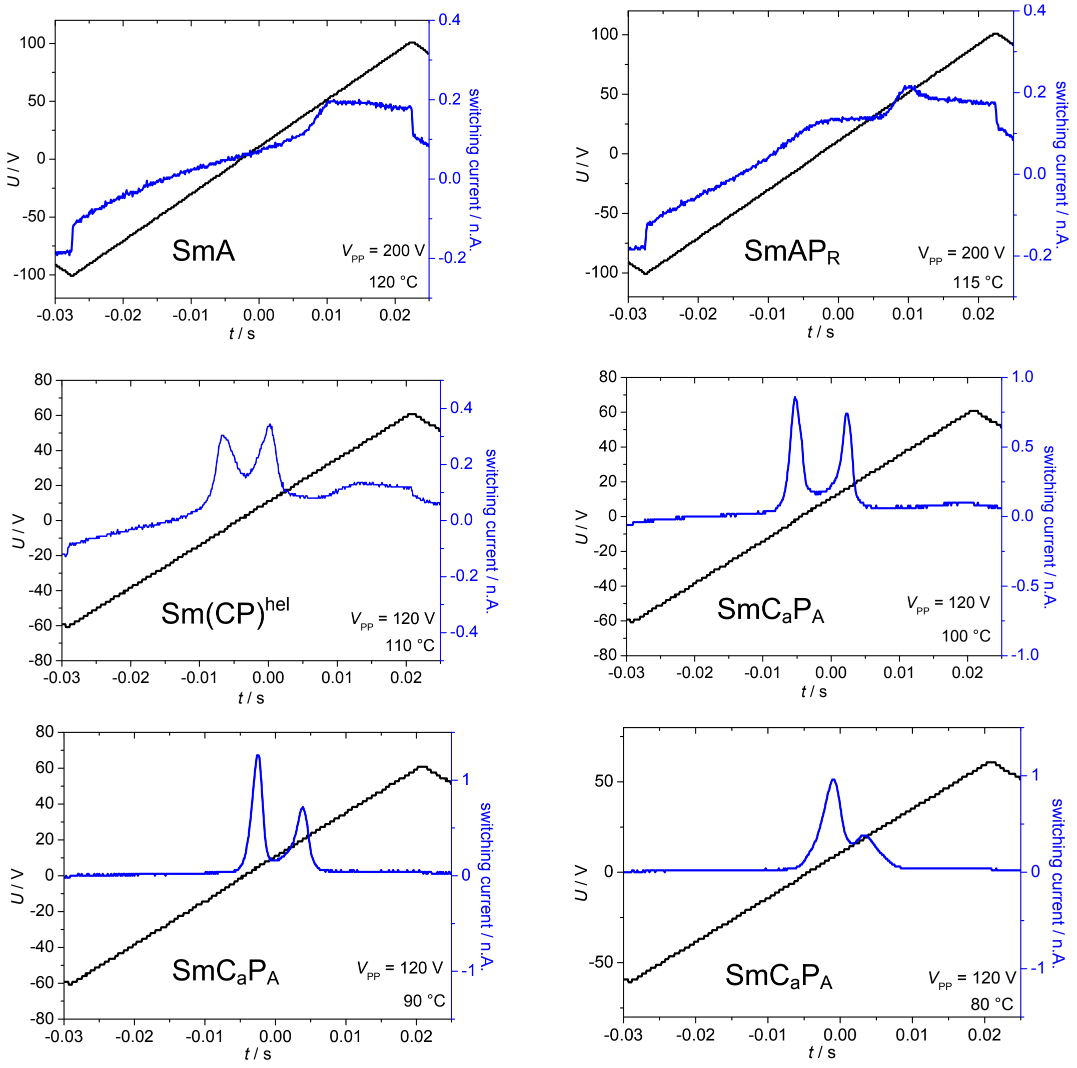

Figure S17. Development of the polarization current peaks of **1/12** depending on temperature.

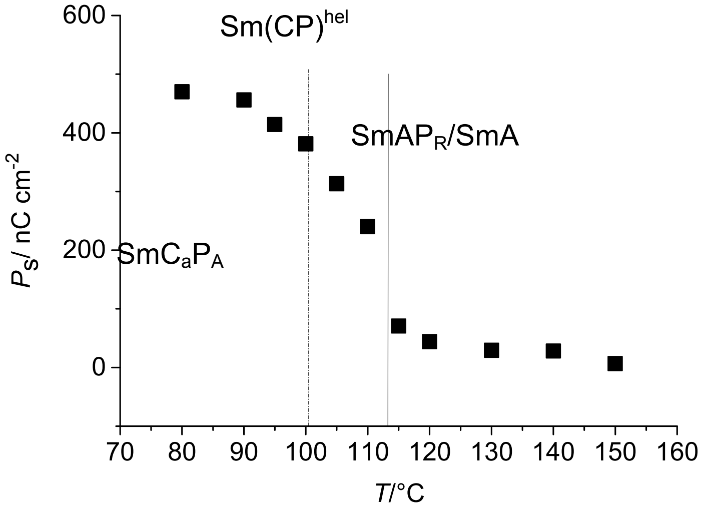

Figure S18. Development of the spontaneous polarization of **1/12** depending on temperature, measured with the triangular wave method in PI coated ITO cells (1cm^2, 160 Vpp, 10 Hz).

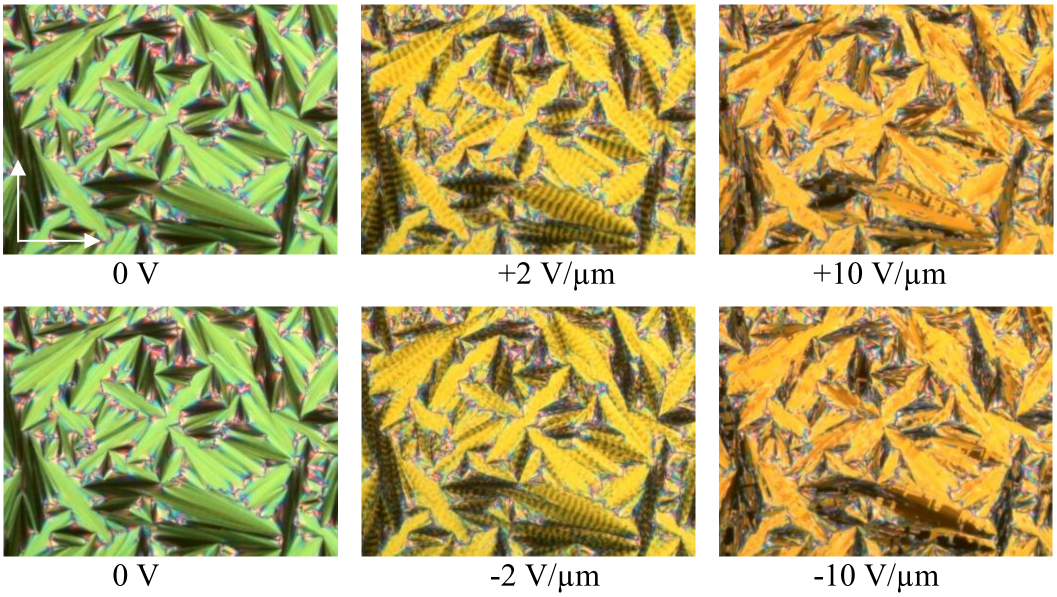

Figure S19. Development of tiger stripe and tilt-domain textures in the Sm(CP)^{hel} phase of **1/12** at T = 105 °C depending on applied voltage

Dielectric studies

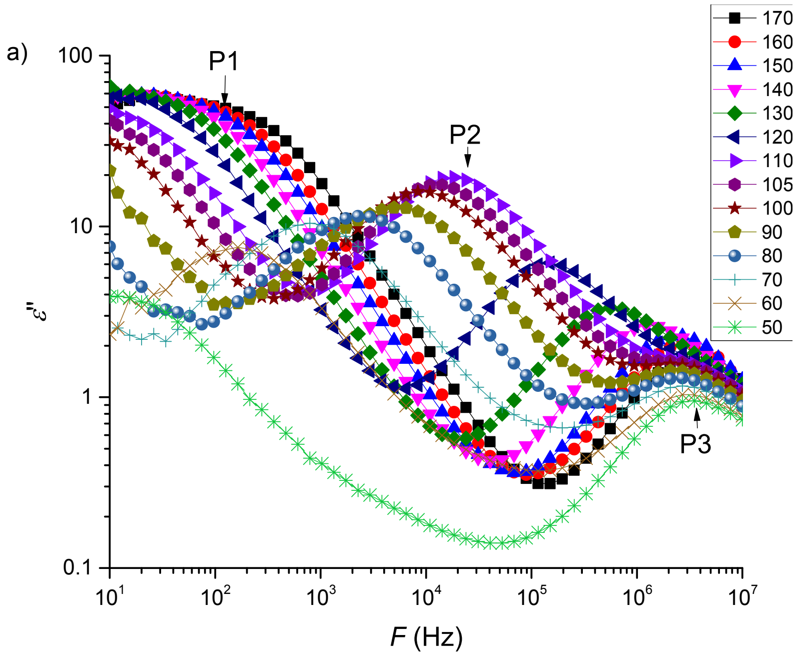

Figure S20. Plot of frequency dependence of the imaginary part of dielectric permittivity, ε'', of compound **1/12**, for selected temperatures including the SmA, SmAP$_R$, Sm(CP)^{hel} and SmC$_a$P$_A$ phases. P1, P2 and P3 are the relaxation processes observed in the measure frequency window.

A frequency dependence of the imaginary part of the dielectric permittivity for compound **1/12** at selected temperatures is shown in Fig. S20. Three relaxation processes, P1, P2 and P3 were observed in the measured frequency range. The dielectric strength $\delta\varepsilon$ and the relaxation frequency f_R P2 and P3 are obtained by fitting the relaxation spectra to the Havriliak-Negami equation. The low frequency process P1 is attributed to conductivity. The high frequency relaxation process P3 was observed in the measured frequency range only below 140 °C and

is assigned to rotation around the short axis. The plot of temperature dependence of relaxation strength and frequency are given in Fig. S21. Initially, between 140 °C to ~120 °C, the relaxation frequency of P3 decreases and the corresponding relaxation strength increases (by 50%) following a 2S+1 dependence (S being the orientational order parameter), as the sample is cooled down. With further decrease in temperature no significant change is observed in both $\delta\varepsilon_3$ and $f_{R,3}$ until the sample reaches ~80 °C, after which the $\delta\varepsilon_3$ decreases (from 3.5 to 2.5) and $f_{R,3}$ increases slightly. The process P2 (medium frequency relaxation process, see Fig. S20) exists in the measured frequency range in all of the liquid crystal phases. The process can be assigned to the polar switching mechanism and Fig. 10 in the main text shows the temperature dependence of dielectric strength and relaxation frequency for P2.

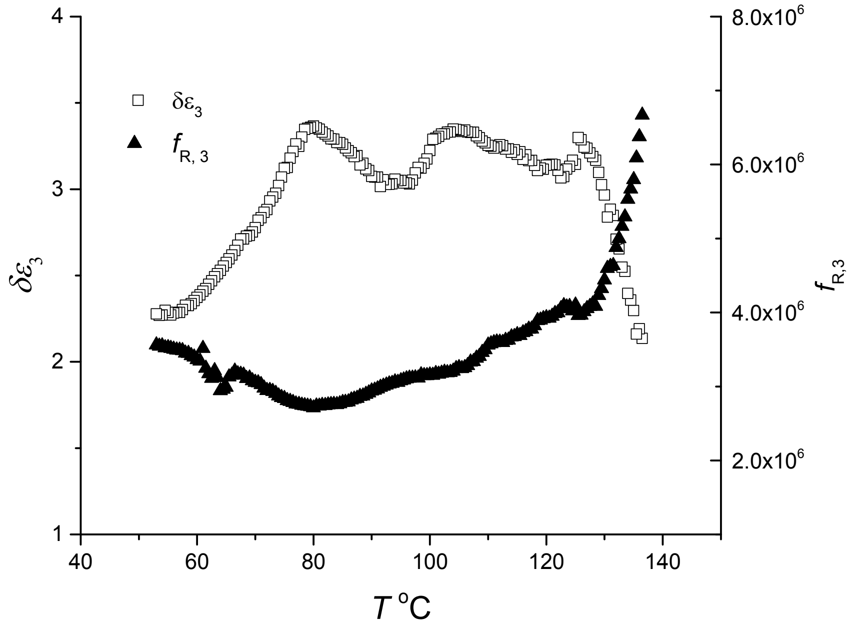

Figure S21. The plot of dielectric relaxation strength, $\delta\varepsilon_3$ (open symbol), and relaxation frequency, $f_{R,3}$ (closed symbol), for process P3 of compound **1/12**, as a function of temperature.

3.6 Compound 1/14

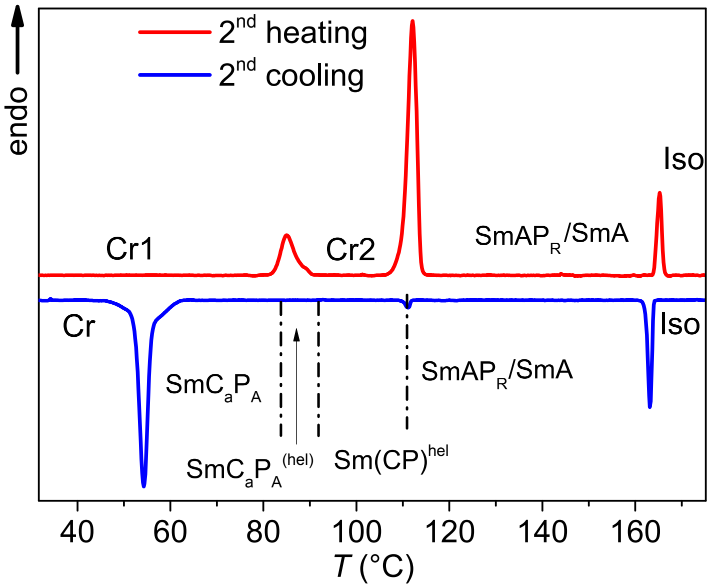

Figure S22. DSC traces of compound **1/14** (10 K min^{-1}).

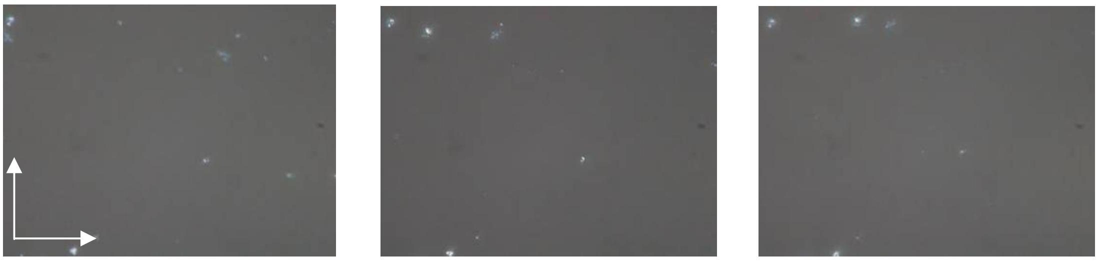

SmA at 120 °C | SmA-Sm(CP)^{hel} transition at 110 °C | Sm(CP)^{hel} at 100 °C

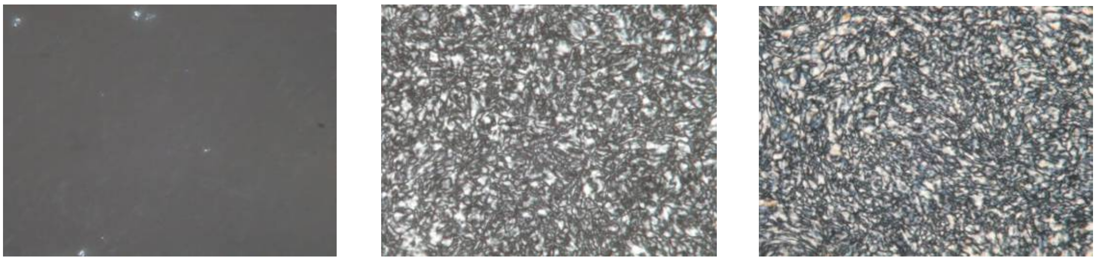

Sm(CP)^{hel} 92 °C | SmC$_a$P$_A$ 87 °C | SmC$_a$P$_A$ 72 °C

Figure S23. Textures of a homeotropically aligned sample of **1/14** depending on temperature.

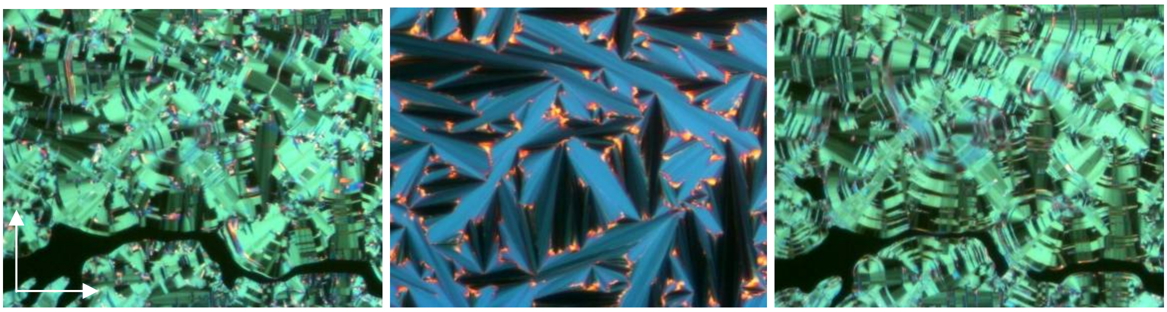

-30 V/µm | 0V | +30 V/µm

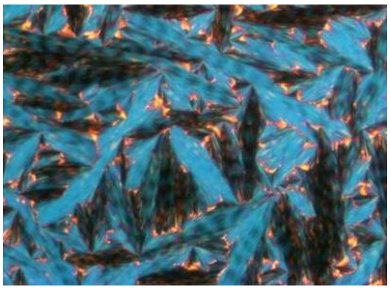

+2.8 V/µm

Figure S24. Planar textures of **1/14** in the Sm(CP)^{hel} at 112 °C at 0V (upper line, middle) and under an applied DC field showing the field induced tilt-domain texture (in a 6 µm ITO cell, right, left); at lower voltage (2.8 V) the tiger stripe pattern is observed.

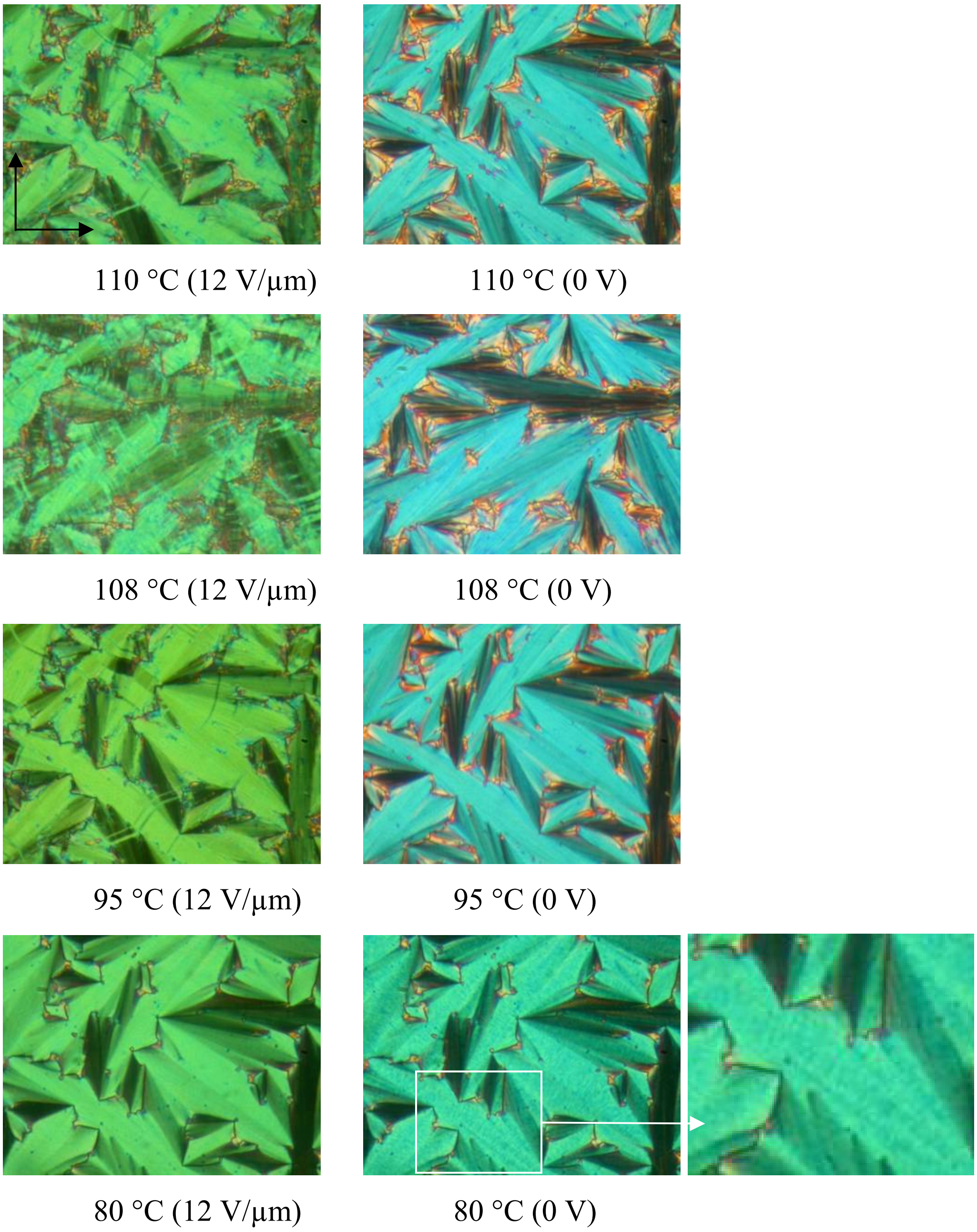

Figure S25. Planar textures of **1/14** in the Sm(CP)^{hel} range (top rows) and in the SmC_aP_A phase (bottom row) under an applied DC field (12V, in a 6 µm ITO cell, left) and after switching off the field at 0V (right).

3.7 Compound 1/16

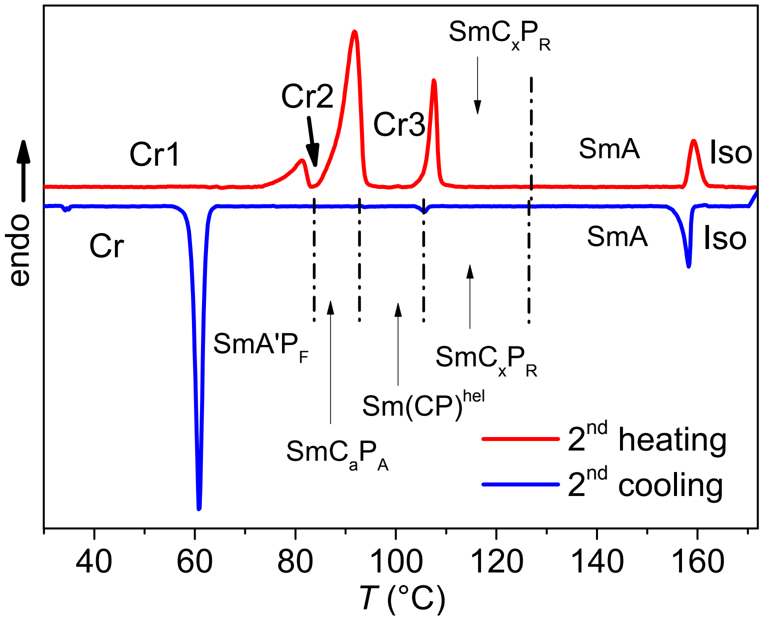

Figure S26. DSC traces of compound **1/16** (10 K min^{-1}).

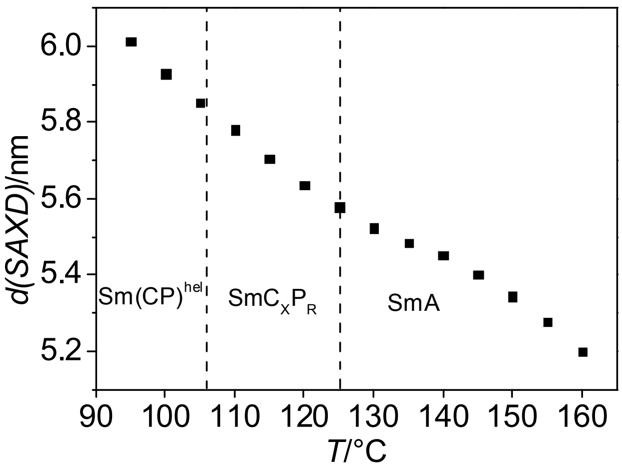

Figure S27. Temperature dependence of the *d*-values of compound **1/16**.

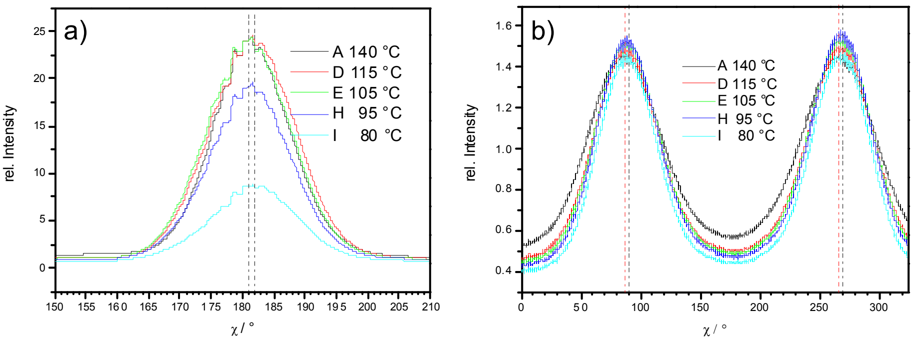

Figure S28. Chi-scans a) over the small angle range (θ = 15-25°) and the wide angle range (θ =15-25°) of the 2D XRD pattern of a surface aligned sample of compound **1/16** in the different LC phase ranges; the peak maximum of the small angle scattering are shifted by +1…+2° form 180° and the maxima of the wide angle scatterings are shifted by -2 … -3° from the 90 and 270° positions, meaning that a tilt of 3-5° would be possible, which is within the error (± 3°) of this method.

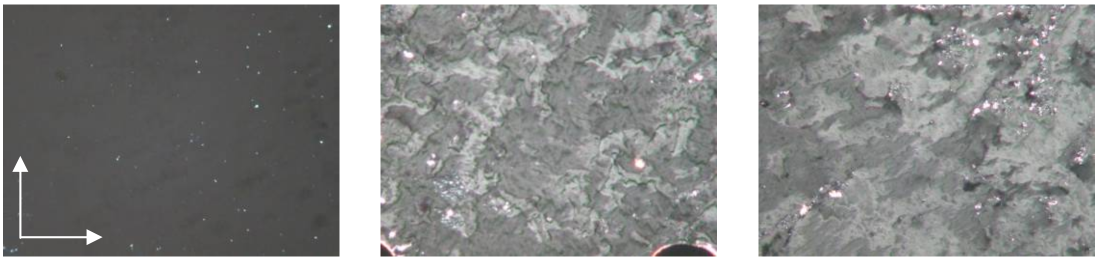

130 °C 120 °C 110 °C

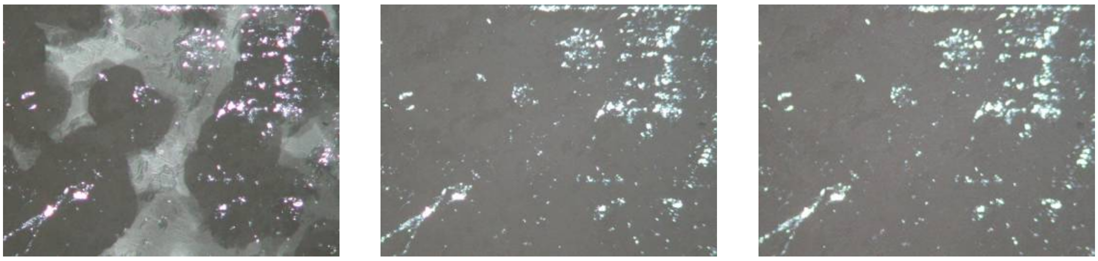

107 °C 105 °C 100 °C

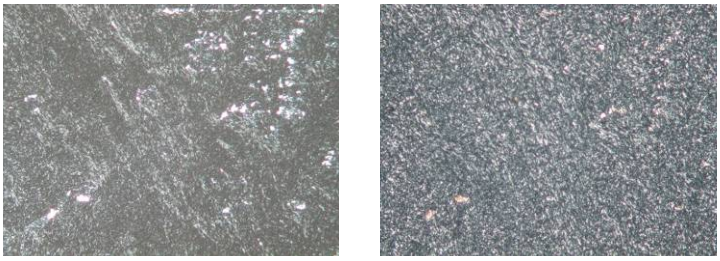

85 °C 80 °C

Figure S29. Textures of a homeotropic sample of **1/16** depending on temperature.

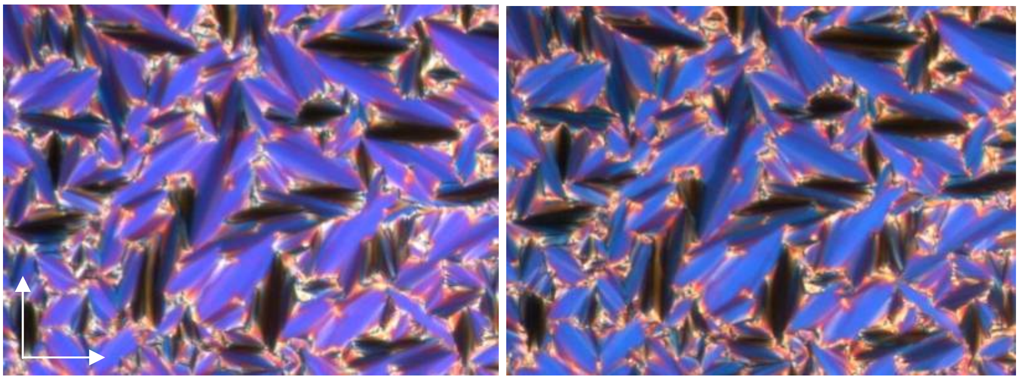

SmA at 130 °C SmC_xP_R at 100 °C

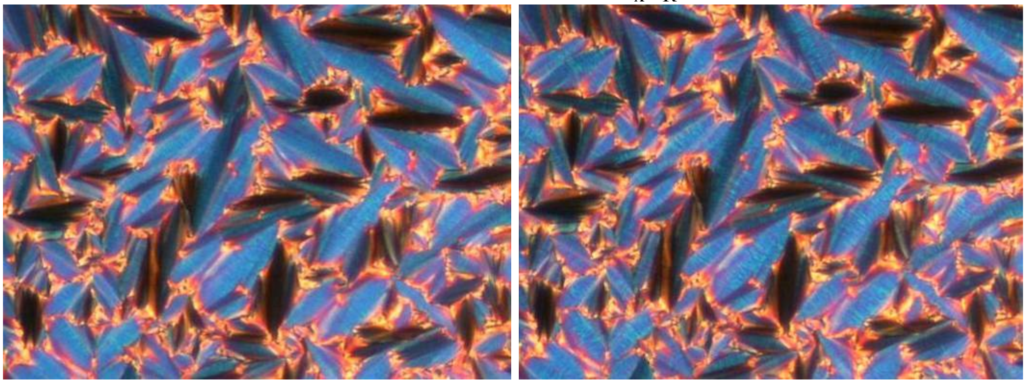

$Sm(CP)^{hel}$ at 90 °C $SmA'P_F$ at 80 °C

Figure S30. Textures of a planar sample of **1/16** depending on temperature as observed on cooling.

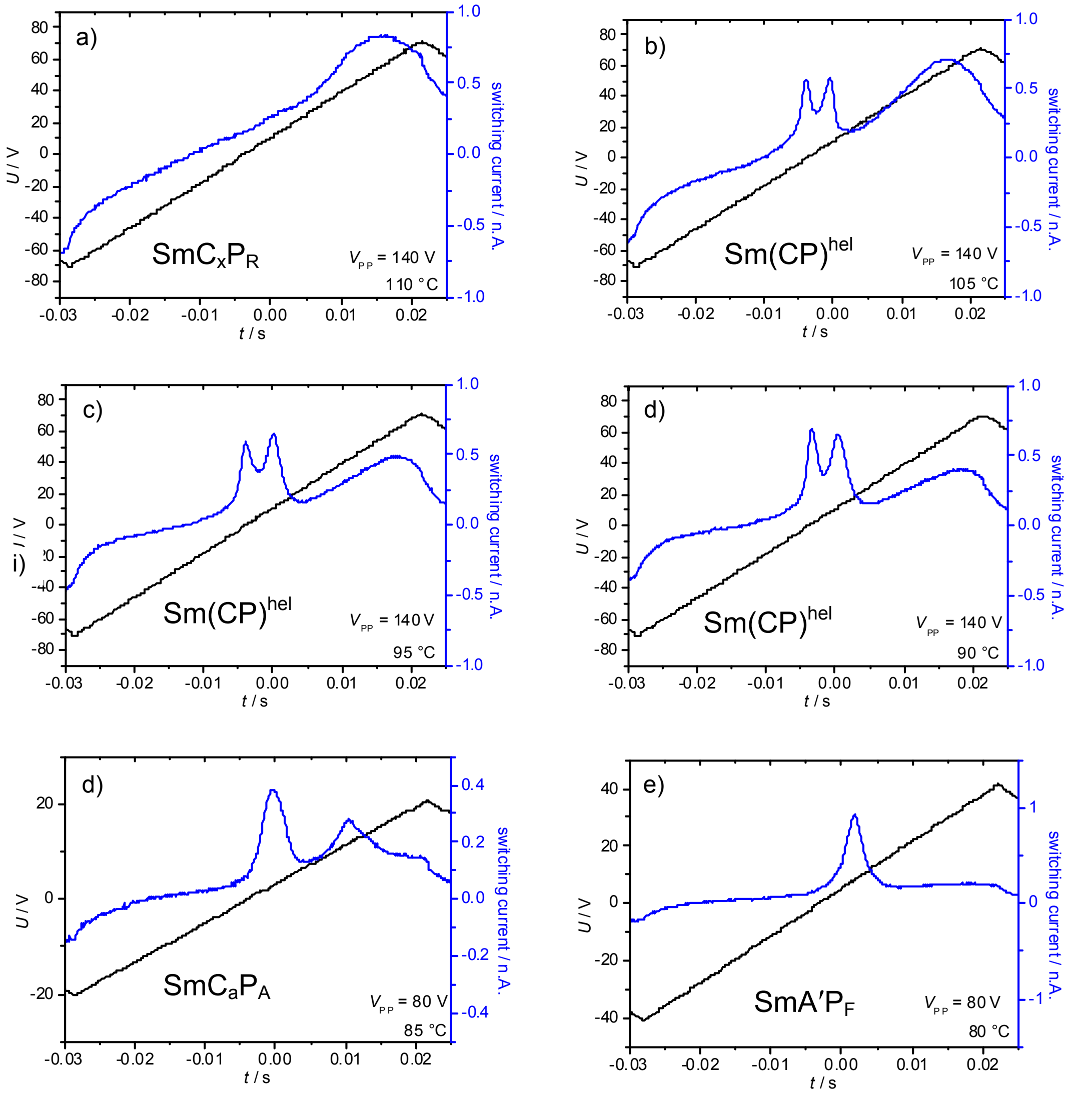

Figure S31. Development of the polarization current curves of **1/16** depending on temperature.

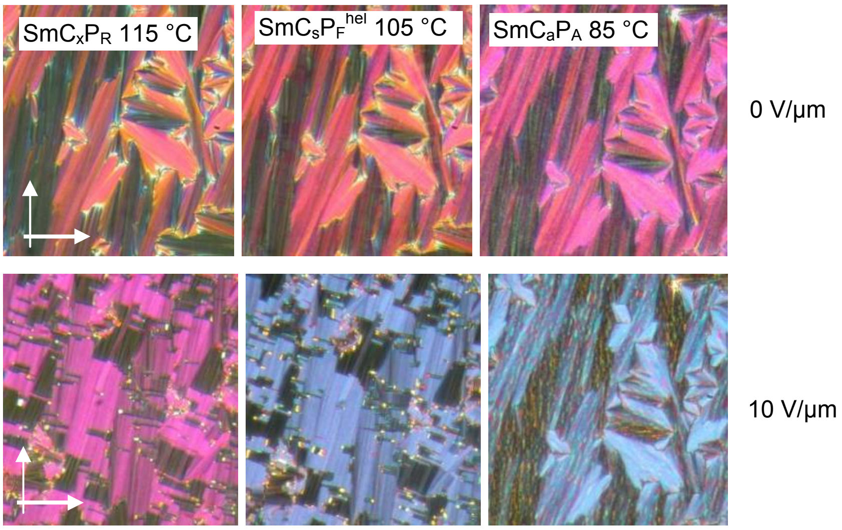

Figure S32. Planar textures of **1/16** in the in the distinct phases at 0V (upper row) and under an applied DC field (in a 6 μm ITO cell, lower).

3.8 Compound 1/18

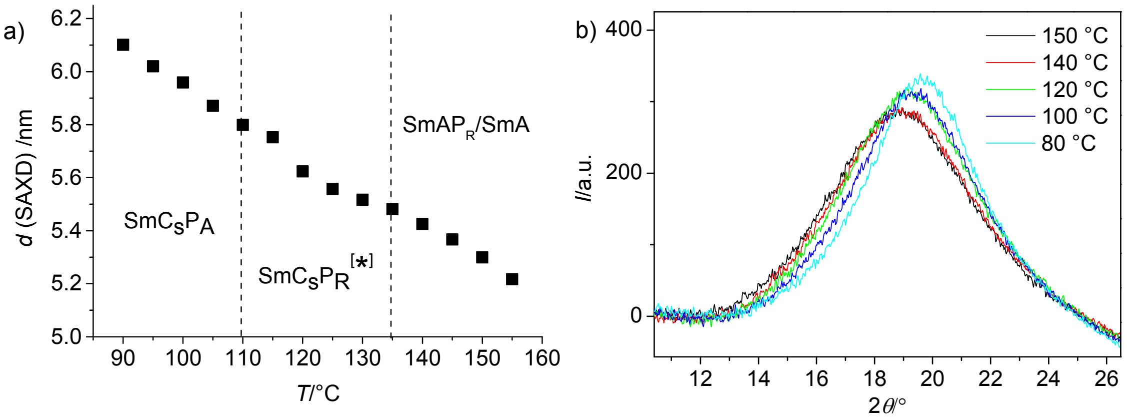

Figure S33. a) Temperature dependence of the *d*-values of the small angle scattering and b) the profile of the diffuse wide angle scattering of compound **1/18**.

<u>*Rotation of the analyzer*</u>

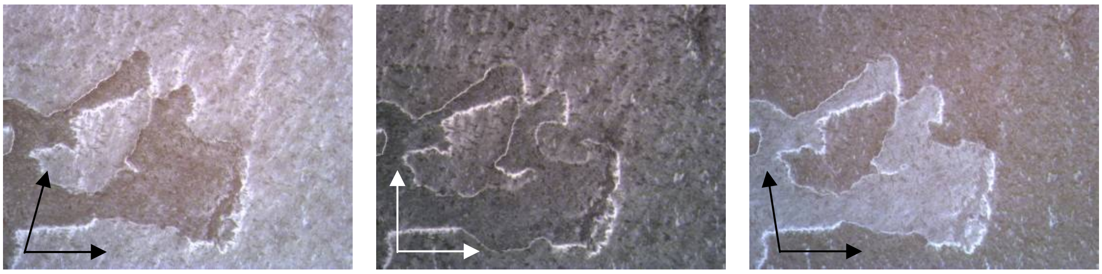

-10° 0° +10°

<u>*Rotation of the sample between crossed polarizers*</u>

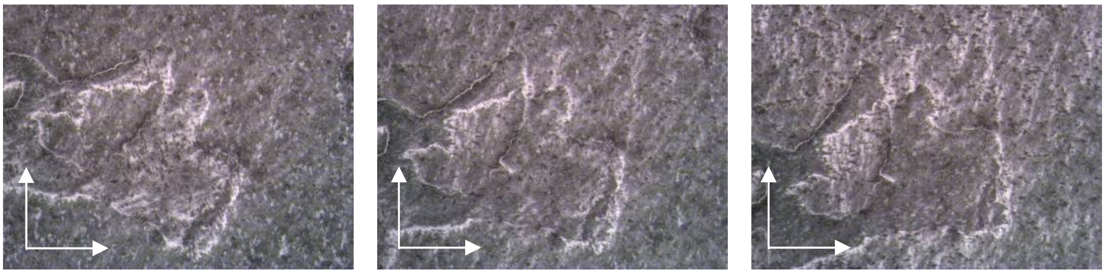

-15° 0° +15°

Figure S34. Chiral domains in the $SmC_sP_R{}^{[*]}$ phase of compound **1/18** at $T = 120$ °C.

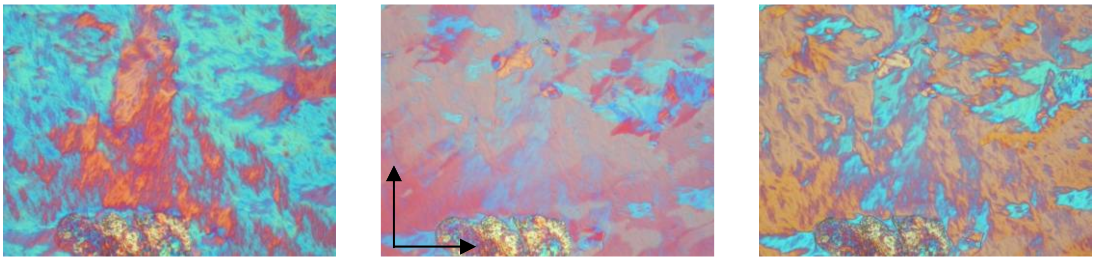

120 °C 105 °C 100 °C

Figure S35. Inversion of birefringence as observed for a homeotropic sample of **1/18** between crossed polarizers with additional λ-retarder plate.

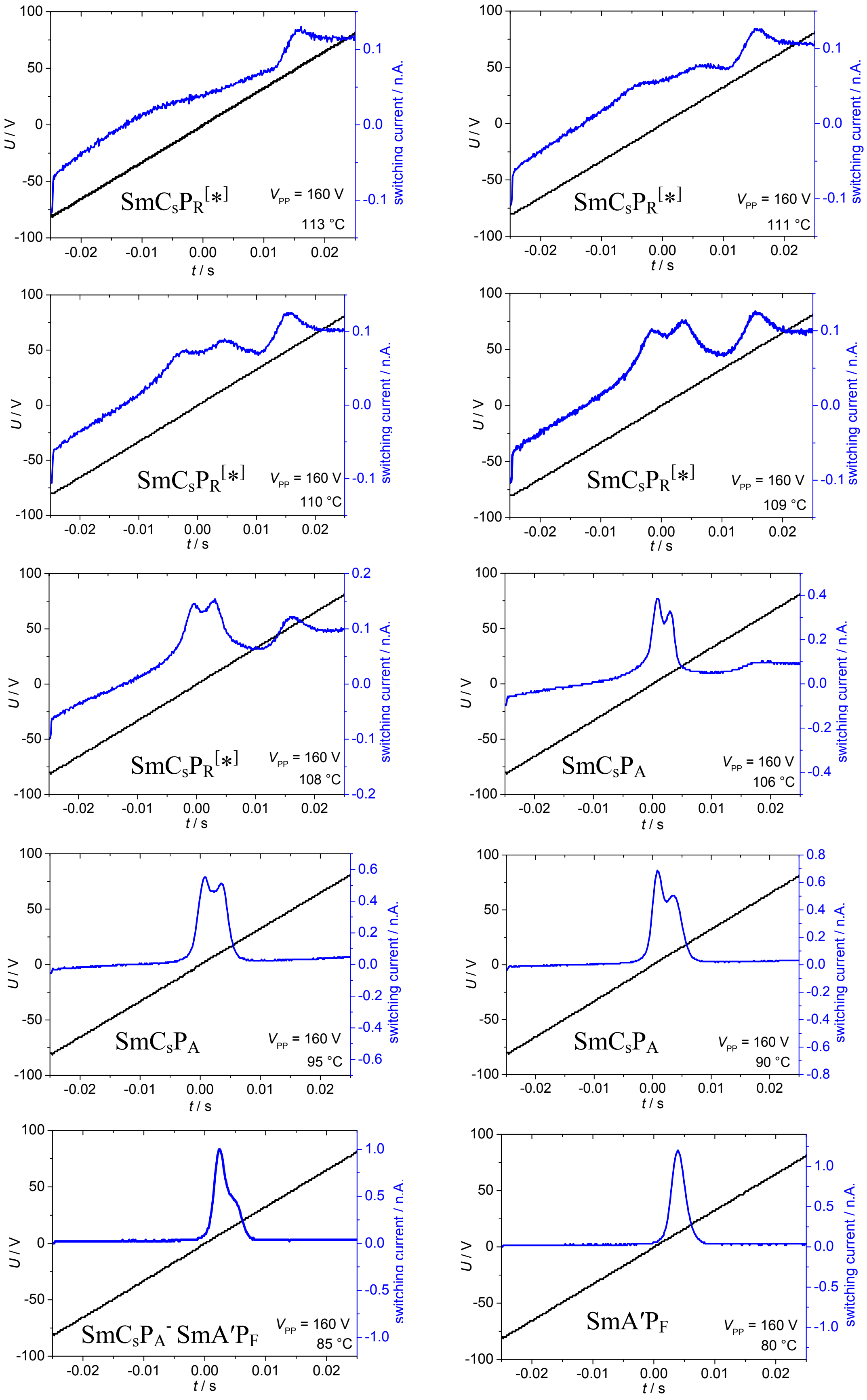

Figure S36. Development of the polarization current peaks of **1/18** depending on temperature.

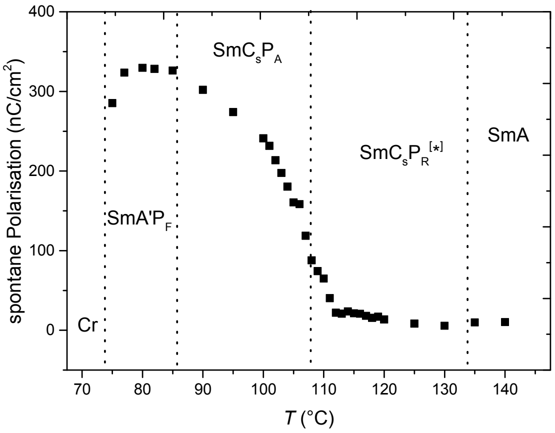

Figure S37. Development of the spontaneous polarization of **1/18** depending on temperature, measured with the triangular wave method in PI coated ITO cells (1cm^2, 160 Vpp, 10 Hz).

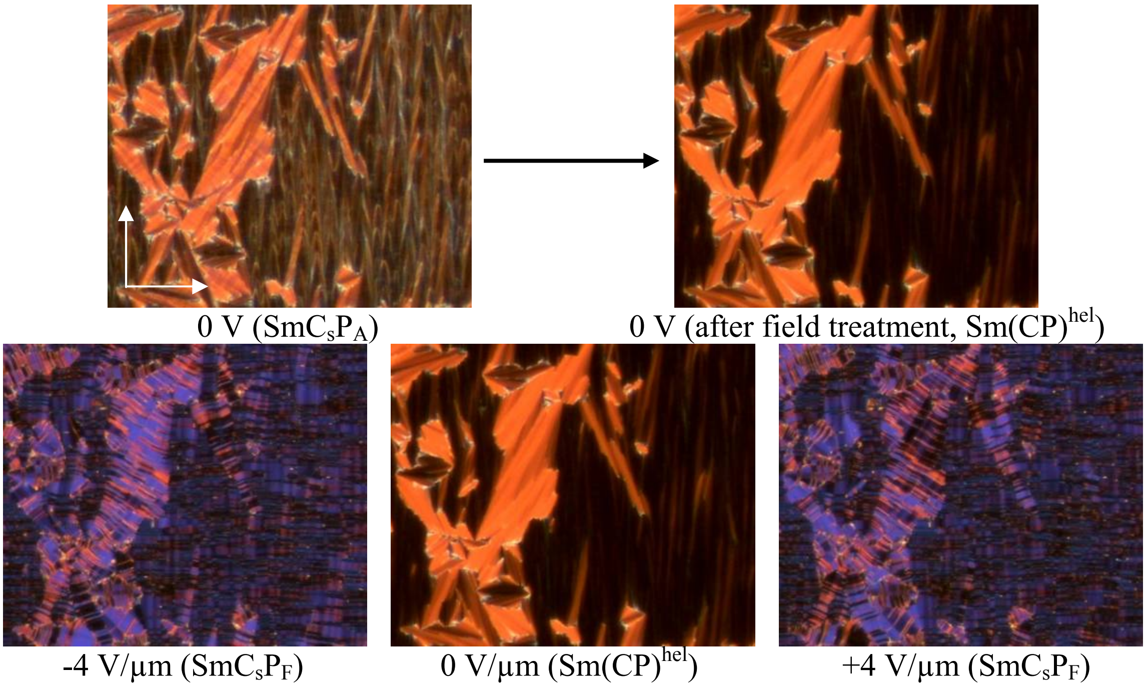

Figure S38. Induction of the heliconical $Sm(CP)^{hel}$ phase in the SmC_sP_A phase at T = 105°C (6 µm, PI-coated ITO cell). Upper row: pristine texture of the SmC_sP_A phase (left) and texture after application of an AC field (200 V, 10Hz, 2 s, right) and lower line: switching between $Sm(CP)^{hel}$ and the field induced SmC_sP_F states by rotation on a cone under an DC field.

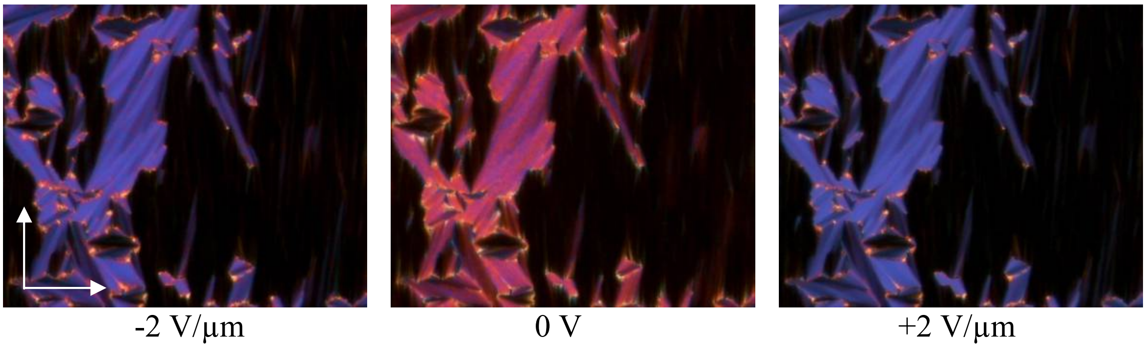

Figure S39. Switching by rotation around the long axis in the SmA'P_F phase of **1/18** at T = 80 °C (6 µm, PI-coated ITO cell) under a DC field.

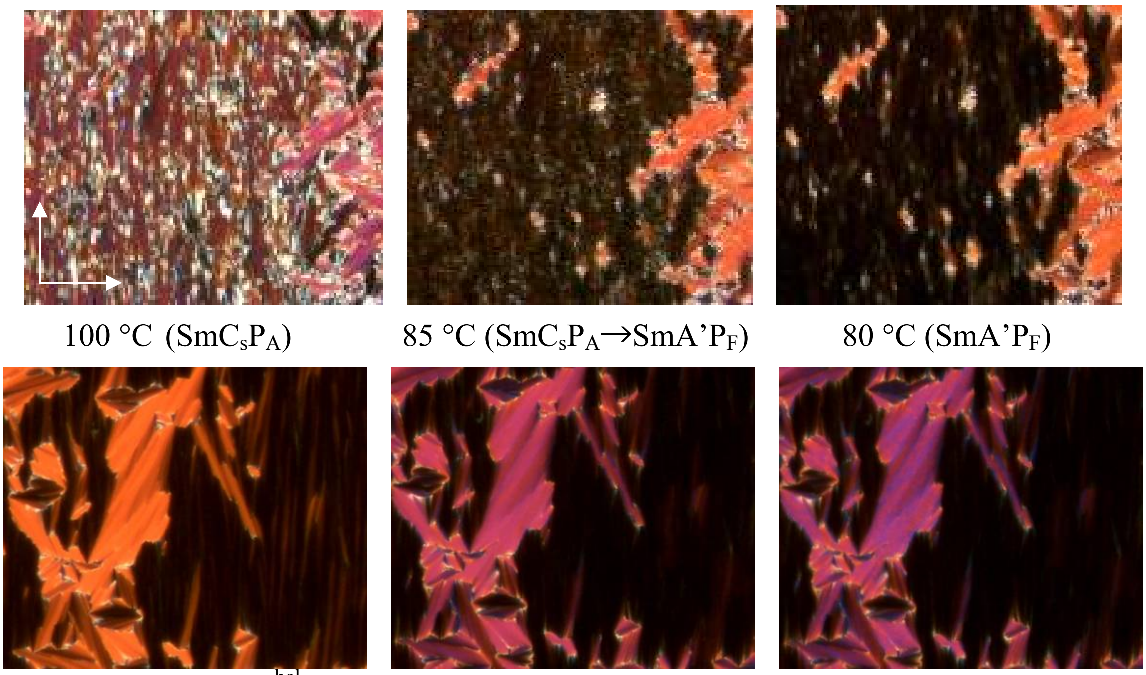

100 °C (SmC_sP_A) 85 °C ($SmC_sP_A \rightarrow SmA'P_F$) 80 °C ($SmA'P_F$)

105 °C ($Sm(CP)^{hel}$) 83 °C ($SmA'P_F$) 80 °C ($SmA'P_F$)

Figure S40. Change of the textures of **1/18** at the phase transitions (upper line) in the pristine state and (lower line) after treatment with an AC field.

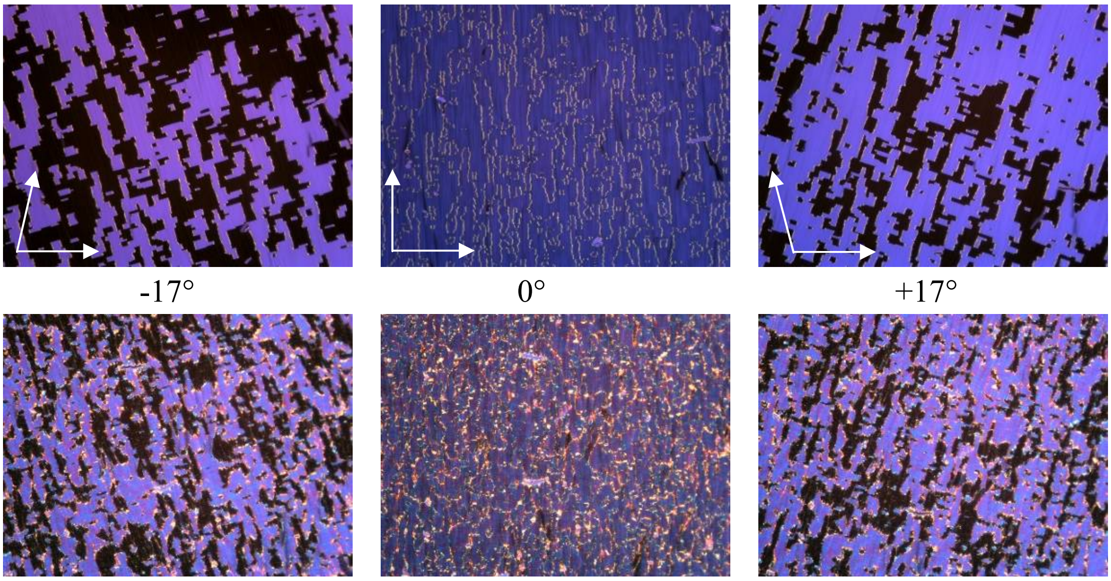

-17° 0° +17°

-14° 0° +14°

Figure S41. Tilt domains in the field induced planar textures of **1/18** in the $SmC_sP_R^{[*]}$ range at 115 °C (upper row) and in the field-induced SmC_sP_F phase at 90 °C (lower row) between crossed polarizers (middle) and after rotation of the sample between the crossed polarizers by the given angles (left, right) in a 6 µm ITO cell under an voltage of 160 V.

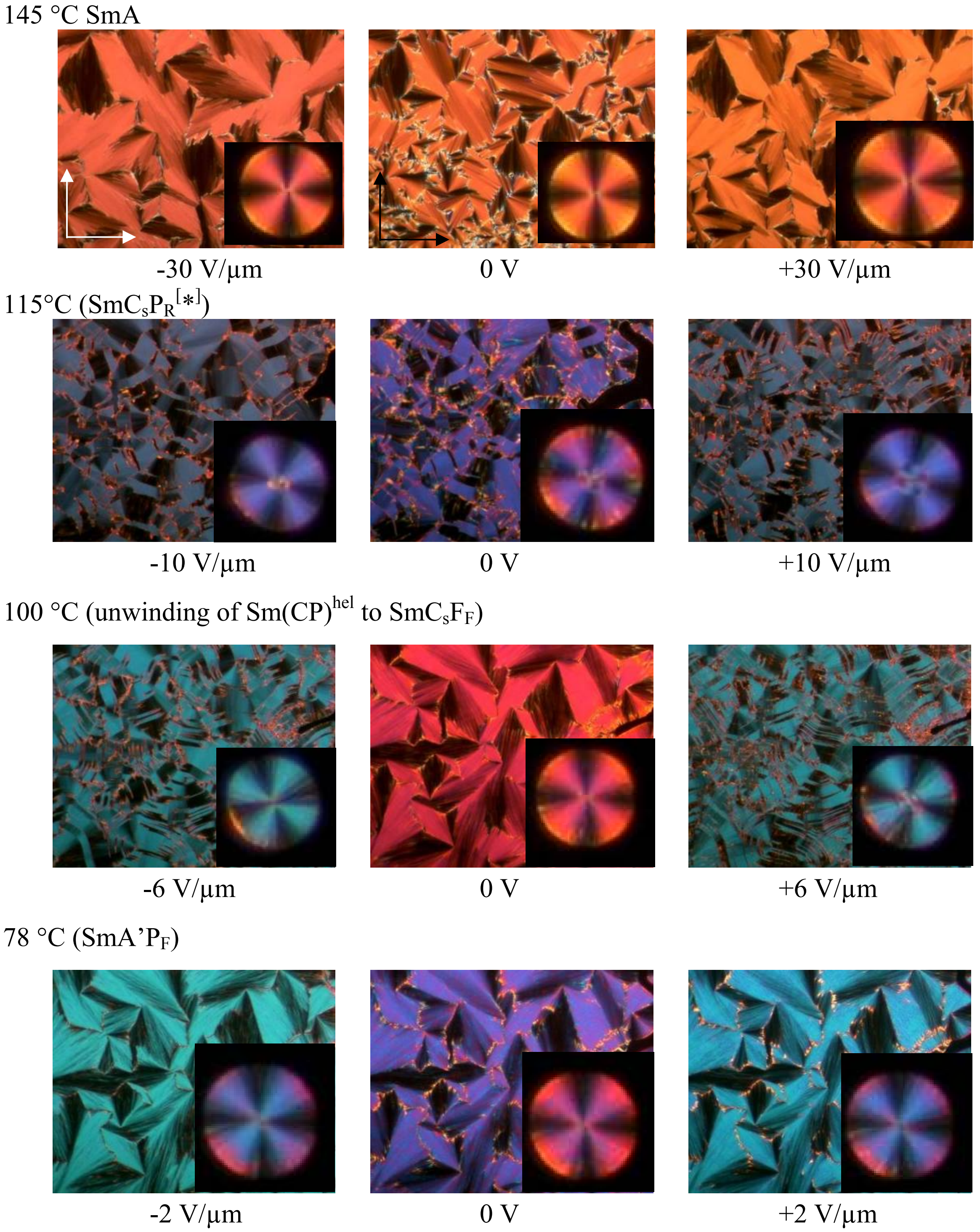

Figure S42. Planar textures of **1/18** in the distinct phases at 0V (middle) and under an applied DC field (in a 6 µm ITO cell, right, left).

3.9 Compound 1/20

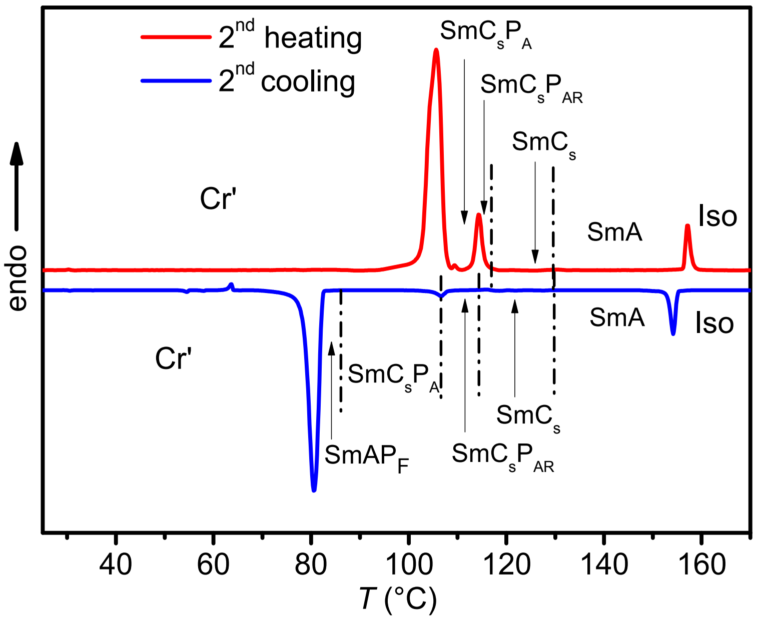

Figure S43. DSC traces of compound **1/20** (10 K min^{-1}).

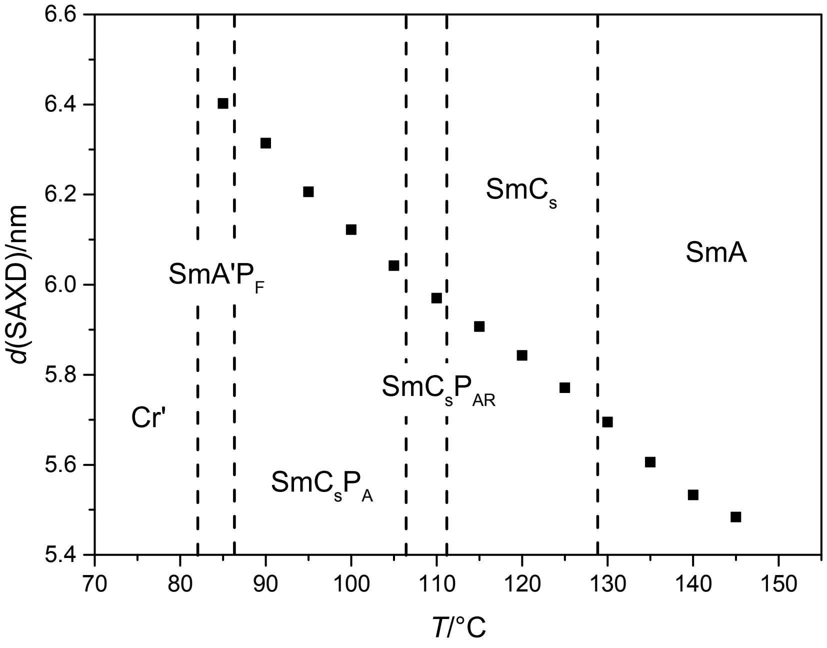

Figure S44. Temperature dependence of the d-values of the small angle scattering of compound **1/20**.

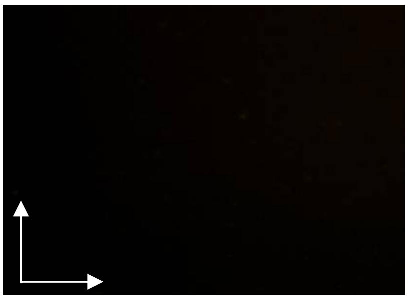
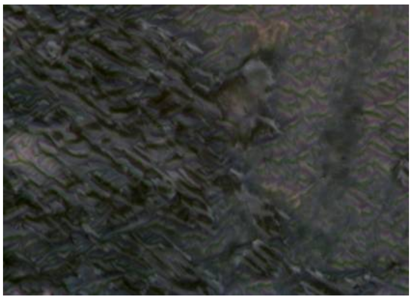
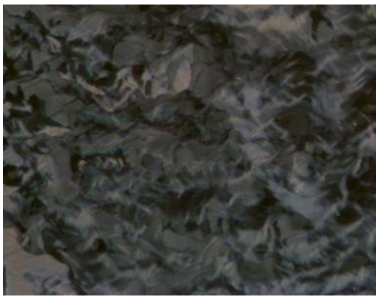

SmA at T = 140 °C　　SmC$_s$ at T = 140 °C　　SmC$_s$P$_{AR}$ at T = 128 °C

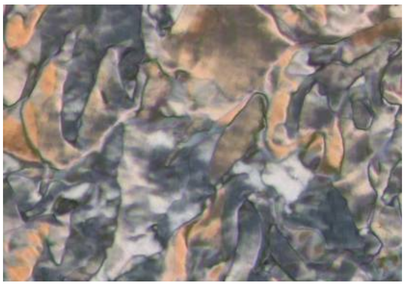
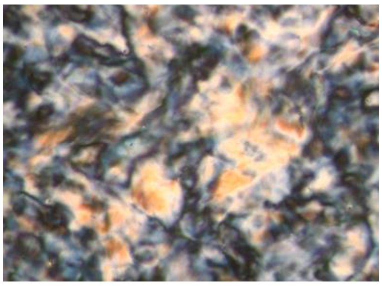

SmC$_s$P$_A$ at T = 100 °C　　SmA’P$_F$ at T = 85 °C

Figure S45. Textures of a homeotropically aligned sample of **1/20** depending on temperature (anchoring transition is assumed to lead to higher birefringence in the SmA’P$_F$ phase.

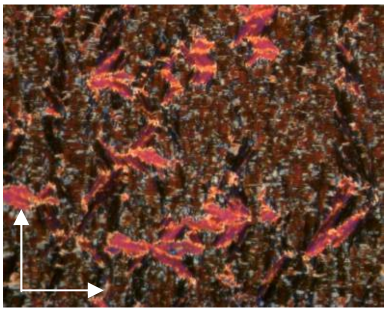
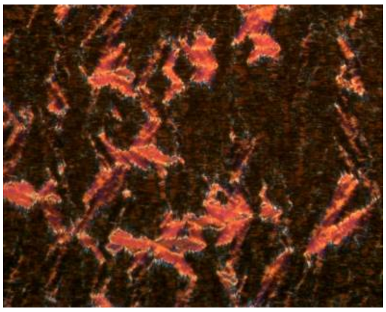
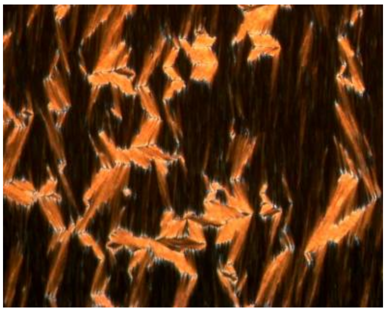

90 °C (SmC$_s$P$_A$)　　86 °C (SmC$_s$P$_A$→SmA’P$_F$)　　80 °C (SmA’P$_F$)

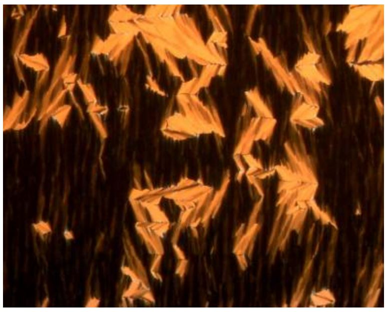
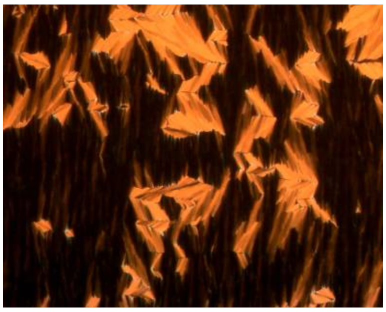
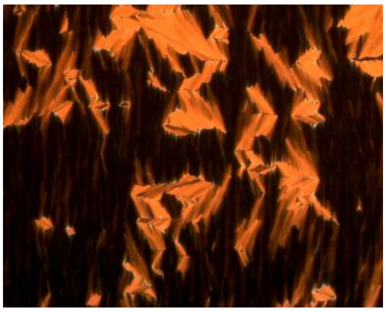

90 °C (Sm(CP)^{hel})　　86 °C (Sm(CP)^{hel} →SmA’P$_F$)　　80 °C (SmA’P$_F$)

Figure S46. Textures of **1/20** (6 µm, PI-coated ITO cell) in the ground state (upper line) and after application of an AC field (200 Vpp, lower line).

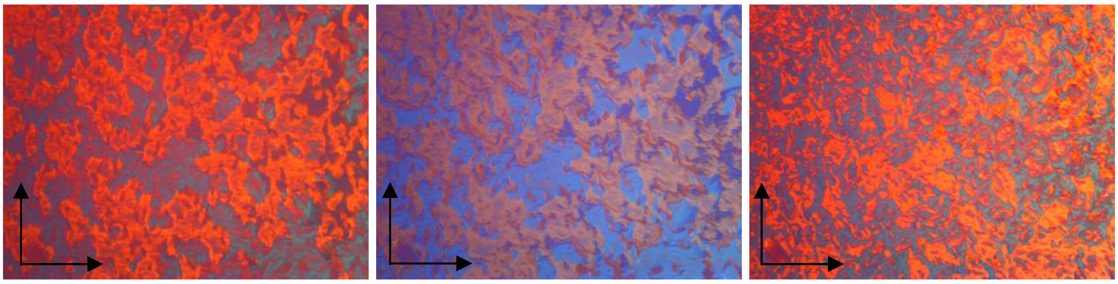

120 °C 105 °C 98 °C

Figure S47. Inversion of birefringence as observed for a homeotropic sample of **1/20** between crossed polarizers with additional λ-retarder plate.

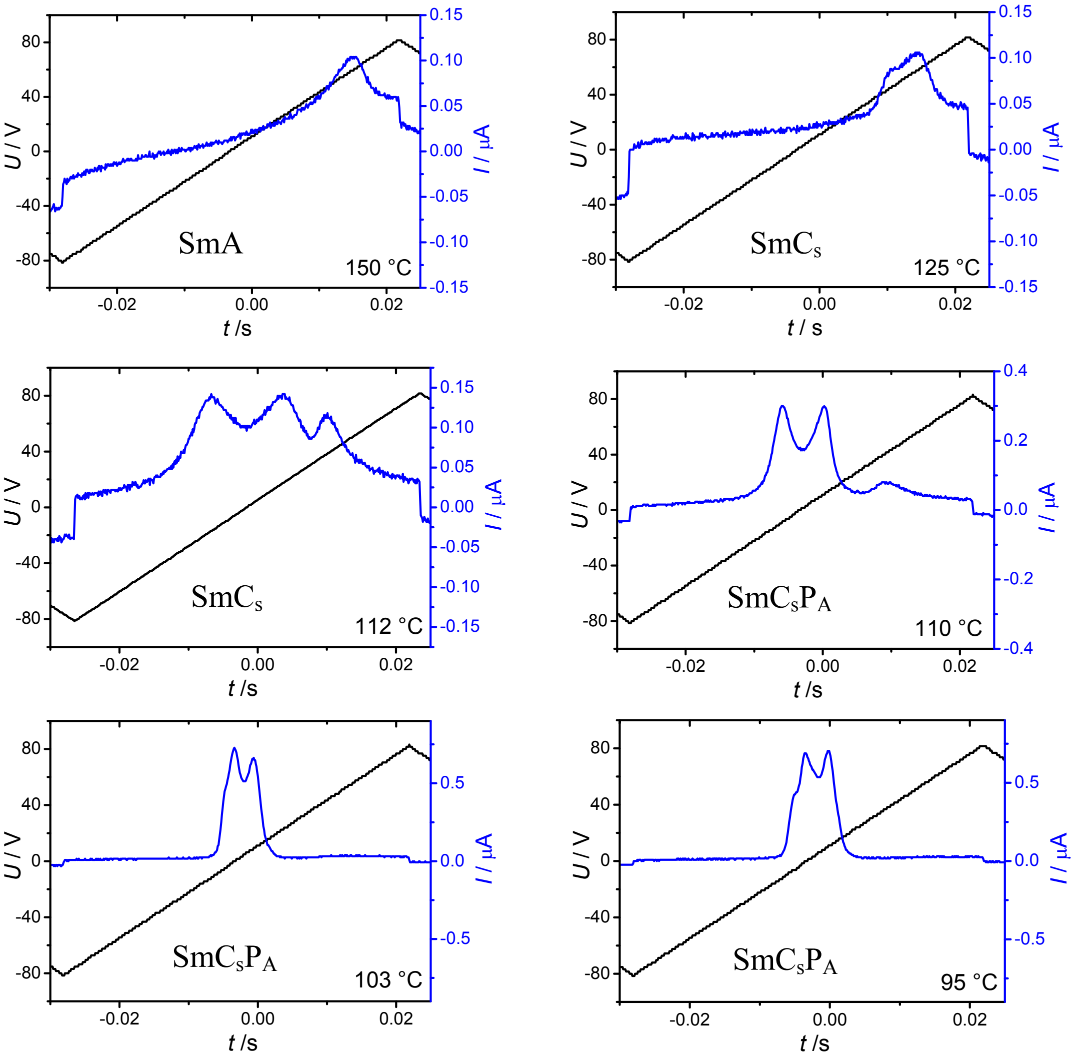

Figure S48. Development of the polarization current peaks of **1/20** depending on temperature.

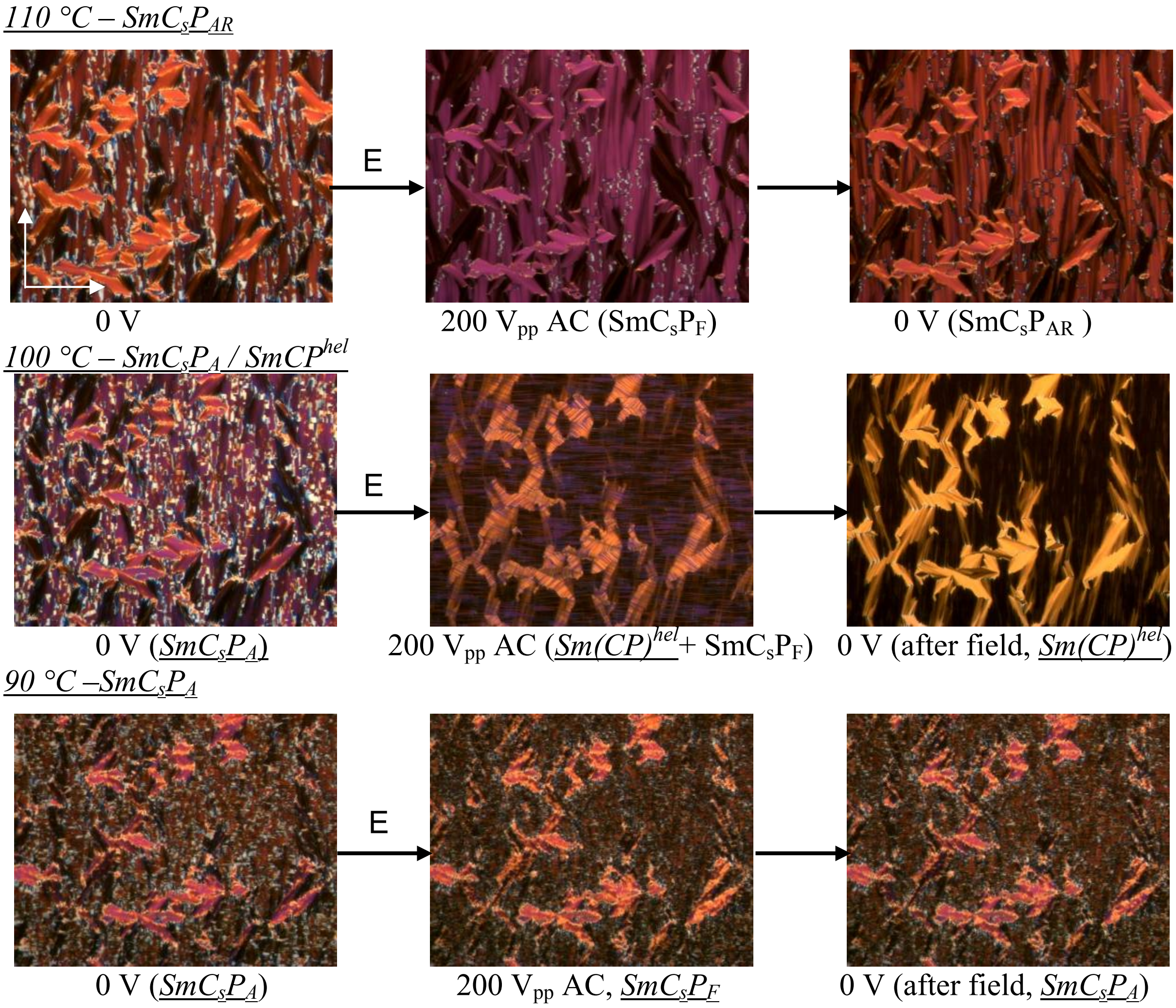

Figure S49. Ground state (left), field induced phases (middle) and textures after relaxation (right) as observed for **1/20** at the indicated temperatures (6 µm PI-coated ITO cell).

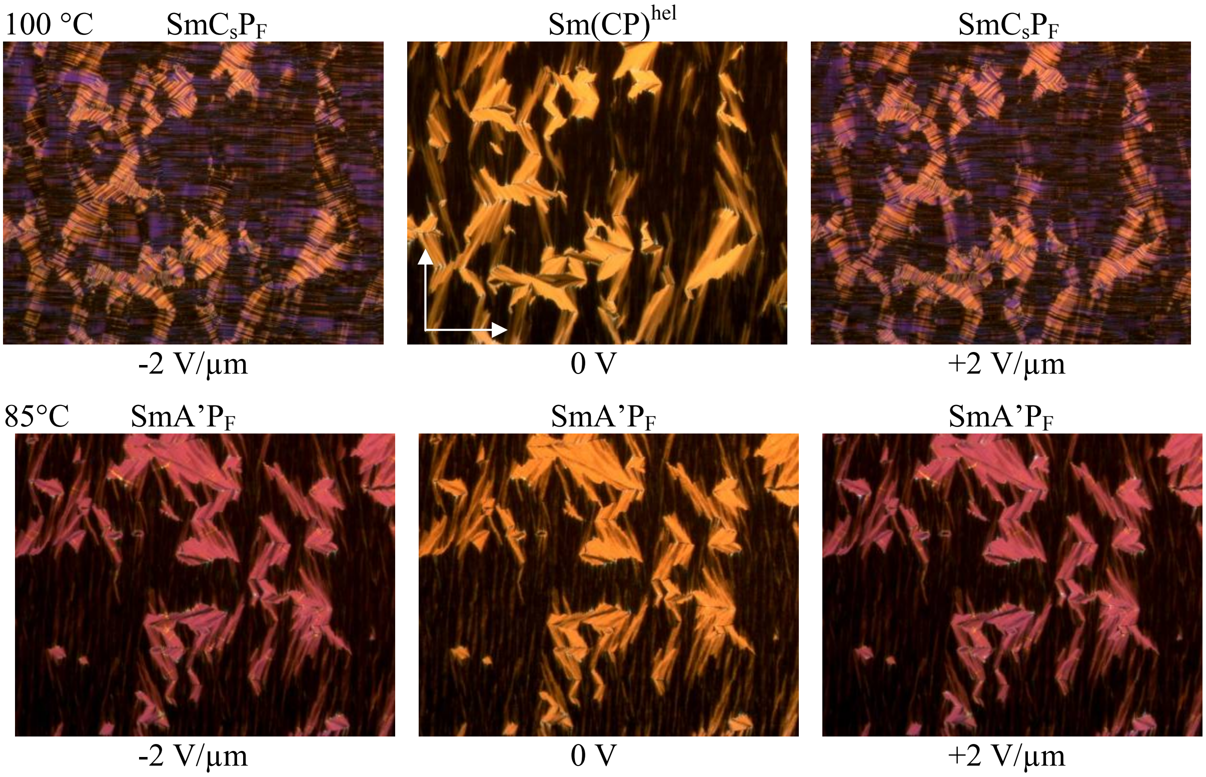

Figure S50. Ground state (middle), field induced phases (right, left) as observed for **1/20** at the indicated temperatures (6 µm PI-coated ITO cell).

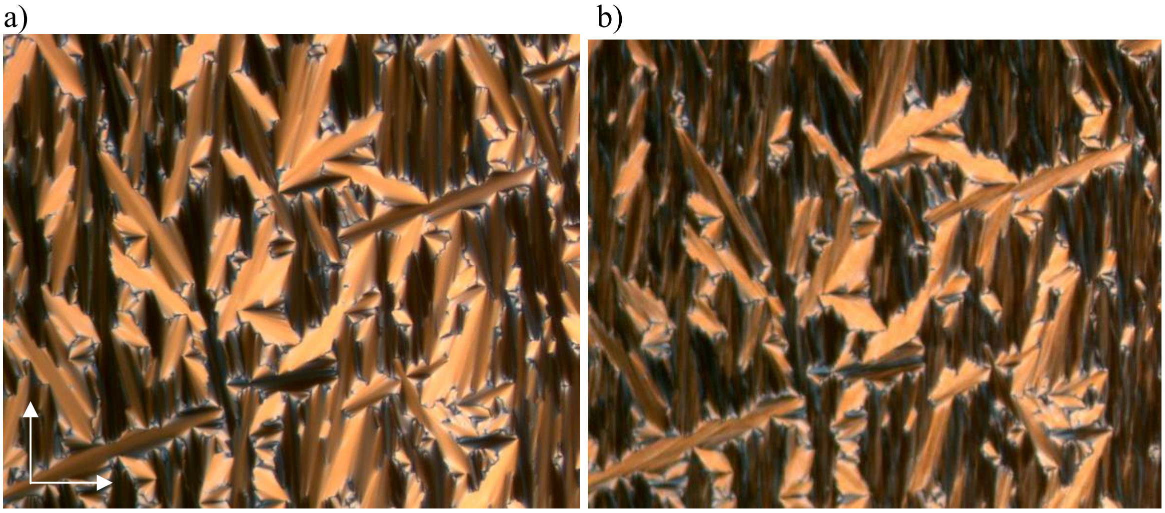

Figure S51. Planar textures of **1/20** a) in the SmA phase at 145 °C and b) the Cr' phase at 80 °C; optically the Cr' phase is almost indistinguishable from the SmA phase, though they have very different transition enthalpy values (see Table S1) and rheological properties, and the crystalline phase show a strong hysteresis of the phase transitions (compare DSCs in Figs. 6c, S43, and S52).

3.10 Compound 1/22

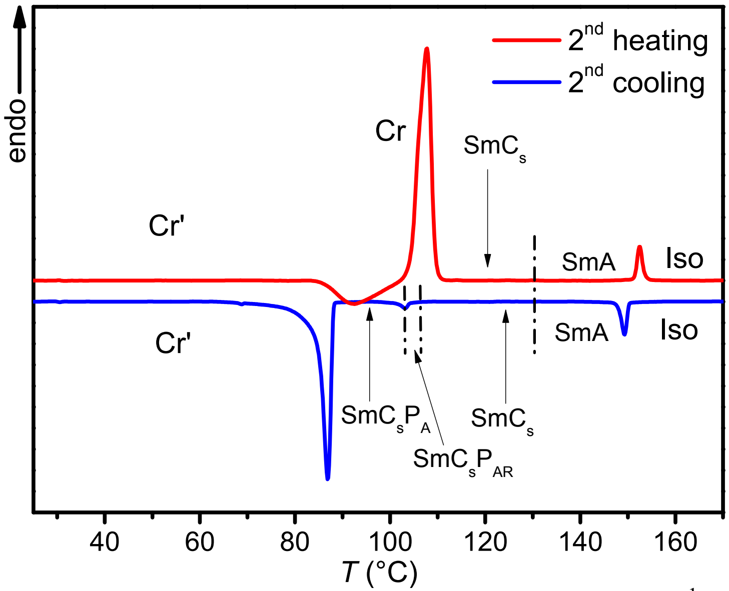

Figure S52. DSC traces of compound **1/22** (10 K min^{-1}).

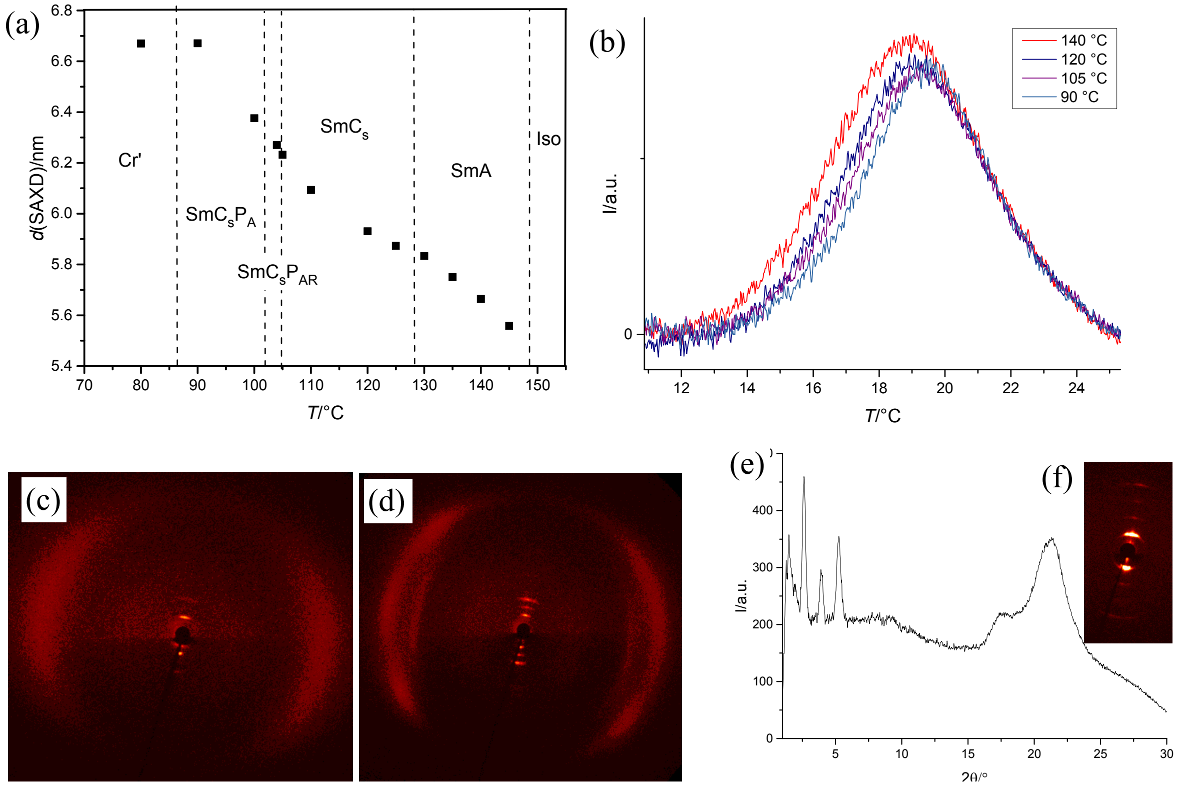

Figure S53. XRD of compound **1/22.** a) Temperature dependence of the *d*-values of the small angle scattering and b) of the wide angle scattering; c) and d) show the 2d diffraction patterns of a surface aligned sample; c) in the SmC_sP_A phase at 90 °C and d) in the Cr' phase at 80 °C. Though, there is no clear indication of a tilt in the SmC_sP_A phase, the out-of-equator scattering maxima in the Cr' phase indicate a tilted organization (by 32-34°), with the outer scattering maxima being similar to the pattern of a hexatic SmI phase; e) the plot of the diffraction pattern of the Cr' phase at 80 °C indicates a well defined lamellar phase showing 4 orders of the layer reflection (the 1st order reflection is partly shadowed by the beam stop and clearly visible in the SAXS pattern in f).

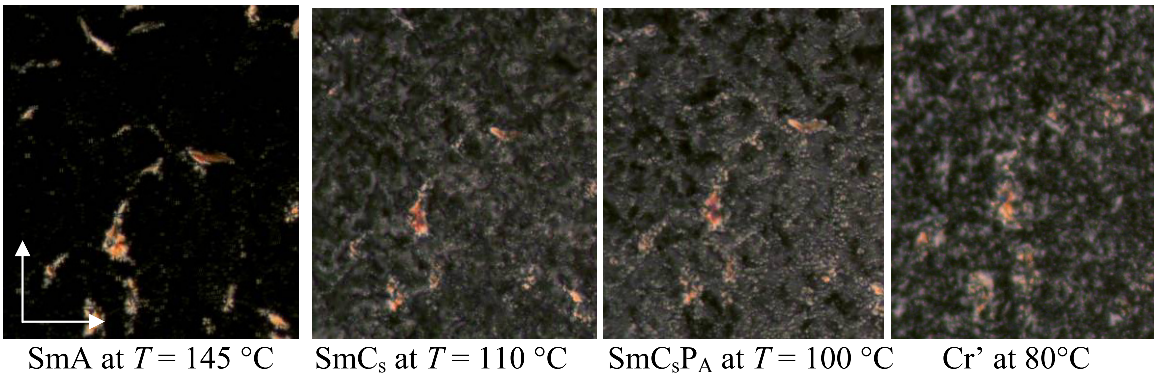

Figure S54. Textures of a predominately homeotropic aligned sample of **1/22** in the distinct phase ranges.

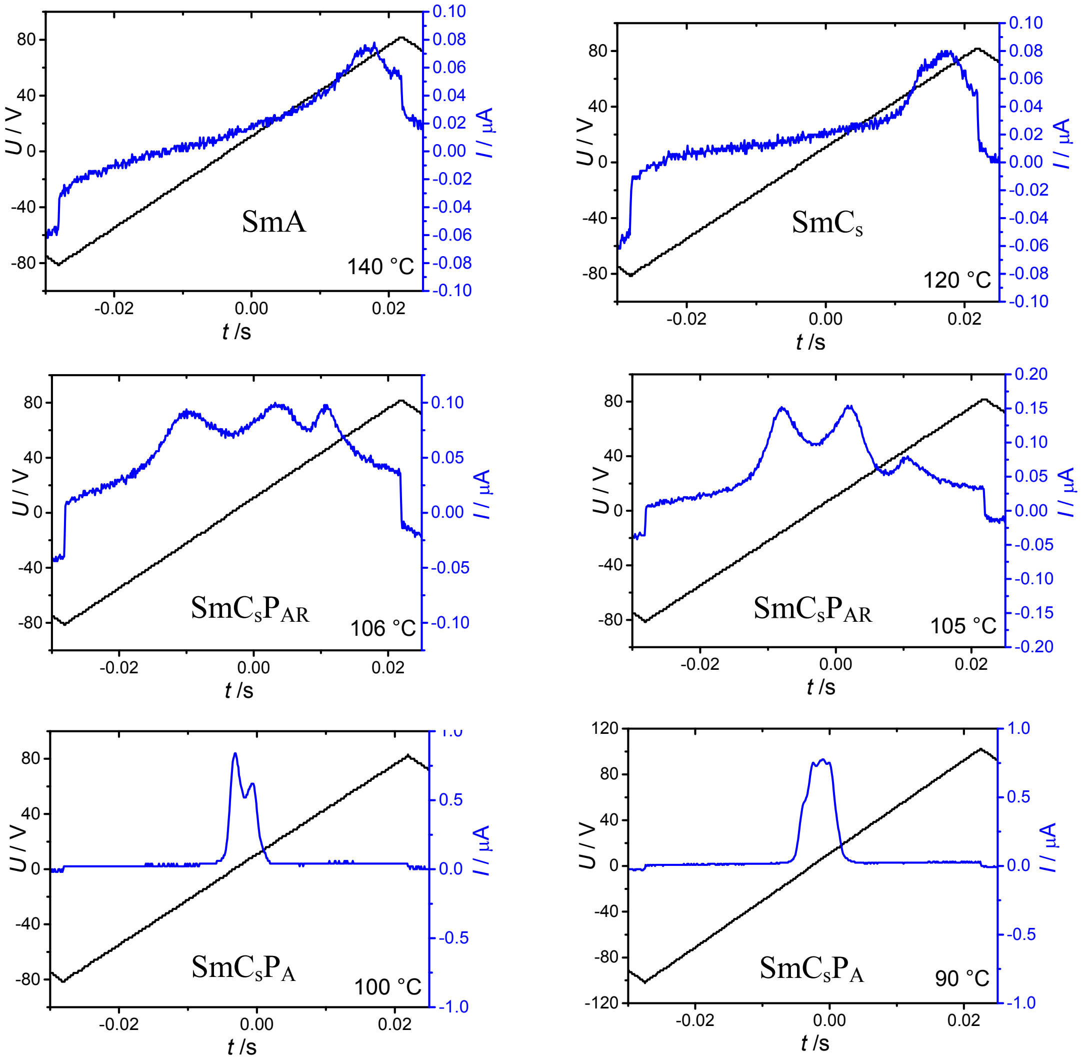

Figure S55. Development of the polarization current peaks of **1/22** depending on temperature.

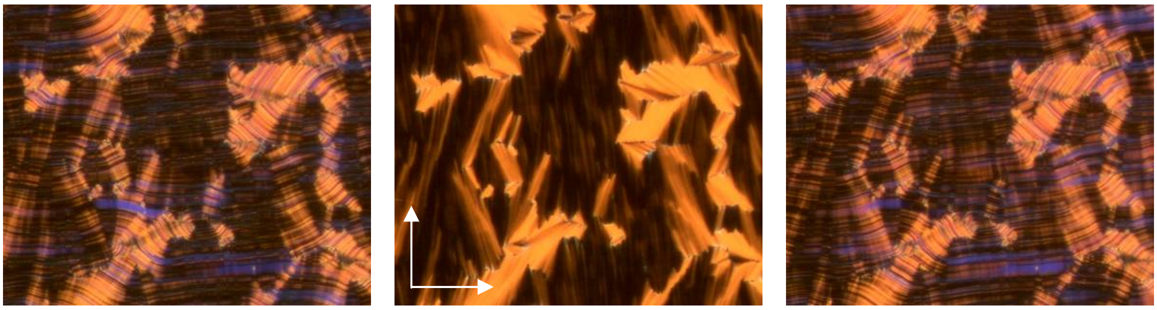

-2 V/µm 0 V +2 V/µm

Figure S56. Switching in the SmC_sP_A phase of **1/22** at T = 105 °C (6 µm, PI-coated ITO cell) under a DC field $Sm(CP)^{hel}$ phase (middle) and the two field induced SmC_sP_F states (left, right) by rotation on a cone.

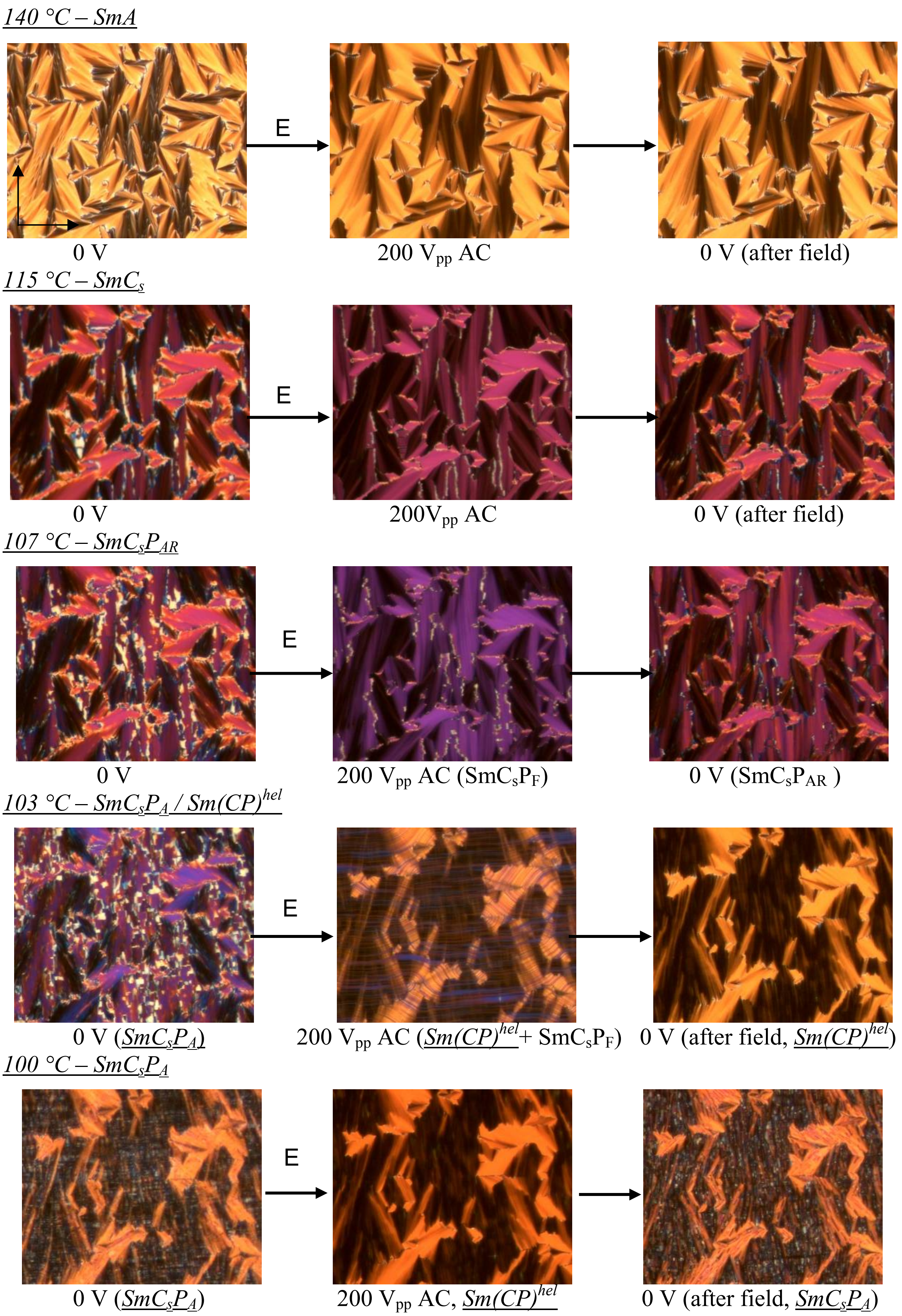

Figure S57. Ground state (left), field induced phases (middle) and textures after relaxation (right) as observed for **1/22** at the indicated temperatures (6 µm PI-coated ITO cell).

4. Development of phase assignments of compounds 1/*n*

As noted in the introduction, XRD gives no indication of any tilt in the LC phases of compounds **1/*n***. In all cases, even for the long chain compounds **1/18-1/22**, for which the tilt can be easily identified for the synclinic tilted SmC phases, there is an almost continuous increase of the *d*-values across the SmA-SmC transitions (see Fig. 7a, and for the individual compounds **1/6** (Fig. S4), **1/8** (Fig. S8), **1/10** (Fig. S10), **1/12** (Fig. S16), **1/16** (Fig. S27), **1/18** (Fig. S33a), **1/20** (Fig. S44) and **1/22** (Fig. S53). Even in aligned samples the tilt cannot be detected, as shown in Fig. S28 for compound **1/16** as example. The deviation of the position of the small angle maximum is only +1…+2° from the meridional (180 °) position and the maxima of the wide angle scatterings deviate only by -2 … -3° from the equatorial 90 and 270° positions, giving a possible tilt of only 3-5°, being in the range of the error of the method (±5°). For this reason and because the tilt is anticlinic in the SmC phases (and the switching in most cases takes place by rotation around the long axis), the polar smectic phases of compounds **1/6**, **1/8**, **1/12** and **14/14** have in previous work been described as non-tilted,[S4,S8-S18] which was later corrected,[S5,S19-S23] after recognition of the tilted organization of **1/14**,[S24,S21] **1/16**, **1/18**[S5] and related alkoxy-substituted compounds.[S25] Table S2 summarizes the development of the phase designations for all previously reported compounds **1/*n***.

Figure S2. Development of the phase designations in the series **1/*n***; the phase sequences given in this work (Tables 1 and S1) are shown in red.

1/*n*	Phase sequence on cooling								Ref.
**1/6**	Iso	SmA				SmAP$_A$		Cr	S4
	Iso	SmA			SmAP$_{AR}$	SmC$_a$P$_A$		Cr	this work
**1/8**	Iso	SmA				SmAP$_A$		Cr	S12,S14,S16
	Iso	SmA			SmAP$_{AR}$	SmC$_a$P$_A$		Cr	this work
**1/12**	Iso	SmA		SmAP$_R$[a] (SmAP$_Z$)[b]		SmAP$_A$		Cr	S4,S8,S9, S12,S13,S16
	Iso	SmA		SmAP$_R$		SmC$_a$P$_A$		Cr	this work
**1/14**	Iso	SmA		SmAP$_R$ (SmAP$_Z$)[b]	SmAP$_{AR}$(SmAP$_\alpha$)[c]	SmAP$_A$		Cr	S11,S12
	Iso	SmA		SmAP$_R$	SmAP$_\alpha$ (SmAP$_x$)[d]	SmAP$_A$		Cr	S10
	Iso	SmA			Sm(CP)$_\alpha$	SmC$_a$P$_A$	SmC$_a$P$_F$	Cr	S21
	Iso	SmA		SmAP$_R$	Sm(CP)^{hel}	SmC$_a$P$_A$$^{(hel)}$	SmC$_a$P$_A$	Cr	this work
**1/16**	Iso	SmA	SmA$_b$	SmAP$_R$	SmAP$_{AR}$	SmAP$_A$		Cr	S15,S17
	Iso	SmA	SmC$_s$[e]	SmC$_S$P$_F$^{hel}		SmCP$_A$[f]		Cr	S5
	Iso	SmA	SmC$_s$P$_R$	SmC$_S$P$_F$^{hel}		SmC$_a$P$_A$		Cr	S19
	Iso	SmA	SmC$_s$[e]	SmC$_S$P$_F$^{hel}		SmCP$_A$[f]		Cr	S20
	Iso	SmA	SmCP$_R$[e]	SmC$_S$P$_F$^{hel}		SmC$_a$P$_A$		Cr	S22
	Iso	SmA	SmC$_s$P$_R$	SmC$_S$P$_F$^{hel}		SmC$_a$P$_A$[i]	SmAP$_A$	Cr	S23
	Iso	SmA	SmC$_x$P$_R$[k]	Sm(CP)^{hel}		SmC$_a$P$_A$[h]	SmA'P$_F$	Cr	this work
**1/18**	Iso	SmA	SmC$_s$P$_R$	SmC$_s$P$_F$				Cr	S18
	Iso	SmA	SmC$_S$[e]	SmC$_s$P$_F$[j] / SmC$_S$P$_F$^{hel}[g]		SmCP$_A$[f]		Cr	S5
	Iso	SmA	SmC$_s$P$_R$	SmC$_s$P$_F$[j] / SmC$_S$P$_F$^{hel}[g,h]		SmC$_a$P$_A$		Cr	S19,S22
	Iso	SmA	SmC$_s$P$_R$	SmC$_s$P$_A$ / Sm(CP)^{hel}[g]			SmA'P$_F$	Cr	this work

For general abbreviations of the phases, see Table 1; additional abbreviations are explained here: SmA$_b$ = biaxial SmA phase; [a] there is no transition enthalpy for the SmA-SmAP$_R$ transition as mistakenly stated in ref. S4; the correct DSC of **1/12** is shown in Fig. 6b; [b] used as tentative phase abbreviation for SmAP$_R$ in ref. S12; [c] proposed phase structure in ref. S11; [d] used as tentative phase abbreviation for SmAP$_R$ in ref. S10; [e] the paraelectric SmC$_s$ phases were not in all cases designated as SmC$_s$P$_R$ or SmC$_s$P$_R$[*]; [f] SmCP$_A$ = SmC$_a$P$_A$; [g] field induced phase; [h] an additional small (0.5 K) range of a SmC$_s$P$_X$ phase is observed between SmC$_S$P$_F$^{hel} and SmC$_s$P$_R$ at 110-111 °C; for details, see ref. S19; [i] with surface stabilized SmC$_a$P$_F$ state; [j] SmC$_s$P$_F$ is the field-induced structure after removal

of the helix; [k] the precise structure of SmC_xP_R is still unknown, but it appears to be a polarization randomized, probably heliconical phase.

In a recent report it was shown that at lower temperature the SmC_aP_A phase of **1/16** is replaced by a non-tilted smectic phase, designated as $SmAP_A$.[S23] Herein we propose that this phase should actually be considered as a ferroelectric $SmAP_F$ ($SmA'P_F$) phase. Thus, the situation concerning the tilt in the distinct polar lamellar phases is not simple at all.

Concerning the history of the heliconical phases, the first indication of a heliconical phase structure in bent mesogens actually dates back to 2011 when it was, based on XRD evidences, reported to represent a non-tilted helical organization of the molecules in the uniaxial and polar smectic phase of compound **1/14**, and it was therefore named $SmAP_\alpha$;[S10] after recognition of the tilted organization[S24] the phase abbreviation was changed to $SmCP_\alpha$[S25b] and $Sm(CP)_\alpha$[S21] and since 2016 $SmC_sP_F^{hel}$ was used.[S5,S19] In 2018 the first report on related heliconical phases with a helix being commensurate with the layer distance, formed by a mesogenic dimer, appeared which was designated as SmC_{TB}.[S 26] The designations $SmCP_\alpha$/$Sm(CP)_\alpha$ for the bent-core molecules were chosen due to the similarity with the incommensurate SmC_α* phases of chiral mesogens,[S21] and $SmC_sP_F^{hel}$ indicates that this phase actually represents a structure resulting from the escape of the macroscopic polar order of synclinic tilted polar SmC_sP_F layers by adopting a heliconical superstructure.[S5] Herein we prefer to use $Sm(CP)^{hel}$ as a general phase assignment of heliconical smectic phases, also including possible commensurate phase types.

5. References

S1 W. Steglich, G. Höfle, N,N-Dimethyl-4-pyridinamin, a Very Effective Acylation Catalyst, *Angew. Chem. Int. Ed. Engl.*, **1969**, *8*, 981; B. Neises, W. Steglich, Simple Method for the Esterification Carboxylic Acids, *Angew. Chem. Int. Ed.*, **1978**, *17*, 522; A. C. Spivey, S. Arseniyadis, Nucleophilic Catalysis by 4 - (Dialkylamino)pyridines Revisited—The Search for Optimal Reactivity and Selectivity, *Angew. Chem. Int. Ed.*, **2004**, *43*, 5436-5441; C. Tschierske, H. Zaschke, A Mild and Convenient Esterification of Sensitive Carboxylic Acids, *J. prakt. Chem.*, **1989**, *331*, 365-366.

S2 J. L. Serrano, T. Sierra, Y. Gonzalez, C. Bolm, K. Weickhardt, A. Magnus, G. Moll, Improving FLC Properties. Simplicity, Planarity, and Rigidity in New Chiral Oxazoline Derivatives, *J. Am. Chem. Soc.* **1995**, *117*, 8312.

S3 M. Alaasar, M. Prehm, M. Brautzsch, C. Tschierske, 4-Methylresorcinol based bent-core liquid crystals with azobenzene wings – a new class of compounds with dark conglomerate phases, *J. Mater. Chem. C*, **2014**, *2*, 5487–5501.

S4 **1/6, 1/12**: C. Keith, M. Prehm, Y. P. Paranin, J. K. Vij, C. Tschierske, Development of polar order in liquid crystalline phases of a banana compound with a unique sequence of three orthogonal phases, *Chem. Commun.*, **2010**, *46*, 3702-3704.

S5 **1/16, 1/18**: S. P. Sreenilayam, Y. P. Panarin, J. K. Vij, V. P. Panov, A. Lehmann, M. Poppe, M. Prehm, C. Tschierske, Spontaneous helix formation in non-chiral bent-core liquid crystals with fast linear electro-optic effect, *Nat. Commun.*, **2016**, *7*, 11369.

S6 R. Achten, A. Koudijs, M. Giesbers, A. T. M. Marcelis, E. J. R. Sudhölter, Non-symmetric bent-core mesogens with one terminal vinyl group, *Liq. Cryst.*, **2005**, *32*, 277.

S7 R. Achten, R. Cuypers, M. Giesbers, A. Koudijs, A. Marcelis, E. Sudholter, Asymmetric banana-shaped liquid crystals with two different terminal alkoxy chains, *Liq. Cryst.*, **2004**, *31*, 1167.

S8 **1/12**: Y. P. Panarin, M. Nagaraj, J. K. Vij, C. Keith, C. Tschierske, Field-induced transformations in the biaxial order of non-tilted phases in a bent-core smectic liquid crystal, *EPL*, **2010**, *92*, 26002.

S9 **1/12**: M. Nagaraj, Y.P. Panarin, J. K. Vij, C. Keith, C. Tschierske, Liquid crystal display modes in a nontilted bent-core biaxial smectic liquid crystal, *Appl. Phys. Lett.*, **2010**, *97*, 213505.

S10 **1/14**: Y. P. Panarin, N. Nagaraj, S. Sreenilayam, J. K. Vij, A. Lehmann, C. Tschierske, Sequence of Four Orthogonal Smectic Phases in an Achiral Bent-Core Liquid Crystal: Evidence for the $SmAP_\alpha$ Phase, *Phys. Rev. Lett.*, **2011**, *107*, 247801.

S11 **1/14**: S. Sreenilayam, N. Nagaraj, Y. P. Panarin, J. K. Vij, A. Lehmann, C. Tschierske, Structure and Polymorphism of Biaxial Bent-Core Smectic Liquid Crystals , *Mol. Cryst. Liq. Cryst.*, **2012**, *553*, 133-139.

S12 **1/8, 1/12, 1/14**: S. Sreenilayam, N. Nagaraj, Y. P. Panarin, J. K. Vij, A. Lehmann, C. Tschierske, Properties of Non-Tilted Bent-Core Smectic Liquid Crystals, *Mol. Cryst. Liq. Cryst.*, **2012**, *553*, 140-146.

S13 **1/12**: N. Nagaraj, S. Sreenilayam, Y. P. Panarin, J. K. Vij, C. Keith, C. Tschierske, Electric field induced transformations and dielectric properties in non-tilted phases of a bent-core smectic liquid crystal, *Mol. Cryst. Liq. Cryst.*, **2011**, *540*, 82-87.

S14 **1/8**: S. Sreenilayam, Y. P. Panarin, J. K. Vij, A. Lehmann. C. Tschierske, Biaxial Order Parameter in an Achiral Bent-Core Smectic Liquid Crystals, *Ferroelectrics*, **2012**, *431*, 190-195.

S15 **1/16**: S. Sreenilayam, Y. P. Panarin, J. K. Vij, A. Lehmann, C. Tschierske, Physical Properties of SmAb Phase in an Achiral Bent-Core Smectic Liquid Crystals, *Ferroelectrics*, **2012**, *431*, 196-201.

S16 **1/12, 1/14, 1/18**: S. Sreenilayam, Y. P. Panarin, J. K. Vij, M. Osipov, A. Lehmann, C. Tschierske, Biaxial order parameter in the homologous series of orthogonal Bent-Core Smectic Liquid Crystals, *Phys. Rev. E*, **2013**, *88*, 012504.

S17 **1/16**: S. Sreenilayam, Yu. P. Panarin, J. K. Vij, A. Lehmann, C. Tschierske, Occurrence of Five Different Orthogonal Smectic Phases in a Bent-Core (BC) Liquid Crystal, *Mol. Cryst. Liq. Cryst.*, **2015**, *610*, 116–121.

S18 **1/18**: S. Sreenilayam, Y. P. Panarin, J. K. Vij, A. Lehmann, C. Tschierske, Fast linear electrooptic effect in nonchiral bent-core liquid crystal, *Ferroelectrics*, **2016**, *495*, 35−42.

S19 **1/16, 1/18**: S. P. Sreenilayam, Yu. P. Panarin, J. K. Vij, A. Lehmann, M. Poppe, C. Tschierske Development of ferroelectricity in the smectic phases of 4-cyanoresorcinol derived achiral bent-core liquid crystals with long terminal alkyl chains, *Phys. Rev. Mater.*, **2017**, *1*, 035604.

S20 **1/16**: Y. P. Panarin, S. P. Sreenilayam, J. K. Vij, A. Lehmann, C. Tschierske, A fast linear electro-optical effect in a non-chiral bent-core liquid crystal, *J. Mater. Chem. C,* **2017**, *5*, 12585.

S21 **1/14**: A. A. S. Green, M. R. Tuchband, R. Shao, Y. Shen, R. Visvanathan, A. E. Duncan, A. Lehmann, C. Tschierske, E. D. Carlson, E. Guzman, M. Kolber, D. M. Walba, C. S. Park, M. A. Glaser, J. E. Maclennan, N. A. Clark, Chiral Incommensurate Helical Phase in a Smectic of Achiral Bent-Core Mesogens, *Phys. Rev. Lett.*, **2019**, *122*, 107801.

S22 **1/16, 1/18**: J. K. Vij, Yu. P. Panarin, S. P. Sreenilayam, M. Alaasar, C. Tschierske, Investigation of the heliconical smectic SmCsPFhel phase in achiral bent-core mesogens derived from of 4-cyanoresorcinol, *Phys. Rev. Mater.*, **2019**, *3*, 045603.

S23 **1/16, 1/18**: Yu. P. Panarin, S. P. Sreenilayam, V. Swaminathan, C. Tschierske, J. K. Vij, Unexpected observation of an anomalous SmA-SmC-SmA phase sequence in a bent-

core liquid crystal derived from 4-cyanoresorcinol, *Phys. Rev. Res*., WHR1005W, accepted.

S24 J. McLeannen, N. Clark, private communication at 24th International Liquid Crystal Conference **2012** (Mainz).

S25 a) C. Tschierske, A. Lehmann, M. Prehm, J. K. Vij, M. Nagaraj, Y. P. Panarin, H. Ocak, B. Bilgin-Eran, M. Alaasar, Molecular Design at the Cross-over Between Rod-like and Bent-core Mesogens: 4-Cyanoresorcinol Derivatives, 24th International Liquid Crystal Conference **2012** (Mainz); b) M. Alaasar, M. Prehm, M. Poppe, M. Nagaraj, J. K. Vij, C. Tschierske, Development of polar order and tilt in lamellar liquid crystalline phases of a bent-core mesogen, *Soft Matter*., **2014**, *10*, 5003−5016; c) N. Sebastian, S. Belau, A. Eremin, M. Alaasar, M. Prehm, C. Tschierske, Emergence of polar order and tilt in terephthalate based bent-core liquid crystals, *Phys.Chem.Chem.Phys*., **2017**, *19*, 5895−5905.

S26 J. P. Abberley, R. Killah, R. Walker, J.M.D. Storey, C. T. Imrie, M. Salamonczyk, C. Zhu, E. Gorecka, D. Pociecha, Heliconical smectic phase formed by achiral molecules, *Nat. Commun*., 2018, **9**, 228.
